# Multiple independent *L-gulonolactone oxidase* (*GULO*) gene losses and vitamin C synthesis reacquisition events in non-Deuterostomian animal species

**DOI:** 10.1186/s12862-019-1454-8

**Published:** 2019-06-18

**Authors:** Sílvia F. Henriques, Pedro Duque, Hugo López-Fernández, Noé Vázquez, Florentino Fdez-Riverola, Miguel Reboiro-Jato, Cristina P. Vieira, Jorge Vieira

**Affiliations:** 1 i3S – Instituto de Investigação e Inovação em Saúde, Universidade do Porto, Porto, Portugal; 2 IBMC – Instituto de Biologia Molecular e Celular, Porto, Portugal; 3 ESEI – Escuela Superior de Ingeniería Informática, Universidade de Vigo, Vigo, Spain; 4 CINBIO - Centro de Investigaciones Biomédicas, University of Vigo, Vigo, Spain; 5 SING Research Group, Galicia Sur Health Research Institute (IIS Galicia Sur), SERGAS - Universidade de Vigo, Vigo, Spain; 6 Centro de Investigaciones Biomédicas (Centro Singular de Investigación de Galicia), Vigo, Spain

**Keywords:** *GULO*, Ascorbic acid, *D. melanogaster*, Microbiome, Synthesis

## Abstract

**Background:**

L-ascorbate (Vitamin C) is an important antioxidant and co-factor in eukaryotic cells, and in mammals it is indispensable for brain development and cognitive function. Vertebrates usually become L-ascorbate auxothrophs when the last enzyme of the synthetic pathway, an L-gulonolactone oxidase (*GULO*), is lost. Since Protostomes were until recently thought not to have a *GULO* gene, they were considered to be auxothrophs for Vitamin C.

**Results:**

By performing phylogenetic analyses with tens of non-Bilateria and Protostomian genomes, it is shown, that a *GULO* gene is present in the non-Bilateria Placozoa, Myxozoa (here reported for the first time) and Anthozoa groups, and in Protostomians, in the Araneae family, the Gastropoda class, the Acari subclass (here reported for the first time), and the Priapulida, Annelida (here reported for the first time) and Brachiopoda phyla lineages. *GULO* is an old gene that predates the separation of Animals and Fungi, although it could be much older. We also show that within Protostomes, *GULO* has been lost multiple times in large taxonomic groups, namely the Pancrustacea, Nematoda, Platyhelminthes and Bivalvia groups, a pattern similar to that reported for Vertebrate species. Nevertheless, we show that *Drosophila melanogaster* seems to be capable of synthesizing L-ascorbate, likely through an alternative pathway, as recently reported for *Caenorhabditis elegans*.

**Conclusions:**

Non-Bilaterian and Protostomians seem to be able to synthesize Vitamin C either through the conventional animal pathway or an alternative pathway, but in this animal group, not being able to synthesize L-ascorbate seems to be the exception rather than the rule.

**Electronic supplementary material:**

The online version of this article (10.1186/s12862-019-1454-8) contains supplementary material, which is available to authorized users.

## Background

L-ascorbate is an essential vitamin in humans. This vitamin has anti-oxidant properties, and in humans its consumption correlates with an increased protection against degenerative diseases and cancer [1], although it may exhibit a pro-oxidative effect at high doses [2, 3]. It is also a well-studied co-factor of enzymes involved in collagen synthesis, facilitates the differentiation of dopaminergic neurons in vitro*,* and is known to be indispensable for proper fetal brain development and cognitive function [4–7]. More recently, it has been shown that L-ascorbate is also a co-factor of enzymes involved in DNA or histones demethylation (e.g. TET1 and JMJD3), thus acting as a modulator of epigenetic modifications [6].

Despite the biological importance of Vitamin C, several mammal species such as primates, guinea pig, and some bat species, have lost the ability to synthesize this vitamin due to the loss of a functional *GULO* gene. This gene encodes a L-gulonolactone oxidase that is involved in the last step of the Vitamin C synthesis pathway [8, 9]. This is the reason why, in humans, a diet that is deficient in Vitamin C, leads to the lethal deficiency disease scurvy. Animal species where the *GULO* gene has been lost usually retain the remaining genes of the pathway, including the *Regucalcin/SMP30* (*Senescence Marker Protein 30*) gene, which encodes a gluconolactonase that produces GULO’s substrate [8, 10]. This observation suggests that the other genes of the pathway play critical functions. For example, in mammals, Regucalcin is a regulator of cellular Ca^2+^ levels, several nuclear processes, and also acts as a transcription factor [11, 12].

Mammals are not the only animal group where *GULO* gene loss has been identified. Indeed, some Passeriformes birds and Teleostei fish species also lack a *GULO* gene. Although Vitamin C could be detected in many species [10, 13–16], most Protostomes were thought not to have a *GULO* gene. Nevertheless, recently, the *GULO* gene has been identified in the annotated genomes of five Araneae, Priapulida, Brachiopoda and Gastropoda species [17]. Therefore, the *GULO* gene has not been completely lost in Protostomian species. There are, however, three Protostomian lineages where a *GULO* gene has not been detected, although a large number of genomes has been analysed, namely the Insects (116 genomes), Nematoda (35 genomes) and Platyhelminthes (nine genomes) [17]. In non-Bilateria, the *GULO* gene has been found in Anthozoan and Placozoan species [10, 17], showing that, in animals, having a *GULO* gene is the ancestral state. In this work we present evidence that *GULO* is also present in the Myxozoa (non-Bilaterian), Acari and Annelida groups (Protostomian), and thus that *GULO* is present in most non-Bilaterian and Protostomian lineages.

Despite the observation that there is no *GULO* gene in insects, there is little doubt that Vitamin C is important in this group of species. For instance, in *D. melanogaster*, L-ascorbate levels increase during aging, and Vitamin C supplemented food contributes to the extension of the average and the maximum life-span of flies [3, 16]. The higher levels of L-ascorbate found in the midgut lumen of the *Orgyia leucostigma* moth are believed to be responsible for the superior tolerance of this species against tannin, a ROS-inducing agent [18]. In beetles (*Callosobrochus maulatus*), L-ascorbate levels decrease in a dose-dependent manner in response to the presence of different ROS-inducing insecticides, possibly by aiding enzymatic antioxidant systems such as catalase, superoxide dismutase (SOD) and peroxidase (POX) [19]. In ladybirds (*Cryptolaemus montrouzieri Mulsant*), the genes related to L-ascorbate metabolism were found to be specifically altered in response to cold-stress, but not in response to heat-stress [20].

Although there is no *GULO* gene in the nematode *C. elegans*, this species was recently shown to be able to synthesize L-ascorbate from its food source, *Escherichia coli* [21]. Therefore, it has been proposed that *C. elegans* uses an alternative enzyme, or pathway, to synthetize L-ascorbate, although the gene responsible for it has not been identified [21]. This observation raises the possibility that in insects, such as *D. melanogaster*, an alternative pathway for the synthesis of Vitamin C is also present. Indeed, it has been reported that flies raised in the absence of L-ascorbate, have increased levels of Vitamin C in response to a brief cold shock (10 min, 4 °C) [16]. It could, however, be argued that the source of Vitamin C is the microbiome. This is a possibility because, although it is usually considered that prokaryotes do not depend on, or synthetize, Vitamin C, several L-ascorbate-producing bacteria that live in symbiosis with metazoan hosts have been identified, such as *Mycobacterium tuberculosis*, *Pantoea citrea* and a particular strain of *Pseudomonas aeruginosa* that colonizes the human host [22–25]. Moreover, it has been shown that in *D. melanogaster*, the microbiome is able to provide vitamins (such as Vitamin B_1_) in amounts that can support the viability of its offspring [26]. Therefore, in this work we also address whether Vitamin C is synthesized in *Drosophila* by the microbiome or by the fly. We also looked at whether, as suggested by the brief cold exposition experiments performed by Massie et al. [16], Vitamin C levels increase under the cold-acclimation conditions usually used in the literature (one day at 15 °C). This experiment could provide further evidence for Vitamin C synthesis, since cold acclimation induces oxidative stress [27], and Vitamin C is a well-known anti-oxidant [1]. Therefore, its production could be regulated by the fly when exposed at low temperatures. We show that in non-Bilateria as well as in Protostomia, not being able to synthesize Vitamin C is likely the exception rather than the rule.

## Results

### Non-Bilateria and Protostomia species have putative functional *GULO* orthologs

The implementation of a fast an efficient protocol for the identification of *GULO* gene in annotated genomes led to the identification of this gene in 118 species, in groups such as the Anthozoa (non-Bilaterian), Araneae, Priapulida, Brachiopoda and Gastropoda (Protostomians) [17]. This result motivated us to use non-annotated genomes of further non-Bilateria and Protostomia species that belong to phylogenetic groups that were not well represented in [17]. Furthermore, since many taxonomic groups are represented by a reduced number of annotated species genomes only, we decided to perform our own annotations using these genomes to avoid loss of information that could arise from poor annotations that were eventually removed in [17]. The details of the gene annotation procedures here used can be found in supplementary Additional file 1 Table S1.

It should be noted that our de novo *GULO*-like gene annotations lack an ATG start codon. Nevertheless, given the alignments between our gene annotations and the mouse *GULO* gene, it seems that the first *GULO* coding exon codes for the ATG codon only. Therefore, although no start codon was annotated, we are confident that these annotations represent functional genes.

**Fig. 1:**
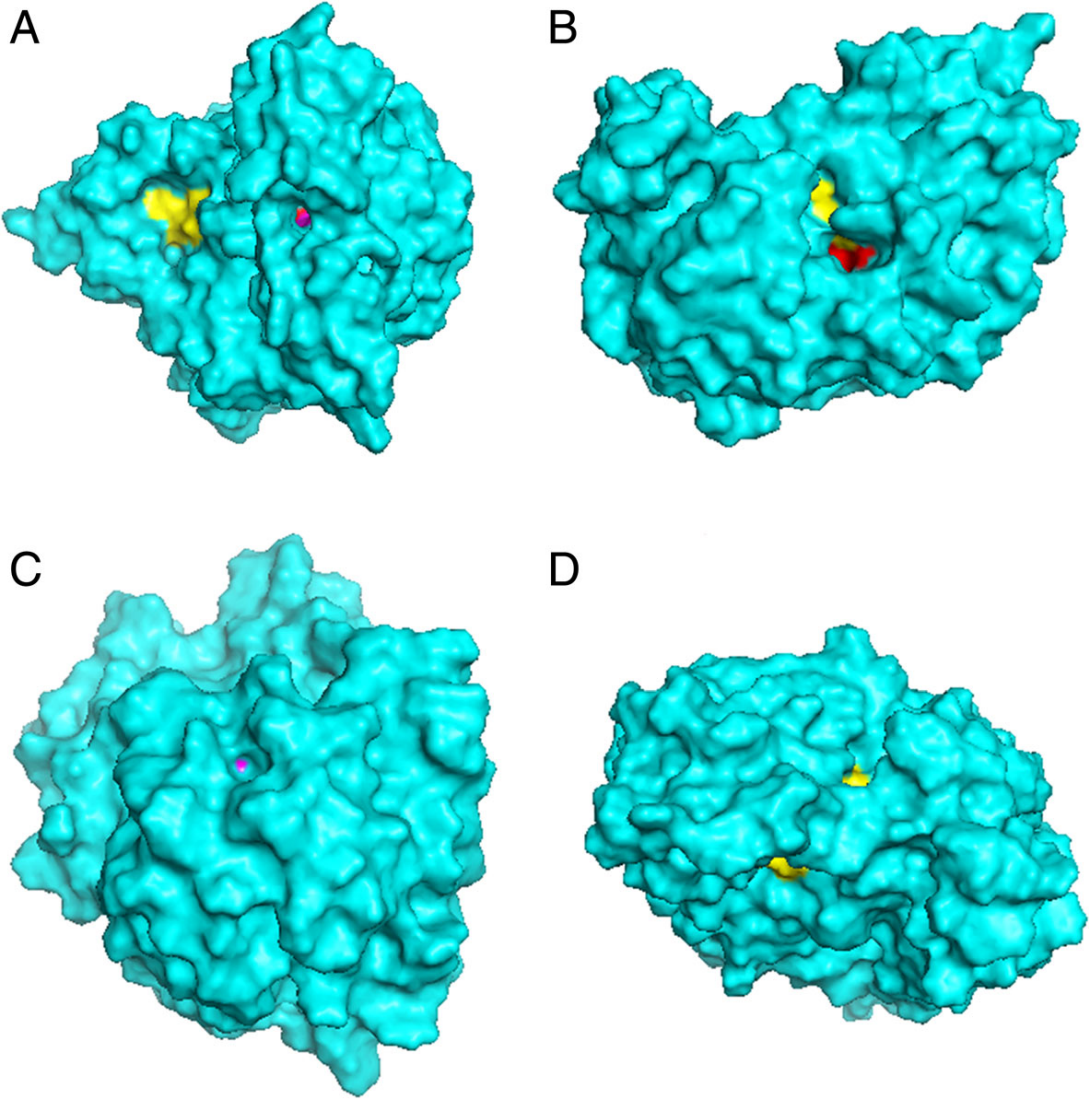
I-TASSER predicted *M. musculus* HWAK (NP_848862.1; panels **a** and **b**) and HWGK GULO (panels **c** and **d**) models. For the HWAK GULO model, in **a**), we can identify an open channel in the protein structure, leading to the exposed HWAK motif (red) and the adjacent Glu-Arg pair (magenta). Furthermore, we can also identify the FAD-binding motif at the surface (yellow). In **b**), we can identify the surface contact point between the HWAK (red) and FAD-binding (yellow) motifs. As for the HWGK GULO model, in **c**), we can observe the single protein surface point were the Glu-Arg pair is located (magenta). In **d**), we can see the two exposure points of the conserved FAD-binding domain

For the phylogenetic analysis only sequences harbouring the HWXK motif were considered, since this motif is conserved amongst members of the vanillyl-alcohol oxidase (VAO) flavoproteins family that includes besides *GULO* the genes that encode plant L-galactono-1,4-lactone dehydrogenases (GLDH) and fungi D-arabinono-1,4-lactone oxidases (ALO) that synthetize L-ascorbate and D-erythorbate, respectively [28]. Ten CDSs from non-Bilateria and Protostomia species here annotated, that appear as an external group to the fungi sequences in the consensus phylogeny (Additional file 2 Figure S1), likely represent other genes that also contain a FAD-domain [28]. Interestingly, all *GULO* CDS identified in [17] as well as those that here cluster with the reference CDSs from animals were found to encode proteins with the HWAK motif, while the CDSs outside the fungi outgroup, with the exception of those from *R. microplus* and *K. iwatai,* encode proteins with the HWGK motif (Additional file 2 Figure S1). The sequence from *R. microplus* seems to be a contamination, since it has high homology to a CDS from a bacterial species (data not shown). The CDS from *K. iwatai* was maintained in our dataset since it clustered with the other sequences encoding proteins with a HWGK motif with low posterior credibility support value (Additional file 2 Figure S1). These observations strongly suggest that, in animals, true *GULO* orthologs always encode proteins with the HWAK motif. Currently, there is no protein structure available for GULO that we can use to address the impact of the Alanine methyl group (−CH3) that is absent in Glycine at the HWXK motif. Therefore, we relied on the in silico predictions obtained with the I-TASSER server. The predicted structure of the *M. musculus* GULO (NP_848862.1) is shown in Fig. 1A, where it can be seen that the HWAK conserved motif is exposed at the surface, and that it is adjacent to the Glu-Arg (Glutamic acid and Arginine) pair, known to be involved in optimal catalysis by aldonolactone oxidoreductases [28]. Furthermore, the GULO FAD-binding domain is also exposed at various sites, one of which in the proximity of the HWAK motif region (Fig. 1B). The *M. musculus* GULO G111 amino acid, implicated in the ability to use molecular oxygen as an electron acceptor in aldonolactone oxidation reactions [29, 30] is located in an internal region of the protein. When we change the alanine of the HWAK motif to a glycine, and repeat the analysis, the HWGK motif is now in an internal region of the protein (Fig. 1C). The Glu-Arg pair is now barely at the surface as well. The FAD-binding region is still well exposed at the protein surface, in two separate regions, while the G111 amino acid remains unexposed (Fig. 1D). Therefore, a single amino acid change is predicted to cause a drastic conformational change, further suggesting that the HWAK motif is under strong purifying selection. In the future, the identification of *GULO* genes can thus be easily made in annotated genomes by performing a simple Blast PHI (pattern-hit initiated) search using this motif.

**Fig. 2:**
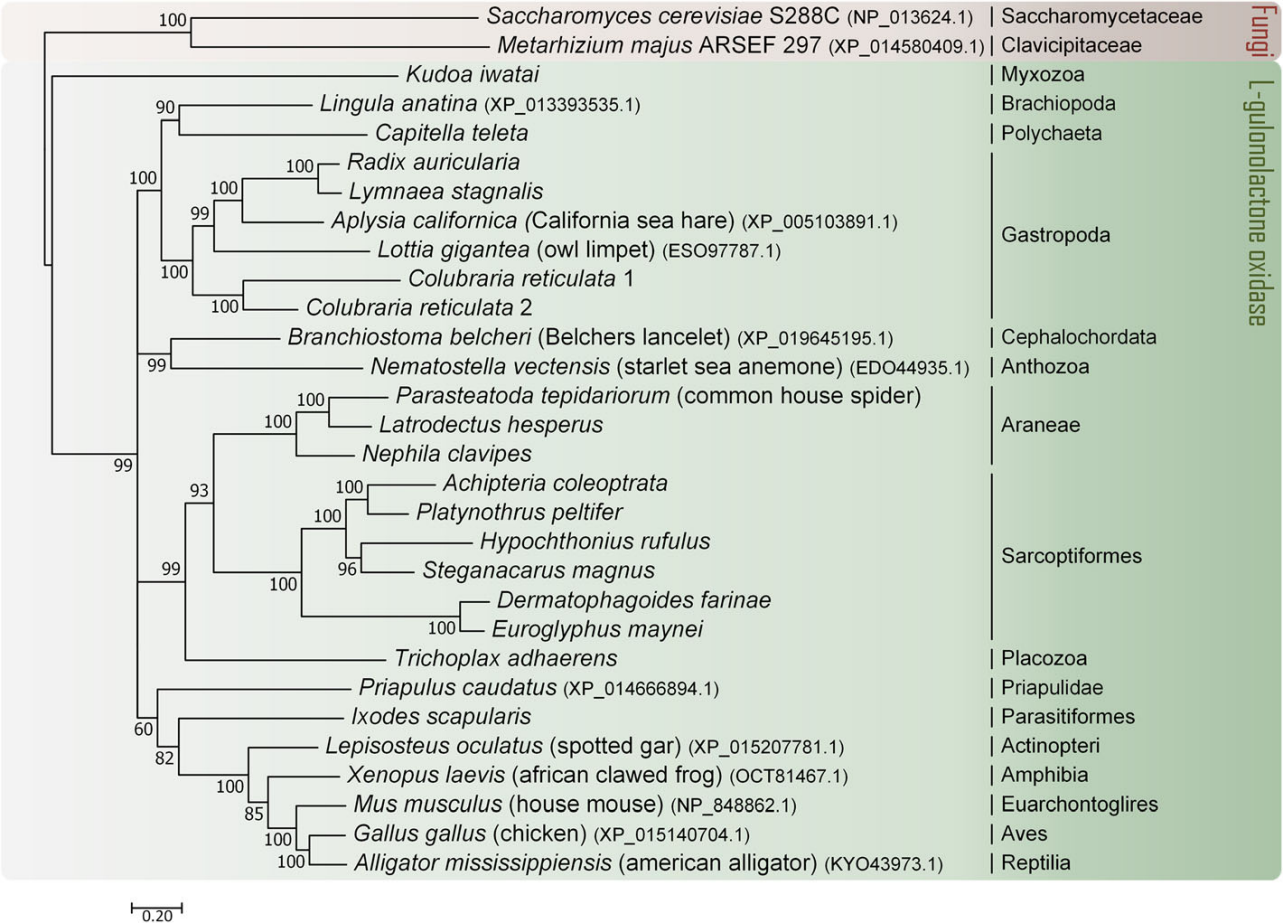
Phylogenetic relationship amongst the putative *GULO* orthologs identified in Protostomian and non-Bilaterian species that have a HWAK motif. Two Fungi *GULO* CDS were used to assist rooting of the consensus tree (red pane). *GULO* CDS of six deuterostomian species, representing the Actinopteri, Amphibia, Euarchontoglires, Aves, Reptilia and Cephalochordata groups, were used to facilitate the identification of functional *GULO* orthologs among Animal species (green pane). Higher taxonomic classifications for common species are shown on the right

Since the sequences coding for proteins without the HWAK motif are distantly related to the *GULO* gene sequences, and thus could have an impact on the phylogenetic reconstruction, we repeated the phylogenetic analysis using only the sequences encoding proteins with this motif (Fig. 2). This phylogenetic analysis clearly shows that, in addition to the groups where a *GULO* gene was previously identified in [17], there is an apparently functional *GULO* gene in Placozoa and Myxozoa (non-Bilaterians) and in Acari and Annelida (Protostomians). Although the results regarding Myxozoa should be regarded with some caution, as the Myxozoan HWAK containing sequence is identified as a GULO sequence only in the phylogenetic tree obtained after excluding HWGK sequences, the presence of the highly specific HWAK GULO motif supports the interpretation that in Myxozoa there is indeed a *GULO* gene. Animals synthesize ascorbic acid starting from D-glucose, and D-glucuronic acid is one of the intermediate products of the vitamin C synthesis pathway. The latter product is used by the pentose phosphate pathway, and thus it is not surprising that the genes that encode the enzymes that catalyse the intermediary steps of the conversion of D-glucose into D-glucuronic acid are essential genes. D-glucuronic acid is also used by Regucalcin to produce L-gulono-gamma-lactone. Then GULO uses L-gulono-gamma-lactone to produce 2-keto-gulono-gamma-lactone that is spontaneously converted into ascorbic acid [8]. Since regucalcin is also an essential gene that is involved in calcium homeostasis, not surprisingly the loss of the ability to synthesize vitamin C is always associated with loss of the *GULO* gene.

**Fig. 3:**
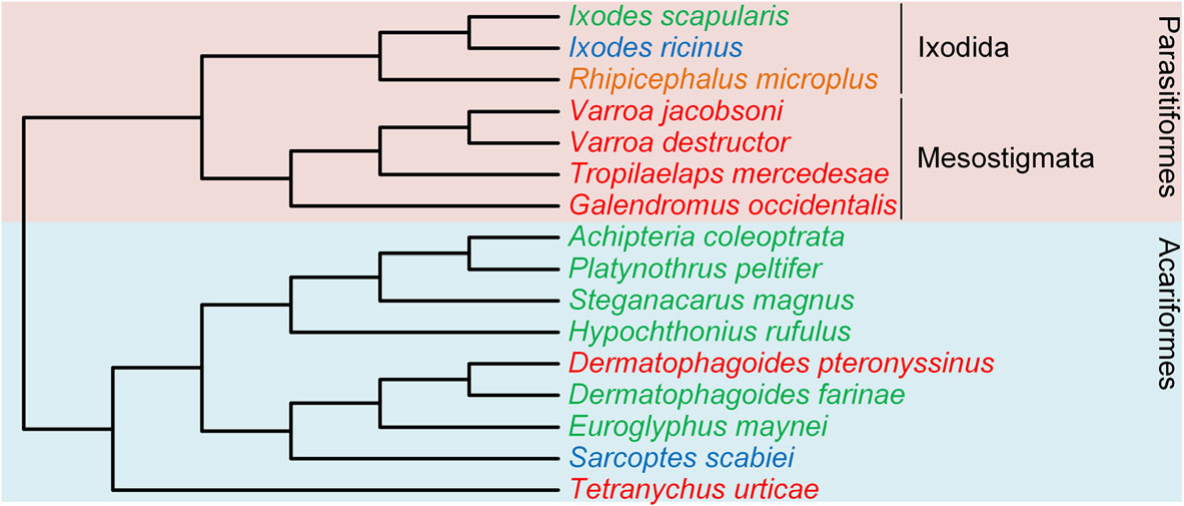
Cladogram representation of the findings regarding the annotation of *GULO* in 16 Acari species. The species in which *GULO* is likely present are highlighted in green, the species where the presence or absence of *GULO* cannot be inferred are highlighted in blue, the species in which the annotations may be the result of genome contamination are highlighted in orange and the species where *GULO* is probably absent are highlighted in red. The pink and light blue regions differentiate the Acari species analyzed into two superorders, respectively Parasitiformes and Acariformes. The Parasitiformes group can be seen divided in two distinct orders, Ixodida and Mesostigmata. The cladogram branches represent the taxonomic relationship between the species analyzed, depicted as in [30–34]

The Acari species analyzed are representative of two Acari superorders, namely Acariformes, and Parasitiformes. Within the Parasitiformes, we were able to annotate a putative *GULO* gene in two species belonging to the Ixodidae family included in the Ixodida order (*I. scapularis* and *R. microplus*), although with further phylogenetic analysis we uncovered that the *R. microplus* annotation obtained may be the result of genome contamination. Curiously, the *I. scapularis* annotation also shows irregular placement in the obtained phylogenies for this dataset, when observing the taxonomic context. This is probably due to the complexity of our dataset regarding the greatly divergent species analyzed rather than a problem with our annotation, and as such, we consider *GULO* to be present in this species. This consideration is supported by the results of Wheeler et al., where *I. scapularis* was presented as a species with an identifiable *GULO* gene, although no details are given by these authors [10]. Additionally, the *I. ricinus* species (also included in the Ixodida order) genome had features of a possible *GULO* gene presence, however the *tblastn* alignment was scattered across many genomic scaffolds. Therefore, we cannot confidently infer the presence or absence of *GULO* in *I. ricinus*, but given the results obtained for *I. scapularis*, it is possible to extrapolate that a *GULO* gene may be present in the Ixodida order, specifically in the Ixodinae subfamily in which these two species are included. We could not annotate a putative *GULO* gene in the four remaining Parasitiformes species (*G. occidentalis*, *V. destructor*, *V. jacobsoni* and *T. mercedesae*) belonging to the Mesostigmata order (Gamasina infraorder), due to the lack of sequence homology in the performed *tblastn* search. This result presents strong evidence regarding the possible loss of *GULO* in the Mesostigmata lineage. The remaining nine Acari species analyzed belong to the Acariformes, where a *GULO* gene seems to be present in most species (Fig. 3).

**Fig. 4:**
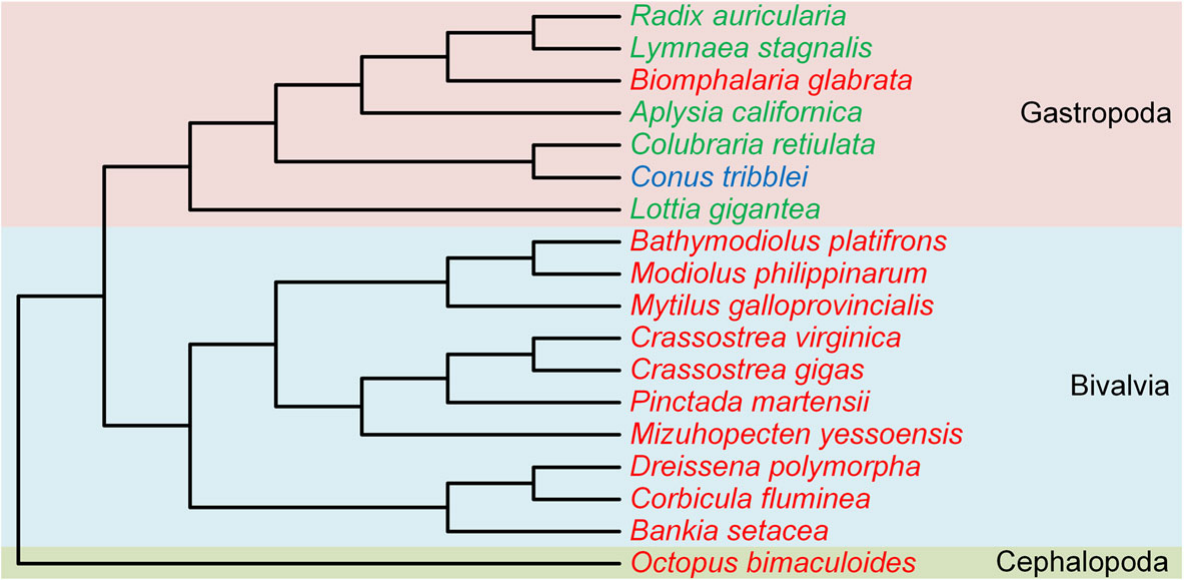
Cladogram representation of the findings regarding the annotation of *GULO* in 18 Mollusca species. The species in which *GULO* is likely present are highlighted in green, the species where the presence or absence of *GULO* cannot be inferred are highlighted in blue and the species where *GULO* is probably absent are highlighted in red. The pink, light blue and olive green regions differentiate the Mollusca species analyzed into three classes, respectively Gastropoda, Bivalvia and Cephalopoda. The cladogram branches represent the taxonomic relationship between the species analyzed, depicted as in [30, 35–38]

Although we show the presence of the *GULO* gene in the Gastropoda, within the Mollusca phylum, this gene is lost in the Bivalvia lineage and may or not be present in the Cephalopoda group. The results for the Molusca group are summarized in Fig. 4. It is likely that *GULO* was lost in the Bivalvia class but was present in the common ancestral of both Bivalvia and Gastropoda taxonomic groups, given that this gene is maintained in the majority of the gastropods analyzed. Inside the Gastropoda group, *GULO* may have been recently lost in *B. glabrata*, a member of the Planorboidea superfamily included in one of the Gastropoda subclasses, Heterobranchia, since it appears present in two closely related species, *L. stagnalis* and *R. auricularia*. Regarding the Cephalopoda group, we cannot infer the presence or absence of *GULO* since only one representative genome was available for inquiry, belonging to *O. bimaculoides*. Nevertheless, *GULO* seems to not be present at least in this species.

**Fig. 5:**
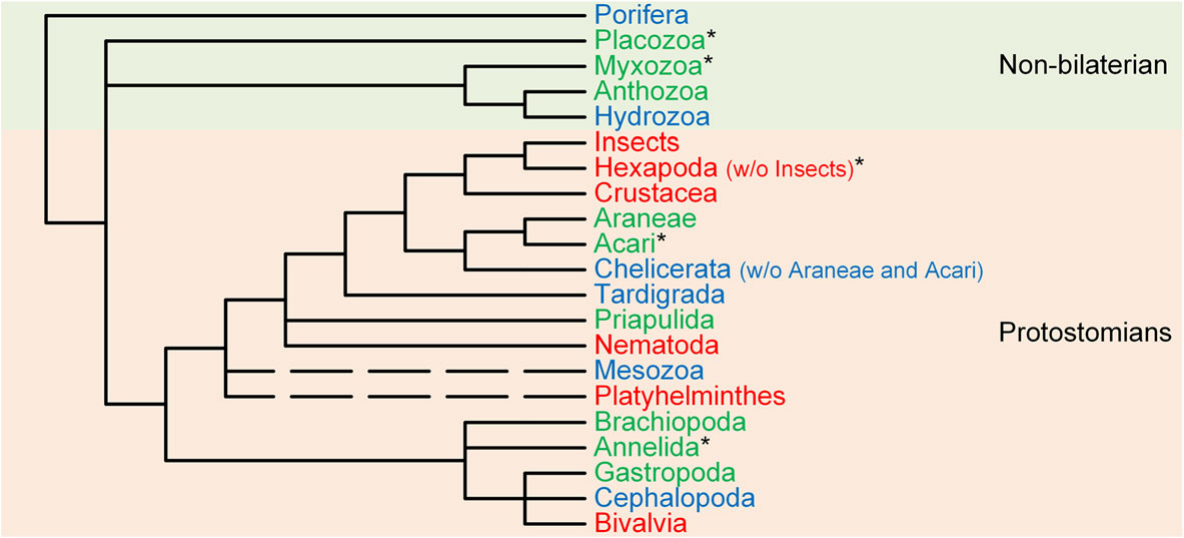
Summary of the main findings regarding the presence of putative functional *GULO* genes among non-Bilaterian and Protostomia species across the different phyla. Data from the analysis of *GULO* orthologs found in species with non-annotated genomes, as also data from a previous analysis of *GULO* orthologs identified amongst species with annotated genomes, were both considered to construct this diagram [17]. The colour code represents lineages where: a putative functional *GULO* ortholog was detected (green); no *GULO* ortholog was detected (red); or lineages for which, due to the limited amount available genomes, no conclusion can be withdrawn (blue). Asterisks highlight the phyla for which new conclusions were withdrawn after *GULO* orthologs identified in species with non-annotated genomes were included in the analysis, when compared to the data shown in [17]. Broken lines show uncertain relationships. Taxonomic relationships are depicted as in [30]

Similarly to *I. scapularis*, it should be noted that the *B. belcheri, N. vectensis, I. scapularis*, *T. adhaerens* and *P. caudatus* species are misplaced within the animal branch of the Bayesian consensus tree (Fig. 2), but given that we are analysing species that have been diverging for more than 650 million years [39], such misplacements are not that uncommon*.* For instance, they could be due to the lack of synapomorphy due to multiple nucleotide substitutions or stochastic errors that lead to erratic phylogenies, or random nucleotide substitutions that create sequence similarities that result in the clustering of distantly related lineages, or even due to different species-specific evolutionary rates [39, 40]. Figure 5 shows a summary of our findings regarding *GULO* presence/absence in the different non-Bilateria and Protostomia animal lineages. It should be noted that in order to state that a *GULO* gene is missing in a given lineage we require that no *GULO* ortholog, with all expected features, is detected in at least three species from that lineage. In conclusion, a *GULO* gene is found in the non-Bilateria Placozoa, Myxozoa and Anthozoa classes, and in the Protostomian Araneae family, the Gastropoda class, the Acari subclass, and the Priapulida, Annelida and Brachiopoda phyla lineages (Fig. 5). Nevertheless, it is not present in the Pancrustacea (Hexapoda and Crustacea), Nematoda, Platyhelminthes, and Bivalvia. Therefore, the *GULO* gene has been lost multiple times independently within Protostomia.

### *D. melanogaster* synthesizes vitamin C

**Fig. 6:**
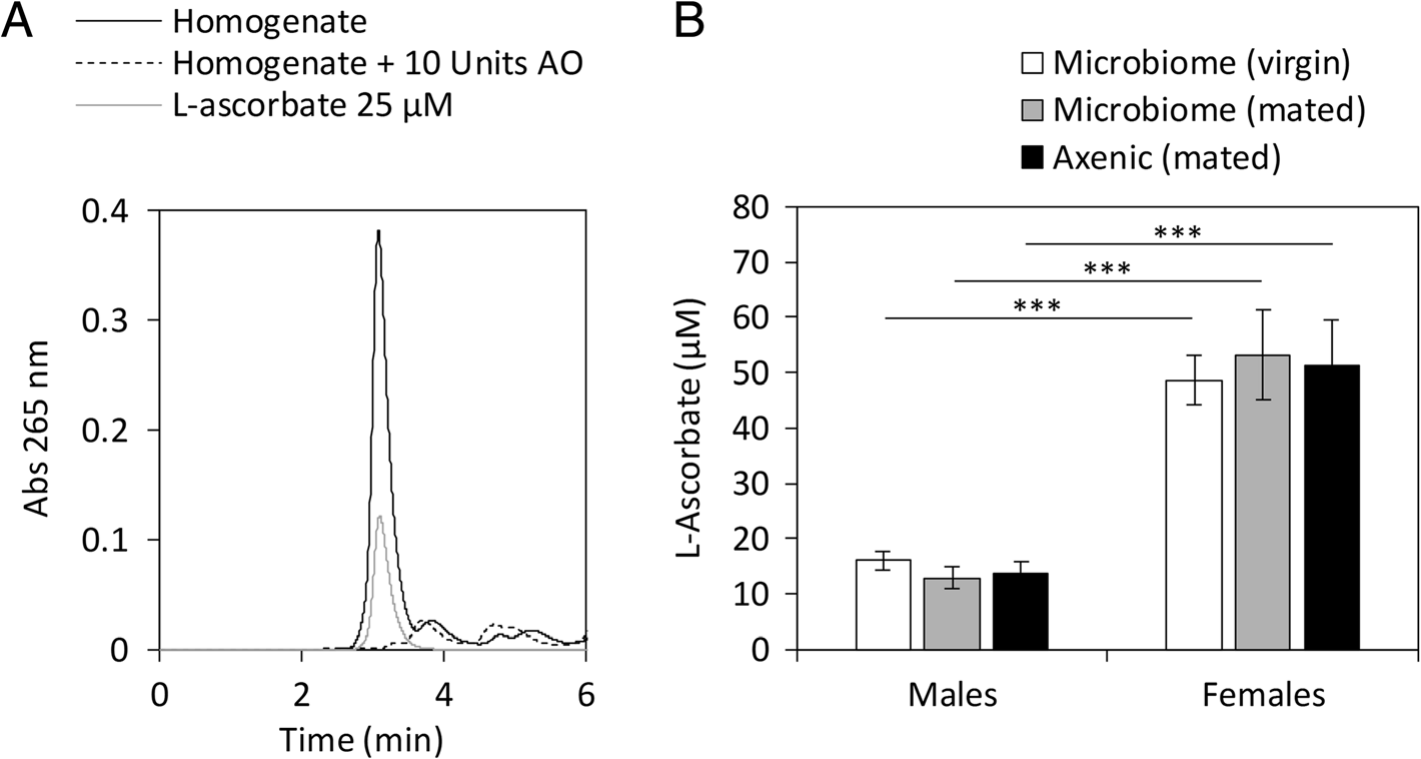
*D. melanogaster* females shown L-ascorbate levels that are three times higher than those measured in male flies. A) L-ascorbate levels were assessed in homogenates containing 25 individuals per millilitre of extraction buffer using reverse-phase HPLC, as described in “Material and Methods” (black line). To guaranty the identity of the L-ascorbate peak, 10 units of Ascorbate Oxidase (AO) were used to oxidise the L-ascorbate present in the sample (dashed line). A chromatogram resulting from the separation of a 10 μM L-ascorbate standard, prepared in extraction buffer, is also shown (grey line). B) The levels of L-ascorbate were assessed in virgin (white bars), mated (grey bars) and in axenic mated flies (black bars) from both genders. The values represent the averages of at least three independent experiments, and the error bar represents the standard deviation. A two-tailed unpaired t-test was used to identify significant differences between samples; *** *P*-value ≤0.001

Although L-ascorbate is biologically important in insects (see “Background”), there is a general consensus that invertebrates obtain L-ascorbate from their diet rather than synthetizing it, which agrees with the lack of a *GULO* ortholog in this taxonomic group [16–18]. Nevertheless, recently, it has been shown that the invertebrate *C. elegans* is able to synthesize L-ascorbate, likely by a yet to be identified pathway [21]. Therefore, we started exploring the hypothesis that insects may also synthesize L-ascorbate. The Oregon-R flies here used have been maintained in the lab for many generations and the food we use to rear them does not contain any source of Vitamin C. This was confirmed by attempting to detect Vitamin C in both fresh and male-inhabited 7-days old food using the same HPLC methodology used and sample amount (20 mg) as for the fly samples (see below; Additional file 3 Figure S2). Given this observation, we reasoned that, if *D. melanogaster* gets all Vitamin C from the diet, we should not be able to detect L-ascorbate in our Oregon-R flies. Nevertheless, this is not the case (Fig. 6A and B). Significant Vitamin C levels are observed both in males and females, although there is a striking difference between them (males: 16.0 ± 1.8 μM; females: 48.6 ± 4.4 μM). The body weight (wet weight) of female flies is approximately 50% higher than male bodies and thus, cannot alone explain the 3-fold higher L-ascorbate levels measured in female bodies. It should be noted that, in order to ensure the identity of the peak corresponding to L-ascorbate, we have treated a sample with 10 units of ascorbate oxidase, which led to the extinction of the L-ascorbate peak (Fig. 6A). Moreover, the levels of L-ascorbate we have determined in 7 days old Oregon R male flies (0.0902 +/− 0.015 μg/fly) are within the same range of those determined in [16] in flies with a similar age raised under similar conditions and which ranged approximately from 0.04 to 0.10 μg/fly. It is difficult to compare Vitamin C levels in small animals such as *Drosophila* flies and large invertebrate and vertebrates because our measurements are for whole flies, while measurements for larger animals are usually given for specific tissues, but is similar to that reported for the mullet brain, the rat liver, or the guinea pig lung and brain, for instance (see [14]).

Given the above results, we have raised the hypothesis that the fly’s microbiome may be responsible, or at least contributing to, the synthesis of L-ascorbate and we repeated the analysis using axenic flies. Since these flies must be kept under sterile conditions until collection, male and female flies could not be separated after hatch. Therefore, to ensure that the mating state was not affecting the L-ascorbate levels observed, we have also measured this vitamin in control conditions using 7-days-old male and female flies that were kept together during that time-frame. Although there are differences between the genders, no significant differences are observed between the L-ascorbate levels measured in mated, virgin or axenic individuals from the same gender (Fig. 6B), suggesting that the microbiome does not influence or contributes for the synthesis of this vitamin.

We also expanded the *D. melanogaster* microbiome ex-vivo, by inoculating MRS liquid medium with a homogenate obtained from 25 whole individuals, previously washed with sodium hypochlorite solution (2.7% v/v) to remove any environmental microbial contaminants (see “Methods”). The MRS media was chosen given that it allows the propagation of several bacteria genera that have been implicated in L-ascorbate synthesis, including *Gluconobacter, Gluconacetobacter and Acetobacter* [41–43]. No L-ascorbate was found in the supernatant or in the pellet of bacterial cells. Therefore, the microbiome is not the source of the observed L-ascorbate.

**Fig. 7:**
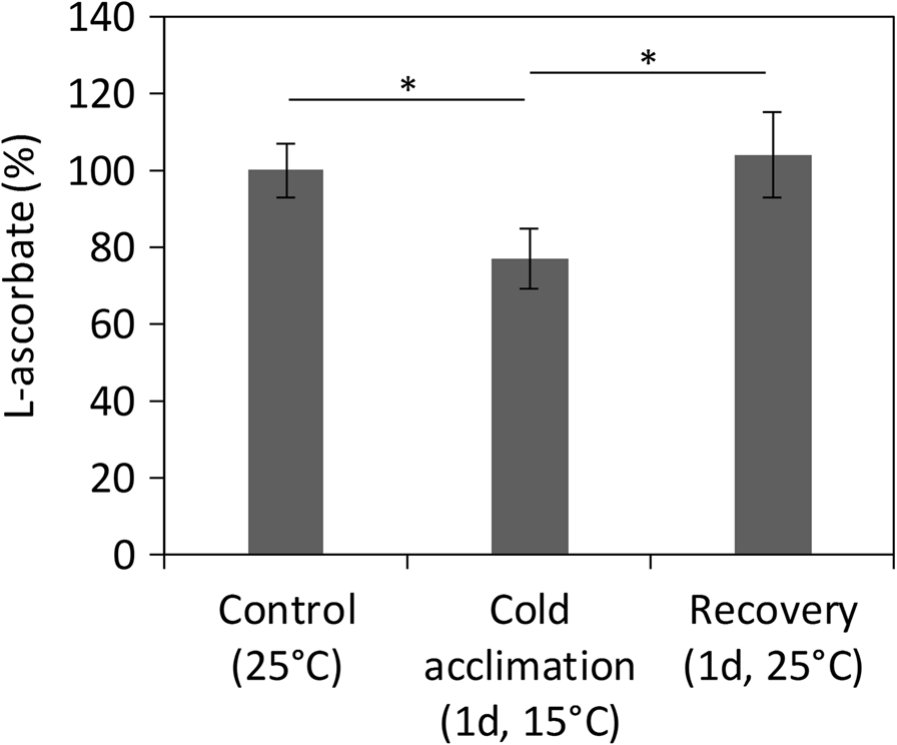
*D. melanogaster* L-ascorbate levels decrease during cold acclimation (15 °C) and are fully recovered after 1 d at 25 °C. L-ascorbate was measured in 7-days-old flies (control), in flies incubated for one day at 15 °C (cold acclimation) and in flies subjected to cold acclimation followed by a recovery at 25 °C during one day (recovery). L-ascorbate levels decrease approximately 23% after 1 d at 15 °C and are recovered to the levels measured in the control after 1 d of recovery at 25 °C. The indicated averages were obtained from, at least, three independent experiments, and the error bar represent the associated standard deviation. A two-tailed unpaired t-test was used to identify significant differences between samples; * P-value ≤0.05

Given that a previous study by Massie et al. has shown that a brief cold exposition (10 min at 4 °C) results in an increase of the L-ascorbate levels in Oregon-R male flies [16], we tested whether cold acclimation could also trigger a similar response using our strain, which would reinforce the idea that L-ascorbate is synthetized by the fly. We have assessed L-ascorbate levels in flies raised in standard conditions, in flies subjected to cold acclimation (one day at 15 °C), and in flies that were allowed to recover at 25 °C for one day (Labelled as “Control”, “Cold acclimation”, and “Recovery”, respectively, in Fig. 7). The flies used in these experiments were 7-days-old male flies. We observed a 23% decrease in the L-ascorbate levels after one day of cold acclimation, which was recovered back to the levels measured in control flies, after recovery (Fig. 7). Therefore, in contrast to the results reported in [16], obtained after a very short cold exposure, flies exposed to the usual cold-acclimation protocol have lowered levels of Vitamin C, possibly because it is being used as an anti-oxidant to protect the cell from oxidative damage.

In conclusion, the above experiments strongly suggest that, as reported for *C. elegans*, *D. melanogaster* synthetizes Vitamin C using a yet to be identified pathway. Therefore, in Protostomians the lack of a *GULO* gene cannot be equated with inability to synthesize L-ascorbate.

## Discussion

The phylogenetic analyses here performed clearly show that the *GULO* gene is present in the non-Bilateria Placozoa, Myxozoa and Anthozoa groups, and in the Protostomian Araneae family, the Gastropoda class, the Acari subclass, and the Priapulida, Annelida and Brachiopoda phyla lineages. In animals, *GULO* always encodes a protein with a HWAK motif, that is also present in the fungi sequences here used as references. *GULO* seems to be thus, a very ancient gene. How far back in time *GULO* orthologs can be found remains to be determined. This gene was lost independently multiple times, both within the Protostomia (in the Pancrustacea, Nematoda, Platyhelminthes and Bivalvia), as here shown, as well as within the Deuterostomia.

In Protostomia, the absence of a *GULO* gene does not, however, imply that a given species is unable to synthesize Vitamin C. Although no *GULO* gene was identified in 35 Nematode genomes [17], *C. elegans* has been shown to be able to synthesize L-ascorbate [21]. Here we show that: i) *D. melanogaster* flies raised for many generations in Vitamin C free food still show high L-ascorbate levels; ii) Vitamin C levels are gender-specific; iii) axenic flies have similar Vitamin C levels to mated and virgin control flies; iv) when expanded ex vivo no L-ascorbate is detected in the fly microbiome; and v) when exposed to cold acclimation conditions, L-ascorbate levels drop but then return to normal levels when flies are brought back to standard conditions. These results can only be explained if *D. melanogaster* flies synthesize Vitamin C, likely via an alternative pathway, or using another enzyme that is able to catalyse the same reaction as GULO. The aforementioned results, also illustrate the importance of the synthesis and maintenance of L-ascorbate levels for the *D. melanogaster* capacity to respond to cold stresses, and likely to other abiotic stresses, and emphasises the importance of elucidating the pathway by which this model organism produces this vitamin. Such knowledge could then be extended to other, less genetically-accessible, insect species where L-ascorbate is also playing, or adding, important functions in the cell [16, 18–20].

The *GULO* gene always encodes a protein with a HWAK motif, and this property can be used to quickly and easily identify the *GULO* gene using Blast PHI searches. It has been shown that individual mutations in the histidine, tryptophan and lysine residues of HWXK motif lead to complete enzymatic activity loss, largely related with the ineptitude of the affected proteins to bind to their cofactor [29]. The impact of changing the alanine at the HWAK motif was not yet addressed in vivo, but our protein structure predictions suggest that changing the alanine at the HWAK motif by a glycine has a profound impact on protein structure. Moreover, mammal GULO and fungi ALO proteins, which contain a HWAK motif, establish a covalent bond with their FAD cofactor, while bacterial L-gulonolactone dehydrogenase (GULDH) and the protozoans *Trypanosoma cruzi* and *Trypanosoma brucei* ALO, which contain a HWGK motif, are thought to bind non-covalently to a flavin cofactor and FMN, respectively [22, 44–47]. Here we find that the HWAK motif region and the GULO FAD-binding domain are located close to each other in the predicted structure of the *M. musculus* GULO protein, and this may be important for establishing a covalent bond with the FAD cofactor. Given these observations, it is possible that the presence of an alanine at the HWAK motif confers a stronger and permanent covalent bond between GULO and the correspondent cofactor due to distinct protein configuration relative to the HWGK alternative, in which the protein/cofactor interaction may be much weaker. This, in turn can be related to distinct catalytic properties between the two proteins.

## Conclusion

Non-Bilaterian and Protostomians synthesize Vitamin C either through the conventional animal pathway or an alternative pathway, but in this animal group, not being able to synthesize L-ascorbate seems to be the exception rather than the rule.

## Methods

### Identification of *GULO* orthologs in non-Bilaterian and Protostomian species with non-annotated genomes

Using the non-Bilaterian and Protostomian taxonomic group names as queries, we downloaded 35 non-annotated species genomes from NCBI (https://www.ncbi.nlm.nih.gov/assembly/; four non-Bilaterian genomes and 31 Protostomian genomes excluding Insects, Nematoda and Platyhelminthes; see Results). In addition, we re-analysed 29 non-Bilateria and Protostomian species genomes analysed in [17], and were able to obtain putative *GULO* annotations for some species where such gene has not been annotated, or where the gene shows an unexpected length. Only genomes from the GenBank database were downloaded, since many of the species of interest did not have a representative genome in the RefSeq database. The SEDA software (http://www.sing-group.org/seda/) was used to identify and annotate putative *GULO* orthologs. Since, when performing a *tblastn* (BLAST algorithm version: 2.7.1+) with SEDA, it is possible to retrieve the high-scoring segment pairs (HSPs) as well as the flanking regions, in our case, 5000 nucleotides on both sides of the HSPs, we first performed this operation using the GULO protein sequence from *Mus musculus* as a query (NP_848862.1), an expect value of 0.05, and the genomes of interest as subject, to obtain the genome regions of interest. Nucleotide sequences were further processed using the *“Grow sequences”* operation included in the SEDA software, with a selected minimum overlap of 2500 nucleotides. This step allows the growing of overlapping sequences, simplifying the annotation process. The sequences were then annotated by hand (under the assumption that intron splice sites follow the canonical GT-AG rule), using the results of *tblastn* searches as a guide (the *M. musculus* (NP_848862.1) GULO protein was used as query and the word size was set to two). Moreover, if none of the putative exons encoded a protein with the typical HWXK amino acid pattern [29], no annotation was attempted. Several non-Bilateria and Protostomian *GULO* representative sequences identified in [17] were added to this dataset, as well as a set of *GULO* sequences to be used as references, namely *M. musculus* (NP_848862.1), *Xenopus laevis* (OCT81467.1), *Priapulus caudatus* (XP_014666894.1), *Gallus gallus* (XP_015140704.1), *Alligator mississippiensis* (KYO43973.1), *Lepisosteus oculatus* (XP_015207781.1), *Branchiostoma belcheri* (XP_019645195.1), *Nematostella vectensis* (EDO44935.1), *Saccharomyces cerevisiae S288C* (NP_013624.1) and *Metarhizium majus ARSEF 297* (XP_014580409.1). The ADOPS pipeline was used to perform the alignment and the phylogenetic analyses [29]. The MUSCLE alignment algorithm was used as implemented in T-Coffee [49]. Sequences were first aligned at the amino acid level and the corresponding nucleotide alignment obtained. Only codons with a support value above two were used for the Bayesian (MrBayes [50]) phylogenetic analyses. The General Time-Reversible (GTR) model of sequence evolution was used, allowing for among-site rate variation and a proportion of invariable sites. Third codon positions were allowed to have a gamma distribution shape parameter different from that of first and second codon positions. Two independent runs of 5000,000 generations with four chains each (one cold and three heated chains) were performed. Convergence was achieved, since the potential scale reduction factor for all parameters was around 1.00. Trees were sampled at every 100th generation with a defined burn-in of 25% for the complete analysis (the first 12,500 samples were discarded). The resulting Bayesian trees, in Nexus format, were converted to Newick format using the Format Conversion Website (http://phylogeny.lirmm.fr/phylo_cgi/data_converter.cgi), and imported to MEGA X (https://www.megasoftware.net/) to root the consensus tree [51].

### Inferred GULO protein structures

Protein structure predictions were obtained using the I-TASSER server (https://zhanglab.ccmb.med.umich.edu/I-TASSER/). The model with the highest C-score was always chosen as being the most likely protein structure. The C-score is usually between − 5 and 2, and values close to 2 indicate a model with higher confidence. For the *M. musculus* GULO (NP_848862.1), we obtained two models with C-score values of 0.22 and 0.68. The change of an alanine by a Glycine at the HWXK motif in this sequence results in three alternative prediction models with C-scores of − 0.65, − 0.41 and 0.00.

### *D. melanogaster* husbandry

Oregon-R flies, obtained from the *Drosophila* Stock Centre (http://blogs.cornell.edu/*Drosophila*/), were reared at 25 °C, with a relative humidity of 70%, under a 12 h light-dark cycle. Flies were fed with yeast-based medium (YBM) containing 40 g.l^− 1^ wheat flour, 80 g.l^− 1^ sugar, 10 g.l^− 1^ agar, 100 g.l^− 1^ yeast extract, 4 g.l^− 1^ NaCl, 5 ml.l^− 1^ propionic acid and 45 g.l^− 1^ Methyl 4-hydroxybenzoate. Unless stated otherwise, flies were always collected under brief CO_2_ anaesthesia, and all experimental groups were matched for age.

### Generation of axenic *D. melanogaster*

To generate axenic flies, Oregon-R flies were placed in a cage, where a plate containing a wet paste of yeast extract was introduced, and where the flies were allowed to lay eggs during 2 h. The yeast and eggs mixture was then resuspended in water and the eggs collected using a sieve and transferred to an Eppendorf tube. The eggs were pelleted by centrifugation at 350 g and washed twice with a sodium hypochlorite solution (2.7% v/v) for 2.5 min, to remove the chorion and thus all microbial cargo (as described in [41]). From this moment on all steps were performed under sterile conditions. The dechorinated eggs were washed three times using a sterile saline triton solution (300 μl.l^− 1^ Triton X-100, 4 g.l^− 1^ NaCl) and then placed in UV-sterilized food containers. To test whether the microbiome was effectively removed in axenic flies, three axenic flies and three individuals raised under routine conditions (controls), were collected, washed with sodium hypochlorite solution (2.7% v/v), and homogenized in 200 μl of a sterile 0.9% (w/v) NaCl solution. One hundred microliters of the homogenate were spread in Luria-Bertoli (LB) solid medium and the plates incubated at 25 °C for 2 days. Bacterial growth was observed in the control plates, but not in the plates inoculated with homogenates from axenic flies.

### Preparation of crude homogenates from *D. melanogaster*

For each tested condition, crude extracts were prepared using an extraction buffer containing 0.1 mM diethylenetriaminepentaacetic acid and 0.01 M HEPES buffer, pH 7.2 (both from Sigma-Aldrich) based on the method described by [16]. Briefly, 25 individuals were homogenized using 500 μl of extraction buffer in a Dounce homogenizer, which was rinsed with another 500 μl of extraction buffer. The resulting 1 ml of crude extract was then centrifuged at 9000 g for 5 min, and 850 μl of the supernatant were collected and centrifuged again at 20,000 g for 20 min. The resulting supernatant was divided into three individual technical replicates (250 μl each), which were centrifuged for another 15 min at 20,000 g and the clear supernatants were immediately used to measure L-ascorbate concentration using High Pressure Liquid Chromatography (HPLC). All centrifugations were performed at 4 °C, and all steps performed in ice.

## Assessment of L-ascorbate concentration using HPLC

L-ascorbate was detected using HPLC, based on the method described by [21]. Briefly, 90 μl of clear extracts were injected into a LiChrospher® 100 RP-18 (5 μm) LiChroCART® 250–4 reversed-phase column (Merck) and separated at a flow rate of 1 ml.min^− 1^ (25 °C). Two buffers were used on the chromatographic analysis, buffer A consisting of 20 mM triethylammonium acetate (Sigma-Aldrich), pH 6.0, and buffer B containing 20 mM triethylammonium acetate in 40% acetonitrile (Merck), pH 6.0. The gradients used to elute the samples were exactly those described in [21] and the L-ascorbate signal was detected at 265 nm. To estimate L-ascorbate concentration in the crude homogenates, L-ascorbate (Sigma-Aldrich) standards of 25, 50 and 100 μM where prepared using extraction buffer as solvent, to obtain a calibration curve. To guaranty the identity of the L-ascorbate peak, 10 Units of ascorbate oxidase (Sigma-Aldrich) were added to 200 μl of crude extract and the mixture incubated for 20 min at 25 °C prior to injection into the HPLC apparatus.

### Determination of L-ascorbate in microbiome cultures expanded ex vivo

To test if the microbiome of flies contributes to the production of L-ascorbate we have measured the amount of L-ascorbate produced by microbiome cultures that were expanded ex vivo in De Man, Rogosa and Sharpe (MRS) media. For that, 25 flies were anesthetized with CO_2_ and washed with sodium hypochlorite solution (2.7% v/v) followed by three washes with sterile water. Then, the flies were homogenized in a total volume of 1 ml of 0.9% (w/v) NaCl solution and this suspension was used to inoculate 100 ml of MRS medium supplemented, or not, with 2% w/v glucose. The inoculated flasks were incubated at 25 and 30 °C, and the microbial growth was followed by measuring the absorbance at 600 nm. Samples were collected 1, 2 and 3 days after inoculation, and L-ascorbate levels were determined in the supernatant and in the pellet of bacterial cells. To determine L-ascorbate in the supernatant, 900 μl of the clear medium were combined with 100 μl of 10x extraction buffer prior to injection in the HPLC. To determine L-ascorbate in the pellet, cells were resuspended in 1 ml of 1% SDS (Sodium dodecyl sulfate), 0.2 M NaCl solution and vortexed for 1 min. The homogenate was centrifuged at 18,000 g for 5 min (4 °C) and 800 μl of the supernatant were combined with 200 μl of 10x extraction buffer prior to injection into the HPLC.

### Cold acclimation experiments

Seven-days-old male flies that were separated from females 8 h after hatching and kept for 7 days under control conditions were used. Cold acclimation stress was induced by transferring the flies (in vials with food) to a 15 °C chamber and kept at this temperature for one day (condition labeled as “cold acclimation, 1d, 15 °C”; see Results). Then, for recovery, the containers were transferred to 25 °C and kept at this temperature for another day. Each condition was tested in triplicate, and 25 flies were collected per condition for HPLC analysis. All samples were snap-frozen in liquid nitrogen and kept at − 80 °C.

## Additional files

Additional file 1: **Table S1.** Summary of the annotation process on the downloaded non-Bilaterian and Protostomian genomes. The rows display information on the analysed species taxonomic groups and names, while the columns present information on the annotations steps performed on the species genomes and the obtained final results. The annotation steps were performed in succession from left to right, and show respectively if any sequences were retrieved from the initial tblastn, if the obtained sequences had a conserved HWXK motif, if the obtained alignment was residual or considerable, if the sequences had exons spread across several scaffolds, if the final obtained coding sequence had stop codons, and the final CDS alignment coverage. (XLSX 11 kb)

Additional file 2: **Figure S1.** Phylogenetic relationship amongst the putative *GULO* orthologs identified amongst all Protostomian and non-Bilaterian species with annotated and non-annotated genomes. Two Fungi *GULO* CDS were used to assist the rooting the consensus tree (red pane). *GULO* CDS of six deuterostomian species, representing the Actinopteri, Amphibia, Euarchontoglires, Aves, Reptilia and Cephalochordata groups, were used to facilitate the identification of functional *GULO* orthologs amongst Animal species (green pane). *GULO* CDS grouped outside the fungi group and that do not have the HWAK motif were removed to obtain Fig. 2. Higher taxonomic classifications for common species are shown on the right. (TIF 120810 kb)

Additional file 3: **Figure S2.** Chromatograms obtained from the analyses of three replicates of fresh (A1-A3) and male-inhabited 7-days old (B1-B3) food. L-ascorbate levels were assessed in homogenates containing 20 mg of food per millilitre of extraction buffer using reverse-phase HPLC, as described in “Material and Methods”. For each condition, individual food samples are represented with a suffix number (1–3). The chromatograms depict three lines representative of the processed technical replicas for each sample (blue, orange and green, respectively), and one indicative of a 25 μM L-ascorbate standard (black). All samples analysed show an absence of L-ascorbate. (TIF 53095 kb)

Additional file 4: Nucleotide alignment used in the phylogenetic analyses presented in Additional file 2 Figure S1. (PDF 244 kb)

Additional file 5: Nucleotide alignment used in the phylogenetic analyses presented in Fig. 2. (PDF 188 kb)

## Data Availability

The sequence data analysed during this study is available in Additional files 4 and 5. Raw HPLC chromatograms are reported in Fig. 6 as well as in Additional file 3 Figure S2.

## Abbreviations

ADOPS: Automatic Detection Of Positively Selected Sites
ALO: D-arabino-1,4-lactone oxidase
AO: Ascorbate Oxidase
FAD: Flavin adenine dinucleotide
FMN: Flavin mononucleotide
GLDH: L-Galactono-lactone dehydrogenase
GTR: General time-reversible model
GULDH: L-Gulonolactone dehydrogenase
GULO: L-Gulonolactone oxidase
HEPES: Hydroxyethyl piperazineethanesulfonic acid
HPLC: High-Performance Liquid Chromatography
HSPs: High-scoring segment pairs
JMJD3: Jumonji Domain-Containing Protein 3
LB: Luria-Bertoli
MRS: De Man, Rogosa and Sharpe
NCBI: National Center for Biotechnology Information database
PHI: Pattern-hit initiated
POX: Peroxidase
ROS: Reactive Oxigen species
SDS: Sodium dodecyl sulfate
SEDA: SEquence DAtaset builder
SMP30: Senescence Marker Protein 30
SOD: Superoxide dismutase
TET1: Ten-eleven translocation methylcytosine dioxygenase 1
VAO: Vanillyl-alcohol oxidase
YBM: Yeast-based medium

## References

[1] Figueroa-Méndez R, Rivas-Arancibia S (2015). Vitamin C in health and disease: its role in the metabolism of cells and redox state in the brain. Front Psychol.

[2] Podmore ID, Griffiths HR, Herbert KE, Mistry N, Mistry P, Lunec J (1998). Vitamin C exhibits pro-oxidant properties. Nature..

[3] Bahadorani S, Bahadorani P, Phillips JP, Hilliker AJ (2008). The effects of vitamin supplementation on *Drosophila* life span under normoxia and under oxidative stress. Journals Gerontol - Ser A Biol Sci Med Sci.

[4] Hansen SN, Tveden-Nyborg P, Lykkesfeldt J (2014). Does vitamin C deficiency affect cognitive development and function?. Nutrients..

[5] Kratzing CC, Kelly JD, Kratzing JE (1985). Ascorbic acid in fetal rat brain. J Neurochem.

[6] He XB, Kim M, Kim SY, Yi SH, Rhee YH, Kim T (2015). Vitamin C facilitates dopamine neuron differentiation in fetal midbrain through TET1- and JMJD3-dependent epigenetic control manner. Stem Cells.

[7] Qiu S, Li L, Weeber EJ, May JM (2007). Ascorbate transport by primary cultured neurons and its role in neuronal function and protection against excitotoxicity. J Neurosci Res.

[8] Drouin G, Godin J-R, Page B (2011). The genetics of vitamin C loss in vertebrates. Curr Genomics.

[9] Cui J, Yuan X, Wang L, Jones G, Zhang S (2011). Recent loss of vitamin C biosynthesis ability in bats. PLoS One.

[10] Wheeler G, Ishikawa T, Pornsaksit V, Smirnoff N (2015). Evolution of alternative biosynthetic pathways for vitamin C following plastid acquisition in photosynthetic eukaryotes. Elife..

[11] Tsurusaki Y, Yamaguchi M (2004). Role of regucalcin in liver nuclear function: binding of regucalcin to nuclear protein or DNA and modulation of tumor-related gene expression. Int J Mol Med.

[12] Yamaguchi M, Yamamoto T (1978). Purification of calcium binding substance from soluble fraction of Normal rat liver. Chem Pharm Bull (Tokyo).

[13] Carr RS, Neff JM (1980). Determination of ascorbic acid in tissues of marine animals by liquid chromatography with electrochemical detection. Anal Chem.

[14] Carr RS, Bally MB, Thomas P, Neff JM (1983). Comparison of methods for determination of ascorbic acid in animal tissues. Anal Chem.

[15] Dabrowski K, Hinterleitner S (1989). Applications of a simultaneous assay of ascorbic acid, dehydroascorbic acid and ascorbic sulphate in biological materials. Analyst..

[16] Massie HR, Shumway ME, Whitney SJP, Sternick SM, Aiello VR (1991). Ascorbic acid in *Drosophila* and changes during aging. Exp Gerontol.

[17] López-Fernández H, Duque P, Henriques SF, Vázquez N, Fdez-Riverola F, Vieira CP, Fdez-Riverola F, Mohamad M, Rocha M, De Paz J, González P (2018). A bioinformatics protocol for quickly creating large-scale phylogenetic trees. Practical applications of computational biology and bioinformatics, 12^th^ international conference. PACBB2018. Advances in intelligent systems and computing.

[18] Barbehenn RV, Bumgarner SL, Roosen EF, Martin MM (2001). Antioxidant defenses in caterpillars: role of the ascorbate-recycling system in the midgut lumen. J Insect Physiol.

[19] Kolawole AO, Olajuyigbe FM, Ajele JO, Adedire CO (2014). Activity of the antioxidant defense system in a typical bioinsecticide-and synthetic insecticide-treated cowpea storage beetle *Callosobrochus maculatus F.* (Coleoptera: Chrysomelidae). Int J Insect Sci.

[20] Zhang Y, Wu H, Xie J, Jiang R, Deng C, Pang H (2015). Transcriptome responses to heat and cold-stress in ladybirds (*Cryptolaemus montrouzieri Mulasnt*) analyzed by deep-sequencing. Biol Res.

[21] Patananan AN, Budenholzer LM, Pedraza ME, Torres ER, Adler LN, Clarke SG (2015). The invertebrate *Caenorhabditis elegans* biosynthesizes ascorbate. Arch Biochem Biophys.

[22] Wolucka BA, Communi D (2006). *Mycobacterium tuberculosis* possesses a functional enzyme for the synthesis of vitamin C, L-gulono-1,4-lactone dehydrogenase. FEBS J.

[23] Kado CI, Pujol CJ, Chan A, inventors. Avenir Genetics Llc, assignee. Microbiological method for producing ascorbic acid. International patent 040955. 2004-05-21.

[24] Chang YL, Rossetti M, Vlamakis H, Casero D, Sunga G, Harre N, et al. A screen of Crohn’s disease-associated microbial metabolites identifies ascorbate as a novel metabolic inhibitor of activated human T cells. Mucosal Immunol. 2018:1–11. 10.1038/s41385-018-0022-7 PMC6202286 29695840

[25] Arumugam M, Raes J, Pelletier E, Le Paslier D, Yamada T, Mende DR (2011). Enterotypes of the human gut microbiome. Nature..

[26] Sannino DR, Dobson AJ, Edwards K, Angert ER, Buchon N (2018). The *Drosophila melanogaster* gut microbiota provisions thiamine to its host. MBio..

[27] Blagojevic DP, Grubor-Lajsic GN, Spasic MB (2011). Cold defence responses: the role of oxidative stress. Front Biosci (Schol Ed).

[28] Aboobucker SI, Lorence A (2016). Recent progress on the characterization of aldonolactone oxidoreductases. Plant Physiol Biochem.

[29] Leferink NGH, Jose MDF, van den Berg WAM, van Berkel WJH (2009). Functional assignment of Glu386 and Arg388 in the active site of L-galactono-γ-lactone dehydrogenase. FEBS Lett.

[30] Maddison DR, Schulz KS, Maddison WP (2007). The tree of life web project. Zootaxa..

[31] Black WC, Klompen JSH, Keirans JE (1997). Phylogenetic relationships among tick subfamilies (Ixodida: Ixodidae: Argasidae) based on the 18S nuclear rDNA gene. Mol Phylogenet Evol.

[32] Liana M, Witaliński W (2005). Sperm structure and phylogeny of astigmata. J Morphol.

[33] Domes K, Althammer M, Norton RA, Scheu S, Maraun M (2007). The phylogenetic relationship between Astigmata and Oribatida (Acari) as indicated by molecular markers. Exp Appl Acarol.

[34] Dowling APG, Oconnor BM (2010). Phylogenetic relationships within the suborder Dermanyssina (Acari: Parasitiformes) and a test of dermanyssoid monophyly. Int J Acarol.

[35] Taylor JD, Williams ST, Glover EA, Dyal P (2007). A molecular phylogeny of heterodont bivalves (Mollusca: Bivalvia: Heterodonta): new analyses of 18S and 28S rRNA genes. Zool Scr.

[36] Plazzi F, Ceregato A, Taviani M, Passamonti M (2011). A molecular phylogeny of bivalve mollusks: ancient radiations and divergences as revealed by mitochondrial genes. PLoS One.

[37] Zapata F, Wilson NG, Howison M, Andrade SCS, Jörger KM, Schrödl M (2014). Phylogenomic analyses of deep gastropod relationships reject Orthogastropoda. Proc R Soc B Biol Sci.

[38] Liu J, Liu H, Zhang H (2018). Phylogeny and evolutionary radiation of the marine mussels (Bivalvia: Mytilidae) based on mitochondrial and nuclear genes. Mol Phylogenet Evol.

[39] Peterson KJ, Lyons JB, Nowak KS, Takacs CM, Wargo MJ, McPeek MA (2004). Estimating metazoan divergence times with a molecular clock. Proc Natl Acad Sci U S A.

[40] Wägele JW, Mayer C (2007). Visualizing differences in phylogenetic information content of alignments and distinction of three classes of long-branch effects. BMC Evol Biol.

[41] Simhadri RK, Fast EM, Guo R, Schultz MJ, Vaisman N, Ortiz L (2017). The gut commensal microbiome of *Drosophila melanogaster* is modified by the endosymbiont *Wolbachia*. mSphere..

[42] Bremus C, Herrmann U, Bringer-Meyer S, Sahm H (2006). The use of microorganisms in l-ascorbic acid production. J Biotechnol.

[43] Newell PD, Chaston JM, Wang Y, Winans NJ, Sannino DR, Wong ACN (2014). In vivo function and comparative genomic analyses of the drosophila gut microbiota identify candidate symbiosis factors. Front Microbiol.

[44] Sugisawa T, Ojima S, Matzinger PK, Hoshino T (1995). Isolation and characterization of a new vitamin C producing enzyme (L-Gulono-γ-lactone dehydrogenase) of bacterial origin. Biosci Biotechnol Biochem.

[45] Smirnoff N (2011). Vitamin C: the metabolism and functions of ascorbic acid in plants. Adv Bot Res.

[46] Logan FJ, Taylor MC, Wilkinson SR, Kaur H, Kelly JM (2007). The terminal step in vitamin C biosynthesis in Trypanosoma cruzi is mediated by a FMN-dependent galactonolactone oxidase. Biochem J.

[47] Wilkinson SR, Prathalingam SR, Taylor MC, Horn D, Kelly JM (2005). Vitamin C biosynthesis in trypanosomes: a role for the glycosome. Proc Natl Acad Sci U S A.

[48] Reboiro-Jato D, Reboiro-Jato M, Fdez-Riverola F, Fonseca NA, Vieira J, Rocha M, Luscombe N, Fdez-Riverola F, Rodríguez J (2012). On the development of a pipeline for the automatic detection of positively selected sites. 6^th^ international conference on practical applications of Computational Biology & Bioinformatics. Advances in intelligent and soft computing.

[49] Notredame C, Higgins DG, Heringa J (2000). T-coffee: a novel method for fast and accurate multiple sequence alignment. J Mol Biol.

[50] Ronquist F, Teslenko M, Van Der Mark P, Ayres DL, Darling A, Höhna S (2012). Mrbayes 3.2: efficient bayesian phylogenetic inference and model choice across a large model space. Syst Biol.

[51] Kumar S, Stecher G, Li M, Knyaz C, Tamura K (2018). MEGA X: molecular evolutionary genetics analysis across computing platforms. Mol Biol Evol.

