# Supplementary figures and images for: Multiple independent L-gulonolactone oxidase (GULO) gene losses and vitamin C synthesis reacquisition events in non-Deuterostomian animal species
Source: BMC Evol Biol. 2019 Jun 18;19:126. doi: 10.1186/s12862-019-1454-8 (PMC6582580; doi:10.1186/s12862-019-1454-8)

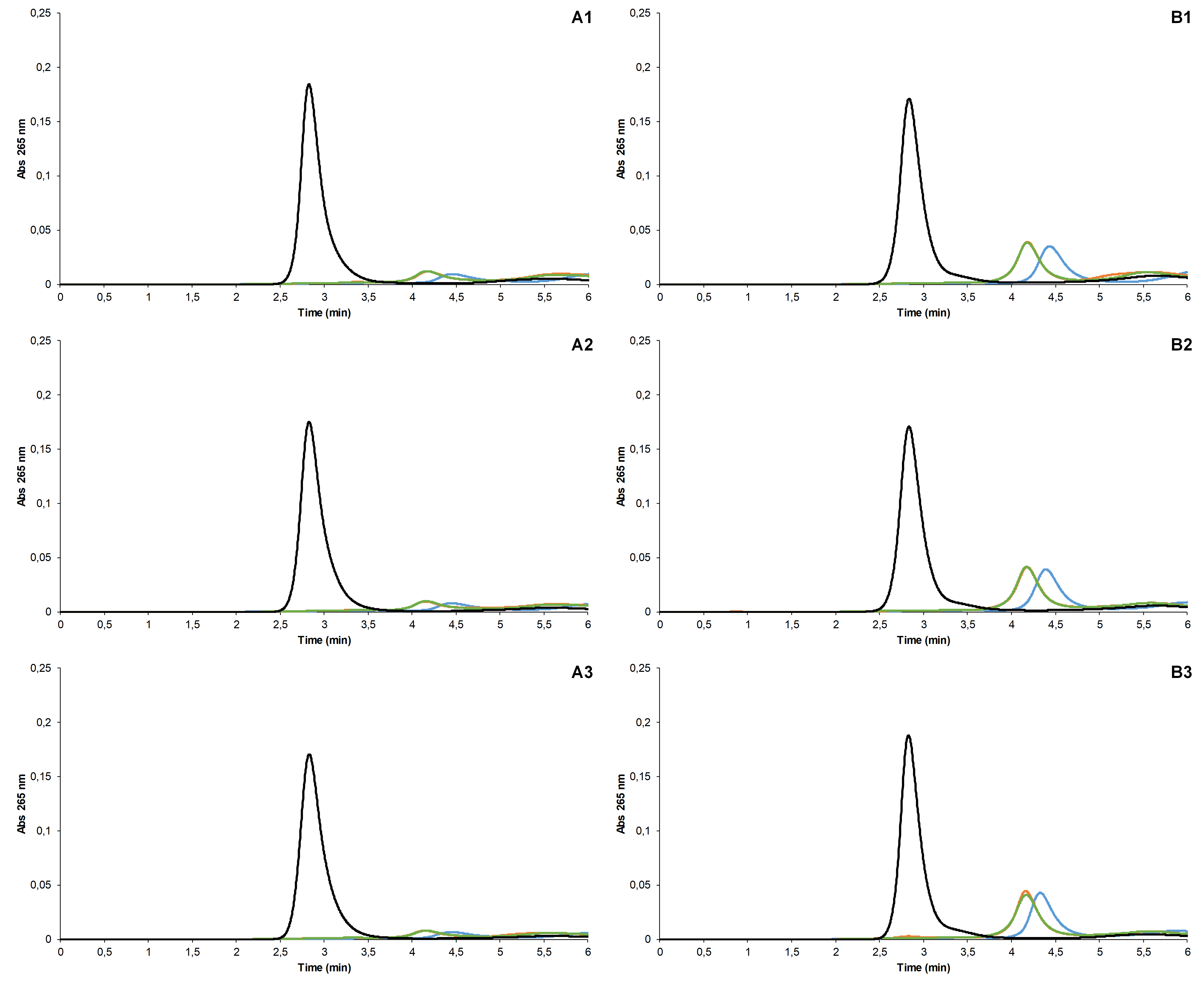

Supplement: Supplementary file 3 — Figure S2. Chromatograms obtained from the analyses of three replicates of fresh (A1-A3) and male-inhabited 7-days old (B1-B3) food. L-ascorbate levels were assessed in homogenates containing 20 mg of food per millilitre of extraction buffer using reverse-phase HPLC, as described in “Material and Methods”. For each condition, individual food samples are represented with a suffix number (1–3). The chromatograms depict three lines representative of the processed technical replicas for each sample (blue, orange and green, respectively), and one indicative of a 25 μM L-ascorbate standard (black). All samples analysed show an absence of L-ascorbate. (TIF 53095 kb) [file 12862_2019_1454_MOESM3_ESM.tif]
