# Supplementary material for: Multiple independent L-gulonolactone oxidase (GULO) gene losses and vitamin C synthesis reacquisition events in non-Deuterostomian animal species
Source: BMC Evol Biol. 2019 Jun 18;19:126. doi: 10.1186/s12862-019-1454-8 (PMC6582580; doi:10.1186/s12862-019-1454-8)
Supplement: Supplementary file 4 — Nucleotide alignment used in the phylogenetic analyses presented in Additional file 2 Figure S1. (PDF 244 kb) [file 12862_2019_1454_MOESM4_ESM.pdf]

```
>Mus_musculus_(house_mouse)_Muridae_NP_848862.1
--------------------------------------------------
-ATGGTCCATGGGTACAAAGGGGTCCAGTTCCAAAACTGGGCGAAGACCT
ATGGCTGCAGTCCAGAGATGTACTACCAG---CCCACATCAGTGGGGGAG
GTCAGAGAGGTGCTGGCCCTGGCCCGGCAGCAGAACAAGAAAGTGAAGGT
GGTGGGTGGCGGCCACTCGCCTTCAGACATCGCCTGCACCGATGGCTTCA
TGATTCACATGGGCAAGATGAACCGGGTTCTCCAGGTG------------
GACAAGGAGAAGAAGCAGGTCACAGTGGAAGCCGGTATCCTCCTGACTGA
CCTGCACCCACAG---CTGGACAAGCATGGCCTGGCCCTGTCTAATCTGG
GAGCCGTGTCTGATGTGACGGTTGGTGGCGTCATTGGGTCTGGAACACAT
AACACCGGGATCAAGCAC---GGTATCCTGGCCACCCAGGTGGTGGCCCT
GACCCTGATGAAGGCTGATGGAACAGTTCTGGAATGTTCTGAGTCAAGT-
--------AATGCAGATGTGTTCCAGGCTGCAAGGGTGCACCTGGGCTGC
CTGGGTGTTATCCTCACTGTCACCCTGCAGTGTGTGCCACAGTTCCACCT
TCTGGAGACATCCTTTCCTTCGACCCTCAAGGAGGTCCTTGACAACCTGG
AC------AGCCACCTGAAGAAGTCTGAGTACTTCCGCTTCCTCTGGTTT
CCTCACAGTGAGAACGTCAGCATCATCTACCAAGATCACACCAACAAGGA
GCCC----------------------------------------------
-----TCCTCTGCATCTAACTGGTTTTGGGACTATGCC------------
------------------ATTGGGTTCTACCTCCTGGAATTCTTGCTCTG
GACCAGCACC------TACCTGCCACGCCTCGTG------GGCTGGATCA
ACCGCTTCTTCTTCTGGCTGCTGTTC------------AACTGCAAGAAG
GAGAGCAGCAACCTCAGCCACAAGATC------TTCTCCTACGAGTGTCG
C------TTCAAGCAGCATGTCCAA---GACTGG----------------
--------------------------GCCATCCCCAGGGAGAAGACCAAG
GAGGCCCTGCTGGAGCTAAAGGCCATGCTG---GAGGCCCACCCCAAG--
-------------------------------------------GTGGTAG
CCCACTACCCCGTGGAGGTGCGCTTCACCCGAGGT---------------
---------------------GATGACATCCTGCTGAGCCCGTGCTTCCA
G---AGGGACAGC-------------------------------------
--------------------------------------TGCTACATGAAC
ATC---------------ATTATGTACAGGCCCTATGGGAAGGAT-----
-GTGCCTCGGTTG---GATTACTGGCTGGCCTAT---GAGACCATCATGA
AGAAGTTTGGAGGCAGGCCCCACTGGGCAAAGGCCCAC------------
------------AATTGCACCAGGAAGGAC--------------------
----------------------TTTGAGAAAATGTAC---CCCGCCTTTC
ACAAG---TTCTGTGACATCCGCGAGAAGCTGGACCCCACTGGAATGTTC
TTGAAT------TCGTACCTGGAAAAGGTTTTCTAC--------------
--------------------------------------------------
--------------------------------------------------
--------------------------------------------------
--------------------------------------------------
--------------------------------------------------
--------------------------------------------------
--------------------------------------------------
--------------------------------------------------
--------------------------------------------------
--------------------------------------------------
--------------------------------------------------
--------------------------------------------------
--------------------------------------------------
--------------------------------------------------
--------------------------------------------------
--------------------------------------------------
--------------------------------------------------
------------
>Xenopus_laevis_(African_clawed_frog)_Pipidae_OCT81467.1
--------------------------------------------------
```

-ATGGTTCTTGGTCGTGGAGGATACAAGTTCGAGAACTGGGCCCAGACAT
ACGGCTCCAGCCCAGAGCTTTACTTCCAG---CCCACGTGTGTAGAGGAA
ATAAAGGAAATTCTCGATCTGGCTCGTCAGAGAAGCAAAAGAGTGAAGGT
GGTGGGAGGCGGTCACTCTCCGTCGGACATAGCCTGCACTGATGACTTTA
TGATCCGAATGGATAAGATGAACAGAATTCTCAAGGTG------------
GATAAGGAAAAGAAGCAGGTAACAGTGGAAGGTGGCATGCTCCTGACCGA
TCTCAACAAGGAA---TTAGATAAGCGTGGCCTGGCTCTGTCCAATTTAG
GAGCCGTATCTGAGGTTGCTGCGGCTGGCGTCATTGGGACGGGCACCCAC
AACACTGGCATCACTCAT---GGCATATTATCAACACAGGTGGTGACTCT
AACACTGATGACGGCATTGGGGGAGATCCTGGAATGCTCCGAGGCTTCG-
--------AACCCAGAAATATTCCAAGCTGCGAGACTCCATCTGGGCTCT
CTAGGGGTGATACTCAGCGTCACTATTCAGTGCAGATCTGCCTTCCACCT
GAAAGAGGTTCCGTTCTCCTCCACTCTCCAAGAGGTCCTCGACAACCTCG
AC------ACTCATCTGAACAGCTCGGAATATTTCCGCTTCTACTGGTTT
CCGCACACAGAGAATGTCAGCGTCTTTTATCAGGACCCAACAGACAAGCC
CCCA--
-----TCTTCCAAAGCAAACTGGTTTCGGGACACTTTT------------
------------------CTGGGAAATTACCTGCTAGAGTTCCTGCTCTG
GATAAGCACT------TTCATAACAGGAATGGTT------CCTTGGATCA
ACCGGTTCTTCTTCTGGCTGCTGTTT------------GCAAGTAAAAGT
GAACAGGTCAACATTAGTCATAAAGTA------TTCAATTTCGACTGCCT
C------TTCAAGCAGCACGTACAG---GACTGG----------------
--------------------------GCTATTCCCATAGAAAAGACCAAA
GATGCATTGATGCAGCTGAAGGGTTGGCTG---GAGAAGAACCCGCAT--
---GTAGTGG
CGCATTTCCCAGTGGAGGTCCGATTTGCCCGTGGG---------------
---------------------GATGATATATTGATGAGCCCTTGTTACCA
T---CAGGACAGC-------------------------------------
--------------------------------------TGTTACATGAAC
ATT---------------ATTATGTACAGGCCTTATGGGAAAGAT-----
-GTTGCGCACCAA---GAATACTGGGTAGAATAT---GAGAATATCATGA
AAAAAGTTGGAGGAAGACCCCACTGGGCCAAGGCTCAC------------
------------ACCTGCACCCGCAAAGAC--------------------
----------------------TTTGAAAAGATGTAC---CCGGCGTTCA
GCACA---TTCAAGAGCATCCGGGAGAAACTGGACCCAACGGGAATGTTC
CTAAAT------GCTTATCTGGAGAAGGTCTTCTAC--------------

>Priapulus_caudatus_(priapulids)_Priapulidae_XP_014666894.1
--AT
GGCCGAATGGGGTATTAAAGGGTACCTATTTGAAAACTGGGCCCAGACGT
ACAAATGCATCCCTGAATTATACTTTACT---CCAAAGACAAAGGAGGAT

GTCATACAGATTCTAGAGCAGGCGCGGGCAGGAGGCAAGACGGTCAGGGC
CGTTGGTGGTGCACGGTCCCCATCAGACATTTGCTGCACCGATGGGTACA
TGATTGATATGCAGCGCATGGATAGCGTGATTGATGTA------------
TGTGCAGAAAAATGTCAAATAACAGTTGAAGGAGGAGTCATGCTGACAAA
GTTGAATAGGATT---TTAGAGGAGAATGGCATGGCTATCAGTGTACTTG
GCGCTATATCTGATATCACGTTGGCAGGAGCCATGGCAACTGGAACCCAT
GGGTCAGGAGCTCAGTAT---GGAGTTCTAGCATCTTACGTTGTGAAGCT
GGAGCTGCTGACTGCGAGTGGTGAGGTGATGACGCTGTCGAGGGAAAAG-
--------GACGAGGACATGTTCCGAGCTGCCGCAGTCAGCCTCGGTGCG
CTGGGCATCATTCTCACTGTAACACTGCAGTGCGAGAAGGCTTTCAAACT
CTGCAAAACGTCGTTCACTACCACAATGGACGATGTTCTTGAGAATTTGG
AG------GTTCATGTCAGGTCTTCTGAGCACTTTCGCTTCATGTGGATT
CCATACACCGGACATGTCATTGCTACGCATGCAAACAGGACTGATAAAAA
AATT--
-----GCGGTGCATTCAAGTTGGTTCTGGGAAACTTTG------------
------------------GTTGGCTACCACATGTTGGAGTTCATGCTGTG
GATAAGCACA------TACTTCACATCTATGGTG------CCATATATTG
CACGCTTTATGTACAATATTTTGTGG------------AGGCATGGTGGA
GAGATGGTAGACACTAGCTACAAGGTC------TTCAACTTCAACTGTCT
T------TTCAAGCAGTATGTGAAC---GAGTGG----------------
--------------------------TCAATCCCAAGGAGTCAGGCCGTG
CTGGTTTTACTGGAGTTACAAGAATGGATATTGGCACAGGAACACAGG--
---CTGCAGG
TACACTTCCCCATTGAGGTGCGCTTCGTGAAGGCA---------------
---------------------GACGATCTTTTGTTGAGCCCATGCTCTGA
A---ATCGATAGT-------------------------------------
--------------------------------------TGTTACATCAAC
ATC---------------ATCATGTTTCGTCCGTATGGCCGCGAT-----
-ACACCACACCAG---GAGTATTGGGACAAGTAC---GAGTCCATTATGA
AGAGAGCCGGAGGAAGGCCCCACTGGGCAAAGGCTCAC------------
------------AGAGAAACAGCAAGTGAT--------------------
----------------------CTGCGCAAGATGTAT---CCGCATTTTG
AACAG---TTCTGCAACATTCGCAGTAGCCTGGATCCACACGGACTATTC
CTTAAT------AACTACTTGAAGAGAATATTGGAATATAAG--------

>Lingula_anatina_(brachiopods)_Lingulidae_XP_013393535.1
--ATGGATAT
ACAGAGTCTCGGCAGAACTGGCCATCTTTTCACTAACTGGGCCACAACAT
ATTCCTGCCAACCTGAACTACATTTTGAA---CCAGAGACAGAAGAACAG
CTCAAACAGATCCTGAACACTGCACAGATCAAGAATAAGAAGGTGAAGGT
GGTGGGTTGTGGTCATTCCTTCTCAGATCTGGTTTGCACTACTGACTATA

```
TGATCAGCATGAAGAACTTTGACAGAGTCATATCAATT------------
GACAAGGAGGCATGTAAAATCAAAGTTCAAGCTGGAATTGAAGTCAAGAA
ACTGAAAGATGAAATTCTGCCACAGCATGGACTGGCATTTAGTGTGTTGG
GGTCTGTGTCAGAAGTGTCCTTAGCAGGGTGTATATGTACTGGAACTCAT
GGCACTGGAGGAAACTAT---GGGGTCTTGTCATCTTATGTGTTGGGTTT
GGAGATCATGACAGCTAATGGAGAGATCATTGAATGCAGTAAAGAGAAG-
--------AACCAGGAAGTATTCTTGGCTGCATGCTGCAGTCTGGGAGCT
CTTGGTGTCATCATGTCTGTCACATTTCAATGTGAACCTGCATTTCACCT
ACATCAGAGTCAATACAGGGACACTTTGAACAATGTTTTGGATAACCTGG
AT------TCCCATATCACCTCCTCAGACCACTTTCGCTTTGTGTGGATA
CCTCACACAGATCACGTAGTTGTGTACAAAGCCAACAGAATATCAGCGCC
CATT----------------------------------------------
-----TGTAGGAAAGACAGTTGGTTCTGGAATTATGCT------------
------------------GTGGGGTGCCATATGTTGCAGTTCTGCCTTCT
CATCAGTGCT------TTCTTCACTTCTCTTCTT------ATTTATATGA
ACCGACTCTTCAGTTGGCTACTGTTC------------TCTAAACCAAAA
GAGATTGTAGATAGGGGTGACAGGGTT------TTCAATCTTGACTGTCT
T------TTCCAACAATATGTCATG---GAATGG----------------
--------------------------GCAGTCCCAAGGGAAAAGACTGCT
GAGGTTTTGTTAGAGCTCAAGGACTGGATA---GAGACAAAC---CAG--
-------------------------------------------TTTCCAG
CACATTTTCCGGTAGAAGTGAGATTTGTCATGGCG---------------
---------------------GATAATATCTATCTCAGTCCAGCATACAA
C---ATGGATTCC-------------------------------------
--------------------------------------TGCTACATTAAT
ATC---------------CTTATGTTCAGGCCATACAACAAGTAT-----
-GTTCCACATGAG---ACATACTGGAATGCCTTC---CAAGATATCATGC
TCAGAGCAGGTGGAAGACCACACTGGGCAAAGGACCAT------------
------------GGTGTTGTAGCTGATCAG--------------------
----------------------TTTAGAAAAATGTAT---CCAAAGTGGA
CAAAG---TTCTGTGAGATTGCCCAAAAAGTAGACCCTCATGCCATGTTT
AGGAAC------CCCAACCTGGAGAGAGTGCTGGGAAGAAAT--------
--------------------------------------------------
--------------------------------------------------
--------------------------------------------------
--------------------------------------------------
--------------------------------------------------
--------------------------------------------------
--------------------------------------------------
--------------------------------------------------
--------------------------------------------------
--------------------------------------------------
--------------------------------------------------
--------------------------------------------------
--------------------------------------------------
--------------------------------------------------
--------------------------------------------------
--------------------------------------------------
--------------------------------------------------
------------
>Gallus_gallus_(chicken)_Phasianidae_XP_015140704.1
--------------------------------------------------
-ATGGTTCACGGCCAAGGAGGATTCAAGTTCCAGAACTGGGCCAAGACGT
ATGGCTCTTCCCCAGAGCTGTACTTCCAG---CCCACCTCAGTGGAGGAG
ATCCGGGAGATCCTGGATATGGCCCGGCAGAGGAACAAGAGGGTGAAGGT
GGTGGGGGGCGGCCACTCGCCCTCTGACATCGCCTGCACTGATGACTTCA
TGATCCAGATGGGGAAGATGAACAAGGTCCTCAAGGTG------------
GACAAGGAGAAGCAGCAGGTGACGGTGGAAGGTGGGATCTTCCTCTCGGA
```

```
TCTGAACGTGGAG---CTGAGCAAGCACGGGCTGGCACTGGCCAACTTAG
GAGCCGTTTCTGAGGTGGCAGCAGCTGGTGTGATTGGGACAGGGACGCAC
AACACTGGGATCAAGCAT---GGCATCCTCCCCACCCAGGTTGTAGGGCT
CTCACTGCTGACAGCCTCAGGGGACATCCTGGAGTGCTCCGAGTCCATC-
--------AATGCAGATATCTTCCAGGCTGCCCGCCTGCACCTTGGCTGC
CTGGGTGTTGTGCTCACCGTCACCTTCCAGTGCGTGCCCCAGTTCCACCT
GCACGAGGTGACCTTTCCATCCACCCTCACTGAGGTCCTCAATCACCTTG
AT------GACCACCTAAAGAGATCCCAATACTTCCGATTCCTGTGGTTC
CCACACAGTGAGAACGTCACTGTCATCTACCAGGACCCCACCAACAAGCC
CCCC----------------------------------------------
-----TCTTCCTCCGCTAACTGGTTTTGGGATTATGCT------------
------------------GTTGGGTACTACTTGCTGGAGTTTCTCCTCTG
GATCAGCACC------TTCGTGCCCAGCTTGGTG------TGCTGGATCA
ACCGCTTCTTCTTCTGGCTTCTCTTC------------AGCTCCCGGGTG
GAGAACATCAATGTCAGCTACAAGATC------TTCAACTACGAGTGCCG
C------TTCAAGCAGCATGTGCAA---GACTGG----------------
--------------------------GCCATCCCCATTGAGAAGACAAAG
GAAGCACTGCTGGAGCTGAAGGCTGCCCTG---GAGAACAACCCCAAG--
-------------------------------------------ATGGTGG
CCCACTACCCTGTGGAGGTGCGCTTTGCTCGAGCG---------------
---------------------GATGAGATCTGGCTGAGCCCCTGCTTCCA
G---AGGGACAGC-------------------------------------
--------------------------------------TGCTACATGAAC
ATC---------------ATCATGTACAGGCCCTATGGGAAGAAC-----
-GTGCCCCGGCTC---AACTACTGGCTGACCTAC---GAGGGCATCATGA
AGAAGTATGGTGGGAGACCACACTGGGCAAAGGCCCAC------------
------------AGCTGCACCCGCAAGGAT--------------------
----------------------TTTGAGAAGATGTAT---CCGGCCTTCC
CCAAA---TTCTGCTCCGTCCGGGATAAGCTGGACCCTACAGGGATGTTC
CTGAAC------ACCTATCTGGAAAAGGTGTTCTAC--------------
--------------------------------------------------
--------------------------------------------------
--------------------------------------------------
--------------------------------------------------
--------------------------------------------------
--------------------------------------------------
--------------------------------------------------
--------------------------------------------------
--------------------------------------------------
--------------------------------------------------
--------------------------------------------------
--------------------------------------------------
--------------------------------------------------
--------------------------------------------------
--------------------------------------------------
--------------------------------------------------
--------------------------------------------------
------------
>Alligator_mississippiensis_(American_alligator)_Alligatoridae_KY043973.1
--------------------------------------------------
-ATGGTTCACGGCCATGGAGGCGTCCAGTTCCAGAACTGGGCCAAGACCT
ATGGCTCCTCCCCGGAGCTGTATTTCCAG---CCGACGTCGGTGGAGGAA
ATCAAGGAGATTTTGGAGCTGGCCCGGCAGCGGAACAAGCGGGTGAAGGT
GGTGGGAGGTGGACACTCGCCCTCTGACATCGCGTGCACGGACGGCTTCA
TGATCCAAATGGGGAAGATGAACAAGATCCTCAAGGTG------------
GATAAAGAGAAGAGGCAAGCGACAGTGGAAGCAGGGATCCTCCTCTCTGA
TCTGAACGTCGAG---CTGACCAAGTATGGCCTGGCGCTGGCCAACCTAG
GAGCTGTCTCCGAAGTGGCGGCCGCTGGGGTGATCGGCACCGGGACACAC
```

AACACGGGCATCAAACAC---GGGATCCTGCCTACCCAGGTGGTGGCGCT
GACCCTGCTGACGGCGGCGGGGGAGATCCTGGAGTGCTCCGAGGGCTCC-
--------CACGCGGACATCTTCCAAGCAGCCCGGGTGCACCTCGGGTGC
TTGGGCGTCGTGCTTACCATCACCTTCCAGTGCGTGCCGGAGTTTTACCT
GCAGGAGACCACCTTCCCCTCCACCCTCAAGGAGGTCCTGGACAACCTCG
AC------AGCCACCTGCAGCGCTCCGAGTACTTCCGCTTCCTCTGGTTT
CCGCACAGCGAGAATGTCAGCGTCATCTACCAGGATCACACCAGCAAGCC
TCCC--
-----TCCTCCACAGGCAGCTGGTTCTGGGACTACGCC------------
------------------ATCGGCTACTACTTGCTGGAGTTCCTGCTCTG
GGTCAGCACG------TTCCTGCCCTTCCTGGTG------CCCTGGATCA
ACCGCGGCTTCTTCTGGCTCCTCTTC------------ACCAGCAAGGGG
GAGAAGGTCGACCTCAGCTACAAGGTC------TTCAACTACGAGTGCCG
T------TTCAAGCAGCATGTCCAG---GACTGG----------------
--------------------------GCCATCCCCATTGAGAAGACAAAG
GATGCCCTCCTGGAGCTGAAGGCCGCACTG---GAGAGCAGCCCCAAG--
---GTGGTGG
CTCACTACCCCGTGGAGGTGCGCTTCGCCCGGGCA---------------
---------------------GACAGCATCCTGCTCAGCCCCTGCTTCCA
G---CGCGACAGC-------------------------------------
--------------------------------------TGCTACATGAAC
ATC---------------ATCATGTACAGGCCCTATGGGAAGGAC-----
-GTACCCCGACTG---GACTACTGGTTGGCCTAC---GAGGGCATCATGA
AGAAGTTTGGAGGGAGGCCACACTGGGCCAAGGCTCAC------------
------------GCCTGCACCCGGAAGGAC--------------------
----------------------CTTGAGAAGATATAT---CCCGGCTTCC
CGAAG---TTCTGCTCCATCCGGGAGAAGCTGGATCCTACAGGGATGTTC
CTGAAC------ACCTACCTGGAGAAGATCCCTTGGGACTTAACCCAATC
CTTGTCCAAGAGCACTGCTCCCCTGGGGTGG---------TTCAGCTTTG
ACCTGGCG--

>Lepisosteus_oculatus_(spotted_gar)_Lepisosteidae_XP_015207781.1

-ATGATACAGGGAACTGAAGGATATCATTTCCAGAACTGGGCTCAGACAT
ACAGTTGCCACCCTGAGCAGTACTTCCAG---CCCAGCTCTGTGGAAGAT
GTCAGACAGATCCTGGAACTCGCTAGGCGTTGCAGCAAGAGAGTGAAGGT
GGTGGGTGGGGGGCACTCTCCCTCAGACATCGCCTGCACAGATGACTTCA
TGATGCGTATGGATAAGATGAATAAGGTTCTTGAGGTC------------
GATGAAGAGAAGAGACAGGTCACAGTGGAAGCTGGGATATTTCTGCGTGA
TCTGAATGAAGAA---TTAGCAAAACATGGCCTTGCCCTCTCAAATTTAG
GAGCCGTGGCAGAGGTGTCTGCTGCTGGCGTAACTGGAACTGGTACCCAC
AACACAGGGGTAGATCAT---GGCATTCTGTCTACGCAGGTCATGCGTCT
GAGTCTGATGACTGCCAGTGGAGAGGTACTGCACTGCTCAGAGTCAGTG-

```
--------AATGAAGACATCTTTCAGGCTGCTAGGCTTCACCTGGGCTGT
CTGGGAGTCATCATCACCATCACCTTCCAGTGCCAGCCTGCCTTTCGACT
TCATGAGCGCCAGTTCCCCTCCACACTCACAGAGGTTTTGGATAATTTGG
AT------TTCCACAGAAAGAAGTCAGAATATTTCCGATTCCTATGGTTT
CCCAATACAGAACATGTCAGAATAATATATCAGGATCGCACAGAAAAGCC
TGCA----------------------------------------------
-----CAGTGTACATCCAGCTGGTTCTGGAGCTATGCT------------
------------------GTTGGATATCATCTACTGGAATTTGTCCTTTG
GGTCAGCACT------TTCTTTCCTGGCCTGGTT------TGCTGGATCA
ACCACCTCTTCTTCAAGTTACTCTAC------------AGCGGCAAAGAG
GAGCGAATAGGCCGCAGTGACAAGGTC------TTCACTTATGAGTGTCT
C------TTCAAACAGCACGTACAG---GATTGG----------------
--------------------------TCTATTCCCATGGAACAGACTAAA
GAGGCTCTGCTGCAGCTGAAGGCCTGGCTG---GAGAATAACCCAGAC--
-------------------------------------------ACAGTAG
CTCATTTCCCGGTGGAAGTGCGTTTTGCTCGGGGA---------------
---------------------GACAACATTCTCCTCAGCCCCTGCTTTCA
G---AGAGACAGC-------------------------------------
--------------------------------------TGCTACATCAAC
ATC---------------ATCCTGTACAGGCCTTATGGAAAGGAT-----
-GTGCCCAAACAG---CAGTATTGGGCTGCTTAT---GAAAACATCATGA
AGGATGTAGGAGGAAGGCCTCACTGGGCAAAGGCTCAC------------
------------AATTGCACCCGTAAGGAT--------------------
----------------------TTTGAGAAGATGTAC---CCTGGTTTCC
AGAAG---TTTTGCTCTATCCGGGAGAAGCAGGACCCATCAGGAGTGTTC
CTCAAC------ACCTACCTGGAAAATATTTTCTTC--------------
--------------------------------------------------
--------------------------------------------------
--------------------------------------------------
--------------------------------------------------
--------------------------------------------------
--------------------------------------------------
--------------------------------------------------
--------------------------------------------------
--------------------------------------------------
--------------------------------------------------
--------------------------------------------------
--------------------------------------------------
--------------------------------------------------
--------------------------------------------------
--------------------------------------------------
--------------------------------------------------
--------------------------------------------------
------------
>Branchiostoma_belcheri_(Belchers_lancelet)_Branchiostomidae_XP_019645195
.1
------------------------ATGGCGGGAGATGCAGACAGACCGGC
AGTGATGACAGGGCAGACAGGAAGAAAGTTCACCAACTGGGCAGAGACAT
TCTCCTGTGAGCCAGAGCTGTTCTTCGAG---CCAACTACTACAGAGGAA
ATAAGACAGATCCTGGAAAAAGCCAGACGTCACAGCAAGCGTGTGAAGGT
GTGTGGGTTTGGGCACTCCCCCTCGGACATAGCCTGTACCACAGACTACA
TGATCAGTCTGGCCAAGTACAGACAGGTGTTGGAGGTT------------
GATGAGGAGAACTGTGTAGTGACAGTCCAGTCAGGAATCCTGCTCAAGGA
GCTGAATGAAGAGGTGTTGCCAAAATACAACATGGCTCTCTCCAATCAGG
GAGCAGTGTCAGAGATCTCTGCTGCAGGGGTCATCTCCACTGGAACTCAT
GGTACAGGCAAGACCTTT---GGCATCTTTGCAACACATGTTCTGGAGAT
GACCATGATGACAGCATCAGGAGAAGTACTGAGGTTGTCTCGTGAGGAG-
--------AACAGAGACATGTTCCTTACAGCATTGGTGGGTCTGGGCTCC
```

CTGGGAATCATCCTCACAGTCAAGATCCAGTGTGAACCTGCTTTTAATCT
CCACCAGGTCCAGTTCAGCTGCTCACTCGATAAGATGCTCAGCACTTTAG
AC------AAGCACCTGGATGAGAATGAACATTTTAAGTTCATGTGGTAT
CCACATACAGATGGGGTGGTGGCATTTCTTTCCAACAGAACAAAAAAGCC
AGTG--
-----CAGACCAATCCCAGCTGGTTTTGGGATTATTTT------------
------------------GTGGGCTACTATTTGCTGGAGTTTCTCCTGTG
GTTGAGCACA------TTTTGTTCAGACCTCCTA------CCATGTATCA
ACAAGTTTTACTACAGCTACATCTAT------------GCTGTACCGTCA
GAGCGGGTGGATCGCAGTGACAAAGTC------TTCAACTTTGAATGTCT
T------TTCAAGCAGTATGTGACA---GAGTGG----------------
--------------------------TCTTTTCCAAGGAGTGAAACTGCA
AATGTGCTGCACACTTTACAAGACTGGGTC---AAGTCCCACCCAGAA--
---CACAAAG
TTCACTTCCCAGTGGAGGTTCGGTTTGTCCGATCG---------------
---------------------GACGACATCTACCTTAGCCCCTGCTACCA
A---CAAGACAAC-------------------------------------
--------------------------------------TGTTACATCAAC
ATC---------------ATTTCTTACAGGCCGTATGGAAAGGAT-----
-GCACCAAAGGAT---GCCTGGTGGGACATGTAT---GAGAGCGTCATGC
ACAAGGTTGGAGGGAAACCTCATTGGGCAAAGGCTCAC------------
------------AAGGTGACACCTGAGCAG--------------------
----------------------TTTCAGAAGCTGTAC---CCAATGTTTT
CCAAG---TTCTGTGCCGTTCGGGAGCAGCTGGACCCACAGGGCATGTTC
CTGAAC------CCCTACCTGGAGCGCCACGTTCTCGGCATGACCCCTCA
GACACAGTTTGACGTT----------------------------------

>Saccharomyces_cerevisiae_S288C_NP_013624_1
---ATGTC
TACTATCCCATTTAGAAAGAACTATGTGTTCAAAAACTGGGCCGGAATTT
ATTCTGCAAAACCAGAACGTTACTTCCAA---CCAAGTTCAATTGATGAG
GTTGTCGAGTTAGTAAAGAGTGCCAGGCTAGCTGAAAAAAGCTTAGTTAC
TGTTGGTTCGGGCCATTCTCCTAGTAACATGTGCGTTACTGATGAATGGC
TTGTTAACTTAGACAGATTGGACAAAGTACAAAAGTTTGTTGAATATCCT
GAGTTACATTATGCCGATGTCACAGTTGATGCCGGTATGAGGCTTTACCA
ATTGAATGAATTT---TTGGGTGCGAAAGGTTACTCTATCCAAAATTTAG
GCTCTATCTCAGAACAAAGTGTTGCTGGCATAATCTCTACTGGTAGTCAT
GGTTCCTCACCTTATCAC---GGTTTGATTTCTTCTCAATACGTAAACTT
GACTATTGTTAATGGTAAGGGCGAATTGAAGTTCTTGGATGCTGAAAAC-
--------GATCCAGAAGTCTTTAAAGCTGCTTTACTTTCAGTTGGAAAA
ATTGGTATCATTGTCTCTGCTACTATCAGGGTTGTTCCCGGCTTCAATAT
TAAATCCACTCAAGAAGTGATTACTTTTGAAAACCTTTTGAAGCAATGGG

AT------ACCCTATGGACTTCATCTGAATTTATCAGAGTTTGGTGGTAC
CCTTATACTAGAAAATGTGTTCTATGGAGGGGTAACAAAACTACAGATGC
CCAA--
-----AATGGTCCAGCCAAGTCATGGTGGGGTACCAAG------------
------------------CTGGGTAGATTTTTCTACGAAACTCTATTATG
GATCTCTACCAAAATCTATGCGCCA---TTAACC------CCATTTGTGG
AAAAGTTCGTTTTCAACAGGCAATACGGGAAATTGGAGAAGAGCTCTACT
GGTGATGTTAATGTTACCGATTCTATCAGCGGATTTAATATGGACTGTTT
G------TTTTCACAATTTGTTGAT---GAATGG----------------
--------------------------GGGTGCCCTATGGATAATGGTTTG
GAAGTCTTACGTTCATTGGATCATTCTATTGCGCAGGCTGCCATAAACAA
AGAA---------------------------------------TTTTATG
TCCACGTGCCTATGGAAGTCCGTTGCTCAAATACTACATTACCTTCTGAA
CCCTTGGATACTAGCAAGAGAACAAACACCAGTCCCGGTCCCGTTTATGG
C---AATGTGTGCCGCCCATTCCTGGATAACACACCATCCCATTGCAGAT
TTGCTCCGTTGGAAAATGTTACCAACAGTCAGTTGACGTTGTACATAAAT
GCT---------------ACCATTTATAGGCCGTTTGGCTGTAAT-----
-ACTCCAATTCAT---AAATGGTTTACCCTTTTT---GAAAATACTATGA
TGGTAGCGGGAGGTAAGCCACATTGGGCCAAGAACTTCCTAGGCTCAACC
ACTCTAGCTGCTGGACCAGTGAAAAAGGATACTGATTACGATGACTTTGA
AATGAGGGGGATGGCATTGAAGGTTGAAGAATGGTATGGCGAGGATTTGA
AAAAG---TTCCGGAAAATAAGAAAGGAGCAAGATCCCGATAATGTATTC
TTGGCAAACAAACAGTGGGCT-----------------------------

--------------------ATCATAAATGGTATTATAGATCCTAGTGAG
TTGTCCGAC---

>Metarhizium_majus_ARSEF_297_XP_014580409_1
ATGCATCCCGCGGCGGAATCCGAGCTGCGAGGCCAAAACGCCAACGGCGT
CGCGTTCCGCGCGAAACCGCACCACGTCCATCGAACCTGGGCGAGGACCT
TCTCCTCGATCCCCGAGCTCTACATCCAG---CCAGAGACCCTCGCCGAG
GTCGAAAAGGTTGTTTCTCTCGCCCAGACATGTCGCCGGCGCATCGTCAC
CACCGGCTGCGGCCACTCGCCCTCCGACATCACCTGCACCTCCAGCTGGA
TGGTCAACCTCGACCGCTTCAACAAGATCCTTGCCGTC------------
AGCACGGACACGGGCCTGGTGACCATGGAGAGCGGCATACGGCTGTACGC
CCTGTGCGAGGAG---CTGGAGCGTCACGGTCTTGCCATGCCCAACTTGG
GGAGCATCAACCAGCAGTCCATTGCTGGCGCCATTTCGACGGGAACCCAC
GGGAGCAGTGTGCGCCAT---GGCCTCATGTCTGAAGATGTGCAGTCTCT
CCGAATCACACTGGCCAATGGGACAACGGAACTCTGCTCTGCCGAGTCG-
---------AATCCGGAGCTGTTTCGAGCCGCGCTCCTGTCTCTCGGCGCA
TTGGGCATCATCACCGAGATTACGCTTCGCGCGGTCCCCGCCTTCAAGCT
CAAGTGGACGCAGACCATCGACACCGACTACAAGCTGTTCAAGAGATGGC
CG---CTCGACCTGTGGACGCAGAGCGAGTTTGTCAGGGTGTGGTGGTTC
CCCTACACGAGACGCGCCGTCGTGTGGCAGGCAGACAAAACCACGGAACC

CGAG--
-----CAGGACCCCCGACGAGCTACTACGACGGATCC-------------
------------------CTGGGGTACTACGTCTACCACAACCTGCTCTA
CCTGGCGCAG------TTTGCTCCCCGGATCCTC------CCCTGGGTCG
AGTGGTTCGTCTTCGGCATGCAGTACGGCTTCGCCAACGGGTCGACCACG
ACGGCCGTCCAGCCGAGCCGAAGGGCG------CTGCTGATGAACTGCCT
C------TACTCGCAGTTTGTGAAT---GAGTGG----------------
--------------------------GCGATCCCCCTGCACAGGGGGCCC
GAGGCGCTGCGGCGACTCAGCGCCTGGCTGAACCGCCTCACGCCCGACGA
CCCGGACTACGTGGCGCACGGCATCCCCTTCTCGGCCGACGGGCTGTACG
TGCACGCCCCCGTCGAGGTGCGGGTGAGCGACACG---------------
------AGCCTCACCTCCAACCTCCGTCCGTACCTGGACCCGACTGTCCC
CGATGGCCCGACG-------------------------------------
--------------------------------------CTGTACCTCAAC
GCC---------------ACCTTGTATCGGCCGTACTGGCGGGAC-----
-CCGCCGTGCCGCGACCGCTACTACCAGGCGTTT---GAGTGGTTGATGA
GGGACATGGGCGGGCGGCCGCACTGGGCCAAGAACTTT------------
------------GAGACGTGTCGGCCTGAT--------------------
----------------------ATCGAGGCCATGTATGGCGAGAGCTTGG
AGGCG---TTTCGGAGGATACGCGACGGCGTGGACCCGGAGGGCATGTTT
GTCGGC------CCGTGGCACAGAGAACGCATCATGGCACCTCTGCCCGG
ATCCAAGCTTCAGCTGGAAGAGGTGGAGGTT---------TCAAGGAGGA
AGGCTGCTGGCGGCGGTCTCGTGATGGAGGGGCTTGTCGGAGGAGGT---

>Nematostella_vectensis_(starlet_sea_anemone)_Edwardsiidae_EDO44935.1
------------------------ATGTCAAGCTTGGAAACAAAGAAGGT
TTTGGCGCGAGGAAAAAAGGGACACCGGTTTACCAACTGGTCTACTACAT
ATTCTTGCTCCCCTGAATTATACTTCGAG---CCAGAATCCACCGATGAA
ATACGGCAGATTTTGCTAATTGCAAAGGAGCTCCATAAGCGTGTGCGTGT
GGTAGGGTCTGGCTACTCCCCGTCTGATCTTGCTTGTACCCCAGAGTATA
TGATGAGTTTGAAGAAGTACAAAGCTGTTTTAGAGATT------------
GACCAGGAGAAACAGCAAGTAACAATGCAAGCAGGGCTCACACTCACCGA
GCTCCACACCGTG---TTGGACATTCATGGACTTGGATTCCCAAACCTTG
GTGCAGTGTCGGGTATCTCCTGTGCTGGGGTGATCTCCACCTGTGTGCAT
GGAACTGGGTTCAACTAT---GGTCTCCTAGCAACACATGTTTCAAGACT
GGATATAATGACGGCAAGTGGAGAAATTATCACCTGCAGTAGAATGCAT-
--------AATGAAGACATCTTCAGATCTGTCATTTGTGGGATGGGTTCA
GTTGGTGTTATTCTTTCCATTACATGGCAGTGTGAGAAGACCTTTAGTCT
TTGCTTAAACAAGAAATCCTTCTATTTAAATGAGATGCTTGAGGACTTGG
AG------GCACAGCTTGTGTCCTGTGATCACTTCAAGTTCTTTTGGTAT
CCACACACTGACAAAGTTGTTGCAGAATATGCAAGTAGAACAACAAGGGG
CCAT--
--ACTCCTCCACAGAATAGCTGGTTCAGGGACATGTTG------------

------------------ATTGGGTTTTATTTGCTGGAGTTTCTTTACTG
GTTAAGTATT------TTTATCCCAGCTCTTGTG------CCTCTGATAA
GCAAGTTGTATTATATGATAGCAACA------------TCGGGTAGCAAG
GAGAGAATTGACAGAAGCTACAAGATT------ATGAATTTTAATTGTTT
G------TTCAAGCAGTATGTGACA---GAATGG----------------
--------------------------TGTATTCCAAGAAATAAAGTTGCA
GATGTCTTGAGGACGTTACGAGATTGGACT---GAGAAAAGT---GGA--
---TACAAAG
TCCATTTTCCTGTTGAAGTTCGCTTTGTGAAGGCT---------------
---------------------GATGATTTCTATCTGAGTCCATGCTACAA
G---ACTGATTCC-------------------------------------
--------------------------------------TGTTTCATTAAC
ATC---------------ATCTGCTACAGGCCATACAACCAGTTT-----
-GTTGCCCATGAT---GCCTACTGGAGATTCTAC---GAGAACCTGATGG
ATTCCGTTGGAGGCAAGCCTCACTGGGCTAAGGCTCAT------------
------------AATCTGTGTGCCGCTGAC--------------------
----------------------ATGGAAAAGAAGTAC---CCGATGTTCA
ACAAA---TACCGGGAAGTTTGCCAGAGGCTAGACCCACAAGGAATCCTG
AGGAAT------TCAAATGTAGACTGCACCATTTTTGGTAAAACT-----

>Parasteatoda_tepidariorum_(common_house_spider)

-ATGAAGACCGGAATTCAAAATATTCATGTAAAAAATTGGGCTGAAACAT
ATGAATGTAGACCAGACCTTTTCTTTGCT---CCAAAAACAGCAGAAGAA
ATATCTCAGATTTTGGACGTAGCTAAGCAAGAAGAAAAACGAGTAAAAGT
TGTTGGTTGTCGACATTCTCCTTCAGACATAGCTTTTACCAATGGAATTA
TGATAAGTTTGCAACACATGAATAAAGTTTTAGAGATT------------
GATAAAAACAGTAAAAGAGTTAAAGTTGAAGCTGGAATCCTTTTGAGAGA
TTTGAATAAAACT---CTTTATTCGAATAATCTTGCATTGTCAGTACTTG
GCTCTGTATCTGACATAACACTTGCTGGTGCAATGTGTGTTGCCACTCAT
GGGACTGGTTTAGAATAT---GGTACCATATCATCTTATGTCACAGAATT
GGAAATGATGCTGGCAGATGGTTCTGTAAACACATATTCAAGTGTAAGA-
--------GATGGAGATATTTTTCAGGCAGCTCTGTGCAGTCTTGGGTCA
CTAGGAATTATTTTGACTGTCACTATACAATGTGAAGAAGCTTTTAATTT
ACAAATGAGGCAATATGGTCTAAGTTTAAAAGATGTTATTGAAAACCTAG
AG------GTACATCTCAATGGTTCCGATCATTGCCGATTTATGTGGTTT
CCATACACAGATAGTGTTGTCATGTCTCATGCAACTCGTACAGAATTACC
AGCC---------------------------------TTGAAAGTAACAT
GGATAAGCAAGATATGGAATATGATATTTGATTATGGT------------
------------------GTTGGTTATCACCTTTTAGAATTTTGTTATTA
TATAAGCACG------TTTATGCCTCATATAGTA------CCATACATAA

ATAGATTCTTTTACTACACAGTTTTC------------TCTGTTTACACT
AGAAAAATTGATAGAAGTTATAATGTG------TTCAACTTTGAATGTCT
C------TTTAAGCAGTATGTTAAT---GAATGG----------------
--------------------------GCTATTCCTATTGAAAAAACTGGC
GCTGTTCTATGGGAACTTAGAGAGTGGATT---GAGACTACCCCAGAA--
---GTATATG
TTCATTTCCCAATAGAAGTTAGATTTTGCAAAGCA---------------
---------------------GACAACATTTTCCTTAGTCCAGCTCATGG
A---AGAAATACA-------------------------------------
--------------------------------------TGTTATATTAAT
ATT---------------TTAATGTACAGGCCATATGGGAAAGAT-----
-GTACCTTATGAA---AAATATTGGGCAGCATAT---GAAAAGATAATGA
TGGAAGCTGATGGCAGGCCTCATTGGGCTAAGGCACAT------------
------------TCAGTGACAGCTGATAAA--------------------
----------------------TTTAGACTGATGTAT---CCCTACTTTG
GAAAA---TGGTGCTCAATACGACAAAAGTTGGATCCAACAAATATGTTT
TTTAAT------TCTTACATGGCAAGGATTTTTTCTCATAGTAGACTA--

>Folsomia_candida_(Hexapoda)_1

-------TTTTACTTCCTCAGGAACTCGTACAGATCATATCAAAGTTCGA
TTTACTGCTCTGCCACATCTCCCATGCTGTATCCAACGTCCGTGACAGAA
GTCCAGCAAATAATACGAGACGCCATCAAAAATGGGAACACCGTAAAATC
CGTTGGGAGCAGACATTCAATAACCGATGTGATTTGCACTGACGGGATTC
CAATGTCAATGGAAAATATAAAGTACACGATTGCC---------------
---AACAACGATGAAACTGCCACGGTTGGCGGAGGGGCGGAAATGAAAAG
CGTCTTGGAATTT---CTCCACGCTAGGAATCGAACTCTAATTCATGTTC
CAACTTTTGGGGGAATAACGGTGGCTGGTGCAATCGGGACAGGAGCTCAC
GGGTCCTCGCTACTCCACCCCCCGTCTCTTTCAGATCAAATCGTGCGACT
CACAATTGTGGATGGCTTAGGAGAAATTCGAGTCATTGAT----------
--------GATCCCCACGAGGTCAACGCGTTCCGAGTGCACTTGGGCCTT
TTAGGTGCAATTGTTGATGTAACTCTGAAAACCGTTCCACTGTTCAAAAT
GGTTGTAAGAAATTATGTAGAAGGAGAAAATATTCTTTTTGATGGGCAAG
CA---ATCAAAATGGCGGAAGAACATGACTGGTTTGAAATGTGGTGGTTC
CCTTCGACAAAGAACGTCGTTGTATCGAAAGGGAACTACACCAGTGATTT
GGAAACTGTT---------GGAAATGCTACCACTAAT---TTAATCCCCG
ACCAAAACAGCGCAGAAATCATGAGTGCACGGGACGCGTTTGAAACTCTT
CAAGGAACTCGAAATAAACGGGGATTGTACGCTATCCAGGATTTTACTCA
GTTTTCAATT---------TACCAAGACGTAGTAGGAAAACCCGCTTTCT
TTACCGAGGATGGAAGA----------------------ACTATTAAAAC
CCAGCTACAGGTTATGCATGGCAATTG------CAATCAAATAAGTGC--

--ACCAAC
AAGTTCATAAAAACGATGAAGAGCATTTTA---TCCCAAATCCAAGCA--
--ACATTA
CCATGATTGGCGTATTCATCAGGTTTTCGCCCCCT---------------
---------------------TCTGACGCACTCATGGCAATTTCCAGTGG
A---CGCCGAACA-------------------------------------
--------------------------------------GTAACAGTAGAG
TGG------GCTACTCCCATGAGAAGAGACCCTTATGGAACGTCC-----
-CGCGATGGTATTGGAGCCTATCAAGCAATTCTA---CAGGCGATGGTGA
AACAACACGGTGGTCGTCCTCACTGGGGGAAAAACGGCTG----------
------------TACTATTCCAACCCTGAT--------------------
----------------------ATCATCAAAGCAACCACACCAAACCGAG
AAGCT---TTCATAAAGGTCATGAAGGCATACGACCCACAAGGACTATTT
CTCAAC------AAGTTCGGTCGACGACTGCTCGGTGTGTCCAGAGAGAT
GGACGTGGATCCAAGTGTGAAACGTTGTGCG---------TTGCAAGACT
ACTGCATTTGTTCCAAGCACTCGGATTGTGCTCCGCTGCAGCAGTGCACA
ATTGTCAACGGGTATGCGGTTTGCAACGAAATTGGTGCTGGATTTCTGCC
T------------------------CCCACAATAAAAAGTTACAATCCTA
CCAATCTGACATCCATTTATGAAGCTATCGAGAAATACTCGTTTCATTCC
GAAACTATTGAAGGGGATGACGCAGAAGCAGCTCCACAATTTTCT-----

>Folsomia_candida_(Hexapoda)_2

--TCGG
TTATCTGCTACCCTAAAACCGCAATACAATATCCCTCAAGCATATCCAAT
GTCGTTCGCCTAGTAAAAAACGCGAGGAAGGATGGCGTACCTATCAAAGC
GATAGGGTCACGACATTCCATAACGGATATCATCTGTACGGACGGTACTC
CAATATCACTGAAACTTTTGACCAAAAAGTACTAC---------------
---CACGAAGATAACACTGCAACTTTTGAGGCTGGCATAAAATTGGACCA
AGTTTTATTATTC---TTACAAGAAAGGAATCGTACTTTGATTAATATCC
CG---ATTTACAGGATCACTCTCGCCGGTGCAATAGCAACGGGCGCACAT
GGATCATCTCTCAAACACCCAGCATCAATTTCAGATCAAATAATCGGGCT
TCAAGTTGTTACCGGAACTGGTGAACTGGTCAACATAAAC----------
--------GATACAAAAACACTGAAAGCATTCCGAGTTAACTTGGGACTT
CTTGGAATCGTGGTCAAAGTCACGTTAAAAATTGTCCCCATTTTCAAAAT
GTCTATTCAAAACAGTGTGGAAGAAATAGATATATTAACGAGTGGGAAGG
CC---CTAGAATGGGCGCGAGAATATGATCATTTCCAACTCTGGTGGTTC
CCATCCCTGCAGGAAGTCGTGGTCGCCAAGGGAAATTATTTACCATTTCT
CAAAAATACCACTTTCGAAGGTAACGCTACTAACAAC---CTGATTACCG
AAGTCACCCAATACACTGCCCGATTTGCTGCGGTCCTCTACGAATTTTGT
AACTGGGCTCAG------ATAGGATTGACCATCTTGGAGGGATACATGAG
AGATTTCTTACTTAAAAGTATCCCCACAGCAAGA------CCACTTTACG
AAGAGGGGGC----------------------------ACGTTTAAAAAT
CCCGCCGTGGGAATAGGTTGGCGTCTC------CTGTCAAGCCAATGCAG
ACCTGGAACGTGCTGGTGGACAAT---CCCTGGGCGACC-----------
--GTGGGCGGGACGGAATCTGCCATCGCGTTTGACACCAAGCATTTCAAA

GGAGTGATGACCATGATTCAAGACACGCTA---CGGGAAATGCCCACG--
---TGTTTCT
ACATGTCCGGAATCCTGATCAGATTTATGCCCAAG---------------
---------------------TCTGAAGCATACCTGTCCTTGTCGAGTGG
G---AGAGAGACG-------------------------------------
--------------------------------------TTCAGCGTGGAA
TGG------GTGAACCCGTTGAGGACAAGGAGGCACTTGGATGCC-----
-GTCGTGGGACTGGGGACATTTCAAGCTATTTTG---CAAAGGATGGTGC
GAGACTTTGACGGGATTCCTCACTGGGGAAAAATGGGCTG----------
------------CATTTCTTGGGGGAAAAT--------------------
----------------------GTGCTCAAG---------AAAAATAGAG
ACAAT---TTTATTGCGGAGATGAAAAACTTTGATCCGGACGGCGTCTTT
CTTAAC------AAGTTTGGTAGGAGACTGACTGGGGAGATTGGTGAGGC
TGACGTCGACCCGGAAATGGAGAGGTGTGCT---------TTGCATGATT
ACTGCATTTGTGGGAAGGATTACGATTGTGGGGTGGGACAAAGGTGTGAA
AAGTTAAGCGAT------GGGGTACTAGTTTGTCGAGATCAGGATAATGT
AATTCTTGGCGGACTGGGTTTCGTGTTTGGAACTAACTTTTTAGCCTTCA
TCTTGTACAGATTATTTTACAGG---------------------------

>Orchesella_cincta_(Hexapoda)

--------------------------CAA---CCTAGATCTATCGCCGAT
GTCCAGGACGTGATTAAGCGTGCCAGGGAGAAGAATCAGACAGTGAAAGC
GTTGGGAAATCTGCACTCGATAACTGATTGCTTTTGTACTATCGGTGTAC
CGGTAAATATGACCCACATAAATCATACTTCTTACAAC------------
---GCTTCCAGTCAACTGGTGACAGTAGGTGCTGGTTCAACTTTACATGC
CCTTCAGGAATAT---CTCCATGACCTGGGGAGAGCATTGTCGGATGCCC
CTTCATATGGGGACATTACCGTTGGAGGTGCTTTGGGCACAGGTGCTCAT
GGTACTTCACTCCGATTCCCATCAAGCATATCCGACCAGCTTCATTCTGT
AACTGTAGTGGACGGTCGGGGCAACGTGAAACACTTCACCGAA-------
--------GGTGATGAAGACTTTAACGCATTTCGGGTTCATTTAGGTCTT
GTTGGAGTAATTGTGGAAGTCGTTTTAAAAACTGTGGAGCAGTTCAAACT
TAAAGTTCGTAATTACCCTGTTAGCGACGACATTTTATGGACAGAAGGAG
AAATATTAAAGTTGGCAAAGGAAAATGATCGATTCCAGTTTTGGTGGTTT
CCTTCATCCCAAAAAGTAGTCCTGTCTCTCGGAAATCGAGTACCTGTTGA
TGAG---------------CCAGGAACCTGTGAAACTCACTTTATATCTG
AAATGCCGAAACCTATCAGTGTGGGCTTTTCAATGGCCTTGGAACTCATG
CAAAAGACGAGGGATCGTGTGGGATTCTTTTTAGTTCAAGAGTATGCAAG
AGTGGGACTTTATGAACAAGTTCCAGGAAGAGAT------CCAGTGTATT
CTGATGAAAATACGAGAGTCATTTGC------------TCCCCC------
GAAGCGGTGGGATACAGTCACCGAATG------GTCACCAACAGATGTAA
T------GAGTGCTTCTGGAAACAA---GGGCCGTTGTCA----------
--CTGCTAGATGATGATGCAAATATTGCATTGCCACTTGAGGAGCTACCC
GAAGCGCTCAAAACTATTAAAAACATACTA---AACGCATATGAAGCT--
---CACTTCC

CCATGAACGGGATTTACTTCAGATTTTTGACCAGT---------------
---------------------TCTGATGCTTTCATAGCTATAAACAGTGG
C---AGGCCTTCG--------------------------------------
--------------------------------------GTGAGCGTTGAG
TGG---------------ATAACGGCTCCAAGATATGATAAATCCCACAT
GAAGTTGGGGTTGCCAGCATACCAAGCAATCGGC---CAGGCTCTGATTA
ACGAGCATCAGGGTCGTCCACATTGGGGCAAAAATGGACTA---------
------------TGTTACAGCAGCAGAGAT--------------------
----------------------ATCCTTAAAGCAGCTCACCCTCGACTTC
CAGAC---TTCCGGCGTGTGATGAAGACAATGGATCCTGACGGCATCTTT
TTAAAT------GACATGGGAAGGCGAATACTTTTCGAAAGCAATCAGCT
AACTGTGAACCCCGAACTGAAGAAACATTGT------GGACTTAATGACG
TCTGCTTTTGTAAATCAGATTGGGAGTGTGGAAACAATGGAAACTTTCTA
CTTGGTCTTGGTAGCTCTTCTTCGACTTGTGGAACTTTAAGGGGCTACAA
A------------------------GTGTGCAAAGAAAGAAGCCTTTTGG
GCGCACTGACGAAAACTGTTCCAATCACACAAACTCTCAGCATGCTCACA
GGAATTGGGAATAATCCGGAGCAAATTTTTAATGCGTTATCAAAGTACAA
AATGCTTCAACACATTTTTGCAACAAAG----------------------

>Holacanthella_duospinosa_(Hexapoda)_1

--TCACTCGAACCTCCACGTTAC---------CCCACAACATATCAGGAA
GTAGTGTCTATTGTCAAAGAGGCCATCAAGAAAGGTGTGAGAGTGAAAGC
TTTTGGTGCTCGTCACTCATCCACAGATATAATTTGTACTGATGGGATCC
CAATTGACATGCATAGAATACGTGAA---TTGAGTCTT------------
---GATGAGAATAACATTGTGACCGTGGGAGCAGGCGTGACAGTTGGGGT
TATGACAGAATTT---TTGATGGATCATGGACGAGGTCTTCCAATATTAC
CAGCATTTCAGAATATCACAGTAGGAGGGGCAATCGGAACAGGTGCTCAT
GGGAGTTCATTCAAGTACATTGCTTCACTTTCTGATCAAACGATCCAAGT
ACTTATCGTCGACGGATTAGGAAATATTAGAAGTATTGATTCC-------
-----------GAAGAAGATTTGAAAGCATTTCGTGTACATTTGGGACTT
CTTGGAATCATTCTTCATGTCAAGTTCAAAACCATTCCACTTTTCAAATT
TAGAGCTCACAATTATGTAATGGAAGAAGAAATTTTGAGTGGGAAAATCA
TT------GATATGGGATTGGAGACTGACCAAATTATGTTCCATTGGTTC
CCATCTTTTCGTAAAGTTGTAGTGGCCAATTGGACGAGAGTGGATGACCC
GAACACCCCT---------GGAAATGCTCACACAAATGCTCTAATTCCTC
CAACCACTGCATCGTTCAATTACTTTGGGAAACGAGCAATGGAGGCAGCA
CAGGCATCCAACTCAATCCAAGTAATGAATTTCTTCCAATATGTCAGTTT
TCTTGCCCTATTTAAAACAGTTCCGGATGTTCTA------CCGATTTATA
CAGAAAATAATATTCATGTGATAAAT------------------------
CCAGCGATTGGATACTACCATCGCCTA------TCATCAGTTGGTTGCTT
G------AATAAGGGTTGGGTCCAATGTCCTTGGGAACATGGGAAAGACG
GAGTCTATGCCATGGATAACGAAATGGCCATTGATATCTATAATCTCCCA
AAATTTGTTACCAGAGTTAGAGACATTTTG---TCAAAATCTTCAGCT--
---GTATTCC
CTTTTGAAGGGATCTTGATTAGATTCTCTGGTTCA---------------
---------------------TCTGATGCTCACATGTCACTGAGCCGAGG

T---CGAAAAACA-------------------------------------
--------------------------------------TGTCACGTAGAA
TTC------TATCCTTTCGAAAGGAAGAATCGATACATGGATCCA-----
-ATTGTAGGGATGGCAGCTTACCAAGCAATTATG---CAATCTCTTGTCA
ATGAGTTCCAAGGACGATCACATTGGGGTAAATCAGGGGCTTAC------
------------TACCATACAACGGAGCAA--------------------
----------------------CTCCGACGGAAACTTGACCCTAGTGCTC
GTTCTGCATTCATAACTATAATGAAGGCATTTGATCCCAATGGACTTTTT
CTCAAT------GACTTTGGAAGACGACTGTTGGGGGAAAGTACAAAACA
AAACTGGGATCCTCTAGTCAAACACTGCGCT---------CTGCTCGACC
ATTGTCTCTGTTCCTCTCAGAATGACTGCGCATTGGGTCAAAATTGTTCA
CGAATAGACGGCTATCCTAACTACCCCGTCTGTTTACCAAAGAATCAACG
A------------------------CATAAGACTCGACTCTTTGATTTCT
CACATTTTACCATCTATTACAACCAA------------------------

>Holacanthella_duospinosa_(Hexapoda)_2

--TCACTCGAACCTCCACGTTAC---------CCCACAACATATCAGGAA
GTAGTGTCTATTGTCAAAGAGGCCATCAAGAAAGGTGTGAGAGTGAAAGC
TTTTGGTGCTCGTCACTCATCCACAGATATAATTTGTACTGATGGGATCC
CAATTGACATGCATAGAATACGTGAATTGAGTCTTGAT------------
------GAGAATAACATTGTGACCGTGGGAGCAGGCGTGACAGTTGGGGT
TATGACAGAATTT---TTGATGGATCATGGACGAGGTCTTCCAATATTAC
CAGCATTTCAGAATATCACAGTAGGAGGGGCAATCGGAACAGGTGCTCAT
GGGAGTTCATTCAAGTACATTGCTTCACTTTCTGATCAAACGATCCAAGT
ACTTATCGTCGACGGATTAGGAAATATTAGAAGTATTGATTCC-------
-----------GAAGAAGATTTGAAAGCATTTCGTGTACATTTGGGACTT
CTTGGAATCATTCTTCATGTCAAGTTCAAAACCATTCCACTTTTCAAATT
TAGAGCTCACAATTATGTAATGGAAGAAGAAATTTTGAGTGGGAAAATCA
TT------GATATGGGATTGGAGACTGACCAAATTATGTTCCATTGGTTC
CCATCTTTTCGTAAAGTTGTAGTGGCCAATTGGACGAGAGTGGATGACCC
GAACACCCCT---------GGAAATGCTCACACAAATGCTCTAATTCCTC
CAACCACTGCATCGTTCAATTACTTTGGGAAACGAGCAATGGAGGCAGCA
CAGGCATCCAACTCAATCCAAGTAATGAATTTCTTCCAATATGTCAGTTT
TCTTGCCCTATTTAAAACAGTTCCGGATGTTCTA------CCGATTTATA
CAGAAAATAATATTCATGTGATAAAT------------------------
CCAGCGATTGGATACTACCATCGCCTA------TCATCAGTTGGTTGCTT
G------AATAAGGGTTGGGTCCAATGTCCTTGGGAACATGGGAAAGACG
GAGTCTATGCCATGGATAACGAAATGGCCATTGATATCTATAATCTCCCA
AAATTTGTTACCAGAGTTAGAGACATTTTG---TCAAAATCTTCAGCT--
---GTATTCC
CTTTTGAAGGGATCTTGATTAGATTCTCTGGTTCA---------------
---------------------TCTGATGCTCACATGTCACTGAGCCGAGG
T---CGAAAAACA-------------------------------------
--------------------------------------TGTCACGTAGAA

```
TTC------TATCCTTTCGAAAGGAAGAATCGATACATGGATCCA-----
-ATTGTAGGGATGGCAGCTTACCAAGCAATTATG---CAATCTCTTGTCA
ATGAGTTCCAAGGACGATCACATTGGGGTAAATCAGGGGCTTAC------
------------TACCATACAACGGAGCAA--------------------
----------------------CTCCGACGGAAACTTGACCCTAGTGCTC
GTTCTGCATTCATAACTATAATGAAGGCATTTGATCCCAATGGACTTTTT
CTC------------------TGTCGTGCACTTCCGGCTCCAAATATCAA
TTTCCGAGCATCGTCGATTAGTAGATGTTCAATTCGGATTCTCTTGTGGG
CAACGCTTTCA---------------------------------------
--------------------------------------------------
--------------------------------------------------
--------------------------------------------------
--------------------------------------------------
--------------------------------------------------
--------------------------------------------------
--------------------------------------------------
--------------------------------------------------
--------------------------------------------------
--------------------------------------------------
--------------------------------------------------
--------------------------------------------------
--------------------------------------------------
--------------------------------------------------
--------------------------------------------------
------------
>Eisenia_fetida_(Annelida)
--------------------------------------------------
--------------------------------------------------
--------------------------------------------------
--------------------------------------------------
----------------------------CTTGTCGCGACCGGCGGACTGC
TCATCGACCTCTCCGGCAATACCGGCGTCACCGGCACC------------
GACAAAGCGCGCCGCCGCGTACGAGCAAAAGCAGGCACACCGATCCACGC
GTTCGGCGACAGA---ATCTGGGCAGACGGCCTCGCACTGAAGAACCAGG
GAGACATCGATAAGCAGAGCATCACGGGTGCACTGTCCACCGGAACGCAC
GGATCAGGGATCGGGTTG---GGTAGCCTCTCGTCCGAACTGCGTTGGGT
GCGTCTCATTAACGGCCATGGCGATGTCGTCGAGATCTGCGAAGAC----
--------CAGTTCGACCGGTTGCGCGCCGCGCAGGTTGCACTCGGCACC
CTCGGGATTTTCCTTGAGGTTGAGCTGGAGGTCGCCGAGGCGTACTGGCT
CCAAGAGACAATCACCTATCCGACGTGGGACGAGACGGCATCAACCTGGG
AC------CACGACATTGACAATAACCGACACTACTCGTTCCTGTGGTGC
CAGGCTGAAGAATCCGCCCCCCTCTACGAGCTGCCCACGCCCGACTCACA
GTCC---------------------------------------------A
TGGTAAACCGCAGCTACACCAAACGGTACAACGCGGTT------------
---------------------------CACCTCAATGAGGCC--------
----------------------------------------CCCACGACCT
CCCAC------------------------------------ACCGAGGGT
GCACGCTGTGATCGCTCCTACCGGATC------TATCCGGGCGGGTTCAT
G------CTCCCGTTCCATGAACTC---GAGTAT-----------------
--------------------------TACGTACCCCGTGAAAACAGCCTC
CCGGCGGTTGAGGCCCTGCAAAACCTTATC------CGCACACGTCAC--
-------------------------------------------TCTGACC
AGAAATACCCCATTGAGGTCCGCTGGGTGCAGCAG---------------
---------------------GATGACGGTTACCTCTCTCCTTTTTACAA
A---CGCGACACA-------------------------------------
--------------------------------------ACATCCCTCTCG
GTGTCGGGAGCACCCGGCACACACTACTTCCCCTACCTGCGTGAT-----
-GTT---------------------------------GATGCACTCTTGT
```

```
CGGACTTCAACGGACGCGCTCACTGGGGCAAGCTCCAC------------
------------TTCCTCACCCGCTCACGC--------------------
----------------------CTCGAAGCGCTGTAC---CCCGAGTTCG
ATGCG---TTCCTCGCGGTCCGCCGTGAGTTCGATCCCCACGGAATCTTC
CTTAAC------GATCACACGCGAGCTCTCCTCGCC--------------
--------------------------------------------------
--------------------------------------------------
--------------------------------------------------
--------------------------------------------------
--------------------------------------------------
--------------------------------------------------
--------------------------------------------------
--------------------------------------------------
--------------------------------------------------
--------------------------------------------------
--------------------------------------------------
--------------------------------------------------
--------------------------------------------------
--------------------------------------------------
--------------------------------------------------
--------------------------------------------------
--------------------------------------------------
------------
>Capitella_teleta_(Annelida)
--------------------------------------------------
----AATACTGGAAAGAGAGGCCATTTATTCGAGAACTGGGCAGGCACCT
ACTCATGTTCCCCGGAAGTATATTTTGAA---CCAACTAATGAAGAAGAG
GTGCGCCAGATTCTTCAAGTTGCTCGAGATGAGGGCAGGTGCGTCAAAGT
GGTTGGAGGGGGTCACTCGCCCTCGGACATCGCGTGTACCGACGACTTCT
TAGTCTCAATGAAACACTTCAAAAAATTAATCAACGTC------------
GACAAAGAAAAGCGGTTGTTCACAGCAGAAGGCGGAATAATGATATCAGA
ACTGAACGAATTA---CTTCGTGACCGTGGCTATGCCCTCAGTGTATTGG
GCTCCATTTCGGAATTAACTCTGGCGGGCGTTATATGCACTGGGACTCAT
GGAACAGGCATTCAATTC---GGCAACTTGGCATCTTCTGTTACGGAAAT
CGAATTAATGACGTCTGACGGGGAAATCCGCACGTTGAGTAAAGAGGCA-
--------GACGGCGATGTGTTCAGAGCAGCAGCGGTCAGCCTGGGATGC
CTCGGAGTCATCCTAAAAGTGACCGCTAAGTGCGAAGAGGCGTTCAACTT
GAAACAAAACAGCTATGGCGCCAACATCAAAGACTTGCTGGAAAACCTGG
AC------GTCCATCTCAAGGCATCTGACCACTTCAGGTTCATGTGGTAC
CCGCACACTGACCAGTGCGTTACATTCCACACCAGAAGAACGCAAGAGGC
GGTT----------------------------------------------
-----CGTAGGAGCCACAGCTGGTTCTGGGATTATCTC------------
------------------ATTGGATTCTATCTCCTGGAATTCCTTCTCTG
GATTAGTACC------TGGTTCAAAGGATTTGTG------CCCTTGATCA
ATCGAACCTACAGCCGAATCAAC---------------AGCAAACCCTCC
GAGTTCATTGATGTCAGTTATAAGGTC------TTCAATTTCAACTGTCT
G------TTCCGGCAATATGTTATG---GAGTGG----------------
--------------------------GCCATTCCAATGATAAAAACGCAG
CTCGCTTTGTTTGAATTAAAGAATTGGATC---GGAAATTCA---GGA--
-------------------------------------------TTTGAAG
CTCATTCTCCGGTGGAAGTTCGTTTTGTGCGTGGG---------------
---------------------GATGACATGCTGTTGAGCCCTGCTAATGG
C---CGGGACGTC-------------------------------------
--------------------------------------TGTTATGTGAAC
ATC---------------ATTATA------CCGTACAACAAGTTA-----
-GTTGCTCATGCT---GAGTATTGGGAAGCGTTT---AAGCAGATCATGC
TCCGAAATGACGGCAGACCACATTGGGCAAAGGATCAC------------
------------CTCATGACCGCCAAGGAG--------------------
```

----------------------CTGAGGCCCCTCTAC---CCTAAATGGG
ACACC---TTCTGCCGGATTCGTCAAAAAATGGATCCAAAGGGGATGTTC
ATGAAT------GAGAATTTGAAGAACACCCTTAGCTATTCA--------

>Trichoplax_adhaerens_(Placozoa)

-------------CCTTACGGCAAAATTTTCAGCAACTGGGCTAAAACTT
ATCAGTGCCAGCCAGAGCTATATTTTATT---CCTGAAAGTATCGATCAA
TTAAGAGATGTTATCAAGTTGGCTACAATTCATAAGAAAGTCGTTAGAGT
AGTCGGCTGCGGCCATTCTCCATCGGACATTACGTGCACGTCGGATTACA
TGATCAGTATGGAAAAATTATCCGGTATTCTTGAAGTT------------
GATGTGGATCGTAATCAAATAAAAGTACAAGGTGGTGTCAAGATTGAGCG
GCTACATAAAGTC---CTAGAAGAGTATGGCCTTGCTATTAGCAACATCG
GTTCGGTATCCGATATCACTCTATCGGGAGCAATTGCTACTGGTACTCAC
GCAACAGGTGTCGACTTT---GGCATGCTTGCTACCAATGTCTTAGAGAT
TGAATTATTGCTAGCCAATGGGGAGCTCATGGTATGTTCTCGACATCAA-
--------AACAAGGAAATTTTTTTGTGTGCTTTATGCAATCTTGGTGCA
CTTGGTGTTGTTGTTTCAATCAAATTACAGTGTGAAAAGAGTTTCAAATT
AGAGCAGCAACAATCTTCCTGTACTCTTGACGAGGTATTAGATAATTTGG
AA------AATGAAGTACGCTCATGCGAACACTTTCGTTTTGGTTGGTTT
CCACATACTAATGATGCAGTT---------------------------CC
CAAA--
-----GTCGATAGTTACAATTGGTTTTGGGATAGCATT------------
------------------GTAGGTTATCATTCTCTTCAGTTCTTCTCC--
----------------TACTTTGATAGTCTTATC------CCATGGATAA
ATAGAACGTTCTTTGGATTGTTGTTC------------CGTAAACCAAAG
TATCGTGTCGATGATAGCTATAAGGTA------TTCAACTTTGAATGCTT
G------TTTAAGCAATACGTCACG---GAATGG----------------
--------------------------GCCATACCTAGGGAAAACACTGGC
ATAGTGCTAAGGCAGTTGCGAGATTGGCTG---AACACAAAT---AAC--
---TTTTATG
CTCATTACCCTGTTGAAGTACGATTTGTTAAAAGA---------------
---------------------GATGATGTTTACCTTAGCCAAAATTATGA
A---CGTGATACA-------------------------------------
--------------------------------------TGCCACATCAAC
ATC---------------GTTATGTATAGACCTTATGGAAAATTC-----
-ATTCCTCATGAC---CGCTATTGGAAGGCATAC---GAAGAAATAATGC
TTGCTGCTAAAGGAAAACCCCACTGGGCTAAGGCGCAT------------
------------AGTCTGACGTATAAAGAA--------------------
----------------------TTGTCACAGATGTAT---CCTCAATATG
ATAAG---TTTTGCAAAATCCGCGATGAACTTGATCCCAATCGGATATTT

ATTAAT------CGTTATTTGACCCAAGTTCTCTTTGAAGGCCGGGAAGA
G---

>Achipteria_coleoptrata_(Acari)

----------GGAGTGAAGAATATAAAGTTCACGAATTGGTCGCAAACAT
ATTCTTGTCATCCATTCCTTTATTTTGTT---CCTAAAGATATCGAAGAA
TTGAGAGAAATAATATTTTTGGCCAAAAGTGAGAATAAAAAGATTCGAGT
CGTGGGTTGCGGTCATTCGCCGTCAGATATTTGCTGTACTAATGATTATA
TGATTAGTTTAAGGCTTTTTAATAATGTATTGAATGTG------------
AATCGAGAGAAACATACGGTTAGTGTTGAGACCGGTATTACTTTGACTGA
ACTCAACGCATAT---CTTGACTCTCAAAAGTTGGCGCTTTCGGTTTTGG
GTTCGATTTCTGATATAACTGTTGGGGGAGTAATAAGTACTGGAACTCAT
GGAAGTGGCGGTAAATTT---GGTGTTTTCGCTGATTATGTCTTAGATAT
GGAGTTAATCACCACTTCTGGCGATACGATAAAGTGTTCTCGTGATGAG-
--------AATAATGATGTATTTCTTTCATCACTGTGTGGATTGGGTGCC
ACCGGTATCATAACCCGAGTGACCATTCAATGCGAGCCCGGATTCCTTCT
CTATTCAAACAGCTATCCGTCCACTTTAGATGAAGTGCTCGAAGATTTGG
AC------GACCAAATCAACAGTTGTGATCACTTCAGGCTCTTATGGTTT
CCGCACACGAATTGTGTTTCCGTTTCGAATTATAACCGGATTTACAACAA
ATCG------------------------------GTTACAAAATATTCTA
AATTTCAAATGATTTCTAATTGGGTCTGGAATTACGGC------------
------------------GTCGGTTACTATACCTTAGAGTTCGCGTATTG
GCTCAGCACT------TATTATCAACCATTAGTC------CCGTATATCA
ACAGAATTTGGTTTTGGTTACAGTAT------------TCTCGACCCCAG
GAAGCGATTGATGTCAGCCATAAGATA------TTCAACTTTGAGTGTCT
C------TTCAGACAACATGTGAAC---GAGTGG----------------
--------------------------TCAATACCCAGAGAGAAAACGGCG
AAAGTATTACTGGAACTGAAGGAATGGATT---GAAACGACGCCTAAT--
---ATTTACG
TTCACTTTCCGGTCGAAATTCGTTTCGTCAAAGAG---------------
---------------------GACGACATTTATTTGAGTCCAGCGTATGG
T---CGCGACTCC-------------------------------------
--------------------------------------ACATTCATTAAT
ATA---------------ATTATGTACAGACCTTATGGTAAGGAT-----
-ATCTCGCATTCG---ATATATTGGAATCAATAC---GAAAAGTTGATGA
AAGAGGCCGGGGGTCGACCCCATTGGGCTAAAGCCCAC------------
------------AGAGAGAGTTCTTCTGAT--------------------
----------------------TTCGTTAAAATGTAC---CCGTATTTCA
GGGCC---TGGACTCAAGTCCGTAAACGATTGGATCCGATTAATATGTTA
GTCAAC------TCATATCTCAACAGAATTCTTATCGAA-----------

>Hypochthonius_rufulus_(Acari)

----------------------GTGCACGTGACAAACTGGTCACGCACGT
TCCATTGCGACCCCATTTTGTATTTCATC---CCTAAAGATGTTGAGGAA
CTCAGAGAGATCCTTCTTTTGGCGGAATCAAATCATAAGAAAGTCCGTGT
CATTGGAAATTGTCATTCACCGTCTGACATTTGCTGTACAAATGATTATG
CAATTAATTTGCAACACTTTAATAATGTATTGAGTGTC------------
GATCATGAGACATGCCGTGTCAAAGTCGAGGCCGGAATGATGTTCGTCGA
ACTCATTCAGCAT---TTGGATAAATTGGGAATTGCATTTCCTATTCTCC
CGTCGATATCCGATTTGACCGTTGGTGGTGTCATAAGTACGGGAACACAC
GGTACGGGTATAAATTAT---GGTATAATCTCGACATTTGTGATTGATAT
GGAACTAATCACATCATCGGGTGATACTATAAAATGTTCGCGTAACGAT-
--------AATACCGAAATATTTATGGCGTCACTCTGTGGTTTGGGATCG
ACCGGAATCATAGTCAATGTGACACTTCAATGTGAACCGTCATTTCTGTT
ACATCAGCACATATATCCTTCGACTTTAGATGATGTTCTCGATGATCTGA
CT------GATCAAATACAATCAGCAGATCATTACCGATTCTTATGGTTT
CCGCACACCAATTGTGTGTCGATATGGCAAAACACACGTATTTATAATGC
ACCC------------------------------GTAACTAAATATTCGA
CACTCCAAAAGGCAATAAATTGGTTTTGGGATTATGCA------------
------------------ATGGGGTATTATACCCTCGAATTTGCCTATTG
GTGCAGTACG------TTTTGTCCACGATTAGTC------CCGTACATTA
ATCGTGCAGCATTTTGGTTATTATAC------------TCATCTTCAAAG
GAGAGAATCGAAATCAGTCATAAGGTG------TTTACGTTTGACTGTCT
T------TTTCGACAGTATGTCAAT---GAATGG----------------
--------------------------TCAATACCATTTGAAAATACGGCG
CGCGTTTTACTCGAACTCAAGAATTGGATT---GATTCGACTCCCGAT--
---GTTTACG
CTCATTTTCCGGTCGAAGTTCGCTTCGTTAAGGGT---------------
---------------------GATGATATTTATTTGAGTCCAGCGTTTGG
T---CGCGATTCA-------------------------------------
---------------------------------------ACGTATATCAAT
ATC---------------ATTATGTACAGACCCTATGGTAAGGAT-----
-CAACCGTTTCAA---GAATATTGGAATAAATAC---GAAGAAATTATGA
AAGCGTCCGGAGGACGCCCGCATTGGGCTAAAGCACAT------------
------------CGTGAAACTGCTGCTGAT--------------------
----------------------TTTCTTAAGATGTAT---CCCCACTTTA
AGACA---TGGACTCACGTGAGGAAACGTTTGGACCCGATTAACATGATG
ATGAAT------CCGTATATGGAAAGGATATTTAAGAAAATTTCCAAT--

>Latrodectus_hesperus_(spiders)

---------ATATTAGAACTAGCTAGACAAGAAGAAAAAAAAGTGAAGGT
TGTAGGCTGCCGACATTCTCCATCTGATATAGCTTGTACAAGTGGTATCA
TGATTAGCCTGCTGAATTATAACAAAATTTTAGAAATT------------
GATAAACATAATCGCCGTGTTAAAGTTCAAGCTGGAATAGTTTTAAGTGA
TCTAAATGATGTA---TTGTATTCCAATAACCTTAGTCTCTCAGTGCTTG
GTTCAGTGTCTAATATTACCATTGGTGGGGCAATAAGTGTAGCTACTCAT
GGAACTGGCATTGAATAT---GGTACAATTTCATCTTATGTGACCGAATT
AGATATAATGCTTGCTGATGGTTCAACTAAAACTTTATCTCCTGACAAG-
---------AATGGGATTTATTTCAAGCTGCTCTGTGTAGTCTTGGCTCT
TTTGGAATTATATTAACAGTAACTATTCAATGTGAAGAAGCATTTAATTT
GCAAATGAGACAATATGGATTGAGTCTAAAAGATGTGATTGAAAACTTAG
AT------GTACATTTGTCAGGCTCAGATCATTTTCGATTTATGTGGTTC
CCTTACACAGATAGTGTTGTTGTTTCTCATTCAATAAGAACTGAATTGCC
CCCA---------------------------------ATGAAAATATCAT
GGATTAGTAGGATATGGAATGCAATATATGAATATGGT------------
------------------ATTGGATATCACCTTCTTGAATTTTGCTATTA
TATCAGTACA------TTCTTTCCTCGTATAGTG------CCATATATAA
ATAGATTCTTCTACTATACAACTTTT------------TCGGTGTATACT
AGGAAAATTGATAGGAGCTACAGAATA------TTTAATTTTGAATGTCT
A------TTCAAACAATATGTCAAT---GAATGG----------------
--------------------------GCTATTCCAATAGAGAAAACTGGT
GTAGTTCTATGGCAACTAAGAGAATGGATA---GAATCAACGCCTGAT--
---TTTTATG
TACATTTTCCAATAGAAGTCAGATTTACTAAAGCA---------------
---------------------GACAATATATTTTTAAGTCCAGCACATGG
T---AGAGATACA-------------------------------------
--------------------------------------TGTTATATTAAT
ATT---------------CTTATGTACAGACCCTATGGTAAAGAT-----
-GTACCATATGAA---AAGTATTGGGCAGCATAT---GAAAAAATTATGT
TAGAAGCTGATGGTAGACCACATTGGGCTAAAGCTCAT------------
------------TCAGTGACTGCTGAAATG--------------------
----------------------TTTAGACTGATGTAT---CCATATTTTG
GAAAA---TGGTGCTCAATACGTCACAAGTTGGATCCAACAAATATGTTT
TTTAAT------CCTTATATGGCTAGAATTTTTTCTAACAGCCGAATG--

>Nephila_clavipes_(spiders)

----AAAGACGGAATCCCAAATGTCCACTTCAAAAACTGGGCAGAAACAT
TTGAATGTGTGCCAGAATTGTTTTTTGCT---CCAAAATCTCAAGAAGAA
ATTGCTCAGATATTAGATTTAGCACGAGAAGAAAACAAAAAGGTGAGAGT
TGTGGGCTCTAAACATTCACCTTCTGATATAGCATGTACAAATGGATTTA
TGATCAGTTTGCAGAATTTTAATAAACTGGTTGAGGTT------------
GACAAAAAACTTATGAGAGTAAAAGTGCAAGCTGGTATGAAATTAACTGA
GCTTAATGATATT---TTGTATTCTCATAACCTTGGGCTTTCTGTACTTG
GATCTGTTTCTGATATCACTATCGGAGGAGCAATTAGTGTTGGTACCCAT
GGTACAGGTATCGAATAT---GGGGTCCTTTCATCCTATATAACTGAAAT
GGAACTGATGTTATCTAATAATGCTGTGATAAAAGTCTCTCCTGAAAAA-
--------GATGTCGATGTGTTTCAAGCTGCTGTTTGCAGTCTAGGCTCA
TTTGGGATTATTTTAACTGTTACAATACAATGTGAACAAGCATTCAATTT
GCACATGAGACAATATGGTTTACCTCTAAAAGATGTTGTTGAAAATCTTG
AC------GTACATTTGACAGGATCTGATCATTTTAGATTTATGTGGTTT
CCGTATACAGATAATGTAGTTGTATCTCATACTTCAAGGACCGATTTGCC
TGCC---------------------------------AGAAAAGAAACGT
GGCTTAGAAAAATTTGGAATCTTTTCTGGAATTACGGC------------
------------------ATTGGATATCATGCTCTCGAATTCTGCTATTA
TGTAAGTACA------TTCTTTCCACATTGGGTA------CCACATATCA
ATCGAATCTTCTACTACCTGTTGTTT------------TCAACATCTAGT
ACAAAAACTGATCGAAGCTACAGAGTC------TTTAACTTTGAGTGCCT
T------TTTAAGCAATATGTAAAT---GAATGG----------------
--------------------------GCTATTCCTATTGAAAAAACAGGA
GTTGTATTATGGCAGCTAAGAGAATGGATT---GAAACTACTCCTGAT--
---GTTTATG
TACATTTTCCAATAGAAGTTAGGTTTACAAAAGCA---------------
---------------------GACAACATCTTTATCAGCCCTGCATTTGG
A---AGAGATACT-------------------------------------
--------------------------------------TGTTACATAAAT
ATT---------------ATAATGTACAGGCCGTATGGTAAGTCT-----
-GTTCCATACAAA---ACATATTGGGAGGCATAT---GAACATATTATGT
TGCAGGCTGGTGGTCGTCCTCACTGGCAAAGGCTCAT-------------
------------TCAATTACAGCACAGACA--------------------
----------------------TTTAGACACATGTAT---CCATTATTTG
GAAAA---TGGTGTTCAATACGACAACAGTTAGATCCCATAAATATGTTT
ATGAAT------TCCTATATGACTCGTATATTCTCA--------------


```
--------------------------------------------------
--------------------------------------------------
--------------------------------------------------
--------------------------------------------------
--------------------------------------------------
--------------------------------------------------
--------------------------------------------------
--------------------------------------------------
--------------------------------------------------
--------------------------------------------------
------------
>Platynothrus_peltifer_(Acari)
--------------------------------------------------
--------------------------------------------------
--------------------------------------------------
---------ATCATATTTTTGGCCAAAAGTTGCAATAAAAAAATCCGAAT
CGTGGGCTGCGGTCACTCTCCATCTGATATTTGTTGTACAAATGATTATA
TGATAAGTTTGAGTTATTTTAATAATGTATTGAATGTC------------
AACAGAGAGTTGAATACCATTCAAGTCGAGGCCGGCATTACTTTCAGTGA
ACTCAATGCACAT---CTCGATAGACACAAACTCGCATTTCCTGTATTGG
GTTCGGTCTCAGAATTAACGATCGCAGGAGTGATCAGTACTGGGACTCAC
GGAACGGGTGCCAAATTT---GGTATTATCTCTAGTTATGTAATAGACAT
GGAATTAATAACAACATCTGGAGATACTATTAAGTGTTCTCGGGATGAA-
--------AACAGTGATGTATTTCTATCAGCTCTTTGTGGTCTGGGAGCT
ATTGGTGTGATAATACGAGTGACCCTTCAATGCGAGCCCGCATTCCTTCT
CTGTCAATACAGCTATCCGTCAACTCTAGACGAAGTGCTCGAAGATCTAA
AT------GATCAAATCCAAAGTTGTGATCATTTTCGGTTCTTATGGTTT
CCGCACACCAATTATGTTTCCATTTCAAACAACAGTAGAATTTCTAATAA
AGAG------------------------------ATAACTAAATATTCCA
CATTCCAAACGGCTTATAATTGGTTTTGGAATTACGGT------------
------------------ATCGGATATTATTCGCTTGAATTCGCGTATTG
GGTCAGTACA------TATTGTCAACGATTAGTC------CCATATATTA
ATCGCATCTACTTTTGGCTGCTTTAT------------TCAAAACCTCAA
CAAACAATTGATGTCAGCCATAAAGTC------TTCAATTTTGAATGTCT
T------TTTAAACAACACGTCAAT---GAGTGG----------------
--------------------------TCTATTCCTAATGAAAAGACAGCT
AAAGTATTGAATCAGTTAAAGAGTTGGATA---GAATCGACTCCAGAT--
-------------------------------------------ATTTATG
TTCACTTTCCTGTTGAAGTCCGATTTGTAAAAGAA---------------
---------------------GATGATATTTACTTAAGTCCAGCATTTGG
T---CGCAATTCG-------------------------------------
--------------------------------------ACATTTATTAAC
ATA---------------ATTATGTATAGGCCTTACGGTAAAGAT-----
-GTTTCGCATTCA---GAATATTGGGATCAATAC---GAGAAGATCATGA
AAGAAGCCGGCGGTCGACCTCATTGGGCAAAAGCTCAT------------
-------------AGAGAAACTGCGACTGAT-------------------
----------------------TTCGTAAAGATGTAT---CCATACTTCA
GAGCC---TGGTCTCACGTTAGAAAGCGTTTGGATCCCATCAATATGTTA
GTCAAT------TCATATCTCAACAGAATTTTA-----------------
--------------------------------------------------
--------------------------------------------------
--------------------------------------------------
--------------------------------------------------
--------------------------------------------------
--------------------------------------------------
--------------------------------------------------
--------------------------------------------------
--------------------------------------------------
```


>Rhipicephalus_microplus_(Acari)

-------------ATTGGGCGAATGGAGTGGCATAACTGGTCGGGAAGCG
TGAACGCGCGGCCACAGGCGATCGCCCGG---CCACGCGACGCGGCGCAG
CTTCGCACGGCGATCCTCTCCACG------------CCCAAGGTGCGCGT
GCGCGGCGCGGGTCATTCCTTCATGCCGCTGTGCGAGACGGACGGCACGC
TGATCGACATGAGCGATTATGCCGCCCCGATCGAGATC------------
GCCGCCGACCGCACCACCGCCTGGGTGCCCGCCGGCTGGAGCCTCGCGCG
GCTGACCGAGGCG---CTGTGGGACGAAGGACTGTCGCTGATCAATCAGG
GCGACGTGAACCCGCAGTCGATGGCCGGCGCCACCGCCACCGGCACGCAC
GGCACCGGCAAGGATTTG---GGCAGCCTGTCGACGCAAGTGCTCGCGTT
CGAGCTGATGCTGGCGGACGGCTCGCTCGTCCAGTGCGACGCGACGCAG-
--------AACCCCGATCTGTTTCAGGCGCAGCGCATCTCGCTCGGGCTG
TTCGGCGTCGCCACGCGCATCCGCATCAACGTGCTGCCCGCCTATTATCT
TGAGGAACGGATCGAGGCCCGCCCGCTCGGCGAAATGGCGGAGCGCTGGG
AG------GAACTGGCCGCCGCGACGCGCCATTTCGAATTCTTCGTCTTC
CCCTATGCCGATACGGTGATCTTCAAGAGCCTCCACCCGGCCGAGGGCGA
CGGC--
-----------------ACGCTGCGCCGCTCCACCGAT------------
------------------ATCGACGAGCGCCCGTTCCGCATCGCCTGCGA
GATCAGCCGC------CGTGCGCCCTTCATGATC------CCGTCGCTGC
ATCGGCTGATGATGCGGCTGAGC---------------AGCAAGCCTTCG
CGCCGCATCGGCCCGGCGTGGCAAATC------TTCCCCGGCGAGCGGAC
G------ATCCGCTTCGAAGAAATG---GAATAT----------------
--------------------------GAGCTGCCCCGCGCGGACGGCATG
CCGACGCTGATGGAGGCGATCGGCTATATT---CGCCGCAAG---CGC--
---CTCCCCG
TCGCCTTCCCGTTCGAGTTCCGGCTGGTGGCGGGT---------------
---------------------GACGACATCTGGATGTCGCCGTTCAATCG
CGGCCCCGGCGCG-------------------------------------
--------------------------------------TCC------ATC
TCC---------------------TTCCATCAATATGCGCGG--------
-ATGCCGTGGCGC---GATCTGTTCGCGGAGATC---GAGCCGGTGCTGC
GCGGCGCGAACGGGCGGCCGCATTGGGCAAAGCGCCAC------------
------------ACGCTGGGCGCGGAGGAT--------------------
----------------------GTGCGTGCGCTCTAT---CCCCGGACCG
GCGAT---TTCCTGAAGGTCCGCGCCGGGGTCGATCCGGCGGGGAAGTTC
GTCAAC------GCCGATCTCGCGCGGCTGTTCGATATCGCGGAAGGG--

>Dermatophagoides_farinae_(Acari)

----CGAATAGCAGTGGCTGGATATCAATTTAAAAATTGGTCCGGTACTT
ATCAATGTGAGCCAGAATTGTTTTTTATC---CCAGAAACAATCGATGAA
ATTCGTGAGATTCTCTATTTAGCCAAAACTGAAAAGAAACGAATCCGTGT
AGTTGGATGCGGTAATTCTCCCTCTGATCTATGTTGTTCATCTGATTATA
TGATATCGATGGAACGTTTTTCAAACATTCTTTATATT------------
GATCGACAACAATGCCTTGTCACAGTCCAAGCCGGTATTCATCTTGATCG
ATTGAATAAATTA---CTTGAAGAAAATGAACTAGCGTTGCCTGTTCTAG
GTTCAATATCGGAATCTGCATTGGCTGGAGTCATATCGGTTGGCACACAC
GGAACTGGTTATCAAACG---GGCATCATAGCCGATTATGTGGTAGCTAT
GCGTTTGATAACACCTTCAGGAGACATAATATCTTGTTCAAAAGAATCTG
GCACTCAACATAATGATGTCTTCAATTCTGTACTATGTGGCCTAGGCGCA
TTGGGTATCATATTAGAAGTCACTATTCGATGTGAGCCTAAATTCTTTTT
ACATCAACTAACGTATCCCAGCCGATTAGATTATGTACTTGATCGATTGG
AT------GAGAATGTTGAATCATGTGATCATTTCCGTTTTCTTTACTTT
CCACATACCGATTATGTTTCTGTTTCCATAACGAACAAAGTGAAAGAACG
TGAT---A
TGCTTCAGAAAATTTTCGACTGGATCATTAAATATGGT------------
------------------GTTGGCTATCATCTACTTCAATTCGCTTATTG
GCTATCAACT------TATGCGCCGGCAATAGTG------CCCACCATCA
ATCGATTAGCATTCTGGCTGCTTTAT------------TCATCAAATCAA
GTCCAAATGGACATTAGTTATAAAGTT------TTTAATTTCGAATGCCT
G------TTCGCTCAACACGTCAAC---GAATGG----------------
--------------------------TCCATTCCCAGGAACAAGACAAGT
CAAGTGTTACGCGAGCTAAAAGCAGCAATC---GATCATGGT---GGC--
---CTATACG
CACATTTTCCTATCGAAGTTCGATTTGTTCAACCA---------------
---------------------TCTTCCATCTATCTTGCGCCAAGTTATGG
T---CGTGATTCA-------------------------------------
--------------------------------------ACATACATTAAC
ATT---------------ATCTCATATCGACCATATGGACGACAT-----
-GTTGACCATACC---GATTATTGGAATCGTTAT---GAAGAGATTATGA
AACGAAACGGAGGACGGCCTCATTGGGCCAAGAATCAT------------
------------CGAGAAACGGCCGTTGAT--------------------
----------------------TTGGTTCGAATGTAT---CCGCATTTCC
GATAT---TGGGCTCATATACGCAAAAGATTGGATCCAAATCGACTATTT
TTCAAT------TCATATCTTGATCGAATCTTTTCAACATTTCCGACTAG
CACAATAGCCACCAGTGACCATAATGAATCT---------AAAATGTTTG
CTAACGTCCAAGGTGGTGATGGTAGTTTGGATTTGTCTCAATCTTTACAC

>Radix_auricularia_(Gastropoda)

----TCTCTTGGAAAGTCTGGTAAAAAGTTTACTAACTGGGCCACTACCT
ATTCTTGCACTCCGGAACTTTATTTTGAA---CCAGAAACAGAGGATGAC
ATTAAAAAGATTTTGGCCTATGCTCAAGAAAAAGACAAAAAAGTCAAGGT
CGTTGGTTTTGGCCACTCACCTTCTGATTTAGCCTGTACGTCTGATTATA
TGATTAGTCTCTGCAGGTTCAACAAGGTCATAAAGGTT------------
GACAAAGAAAAATTGCAAGTAACTGTTCAAGGTGGCTGTCTTCTTAAAGA
ACTGAATGATCACGTCCTACCAGCTAATGGGATGGCACTCTCTGTACAAG
GGGCTGTATGTGATTTGACTGCAGCTGGTGTTATTAGTACCGGCACTCAC
GGTACAGGTGCAGAGTTT---GGTATCATTTCATCTTATGTTGTTGGTCT
TGAGCTAATGAAAGCTTCAGGGGAAGTAATTACCATCAACCAAGAACTC-
--------AACTCAGAATTACTTCCAGCTGTTTCTCTTAGTCTGGGCTCC
TTGGGTATCATTCTATCTGTCACATTGCAGTGTGAGAAAGCTTTCAGGCT
GCATTGCAAACAAGTGACAAACAACTTGCAAAATGTGATTGAAAACCTTG
AT------GTGTATGTGACCAGTTCTGATCATTTCAAGTTTTTCTGGTAT
CCACACACAGAATCTGTCGTTTGCTTTTTCACAAATAGAACAAAAGAGGA
ACCT--
-----AATGTAAAGTCAAGCTGGTTCTGGGAGATGGGA------------
------------------GTTGGTTACTATTTACTACAGATATTG-----
-TTATGTACT------TTTTTCCCCACTTTAGTT------CCTGGCATCA
ACAGACTTTACTTAAAGCTCTTCTGT------------TCTAGAAGTGTA
GAGAGAGTAGACAGAAGTGATAAGATC------TTTAACTTTAATTGTTT
G------TTCAAGCAGTACGTCATG---GAATGG----------------
--------------------------TCTATACCCAGGTCCAAAGTTGGA
GTAGTTCTGTGGGAACTGAAAGAGTGGATT---GAGAAAAAC---AAA--
---TTTCCGG
CACATTTTCCCGTGGAAGTGCGTTTTGTCAAGGGA---------------
---------------------GATAATATTTATCTGAGTCCTTGCTATAA
A---CAAGATTCC-------------------------------------
--------------------------------------TGCTATATTAAC
ATT---------------ATTATGTACAGGCCATTCAATAAACTT-----
-GTGTCACATGAA---ACCTACTGGAATGAGTAT---CAAAGTATAGTTG
CCAAACATGGCGGAAGGCCACACTGGGCTAAGGACCAT------------
------------AACTTTACAGGCACAGAA--------------------
----------------------TTCATCAAGCTATAT---CCAAAGTGGA
ATGAG---TTTTGTGCCATCCGTGAAAAAATGGACCCTAATGGGATGTTC
CTCAAT------GCAAATCTGGAGAGAGTATTTAACAACAGACCTTCACC
TTCAAGGAATCATGTA----------------------------------


```
--------------------------------------------------
--------------------------------------------------
------------
>Aplysia_californica_(California_sea_hare)_Aplysiidae_XP_005103891.1_(Gas
tropoda)
---------------------------------ATGTCAAATTTTGATAT
TCGGACCCTCGGCAAGTCAGGTAGGCAGTTCACCAACTGGGCAACTACAT
ACTCATGTCAGCCAGAACTGTATTTTGAA---CCTGAGAATAATGAAGAA
ATAAAGAATATTGTTGATTATGCACGAGAGAAGGGGAAAAAGGTGAAAGT
GGTTGGTTTTGGACATTCCCCTTCAGACATCGCTTGTACTACAGACTTTA
TGATCAGCCTGAAGCGCTACAATAAAGTTTTGAAGGTT------------
GATGAAGAGAAGTCTCAGGTGAGCGTGCAAGCTGGATGCTTGATAAAAGA
CCTCAACCACAACGTCCTTCCAGCGCACAACTTGGCATTCTCTGTCCAAG
GGACTGTGGACGATCTCTCAGCTGCTGGTGTCATTAGCACTGGAACTCAC
GGGACTGGCACCAACTTT---GGCATTGTGTCTTCCTATGTCGTGGATCT
CACTCTAATGAAAGCCTCTGGGGAGATAATCACCATCAGCAAAGAAGAA-
--------AACTCTGAGTTGTTCAACGCTGCTGTTCTGGGTCTTGGCTCG
CTTGGAGTGATTCTCTCTGTCACGTTCCAGTGCGAGAAAGCTTACAGACT
TCACCAGAGGCAGTTCCCCAGCTCCCTGAAAAATGTCATTGACAACTTTG
AC------AAGCATGTGGCAGAGGCTGACCATTTCCGCTTCATGTGGTAT
CCTCACACTGAGTCTGTCGTCTGTTACAGTGCCAGCAGGACCAAAAAGGA
GCCA----------------------------------------------
-----GCCGTGAAGTCCAGCTGGTTTTGGGATATGTTC------------
------------------GTTGGCTACCATCTCTTACAACTTTTGCTGTG
GATAAGCACG------TTTGTGCCTTGTCTGGTG------AAGAGCATCA
ACCGTCTGTACTTTCGCCTCATGTGT------------CAGCAGCCCATC
GAGAAGGTGGCCCGCAGCGACCGGGTC------TTTAACTTCAACTGCCT
G------TTCAAGCAGTATGTCACA---GAGTGG----------------
--------------------------GCCATACCCAAAGAGAACACGGGC
GTGGTGCTGTACAAGCTGAAAGACTGGATT---GAGCAGAAC---GGT--
-------------------------------------------TTCTACG
CACACTTCCCCGTGGAGGTTCGCTTTGTGCGTGCT---------------
---------------------GATGACATCTACCTGAGCCCATGTTATCA
A---CAAGACTCC-------------------------------------
--------------------------------------TGCTATATCAAC
ATT---------------ATCATGTACAGGCCCTTCAACAAGCTG-----
-GTGGACCACGAG---TCGTACTGGACGGCGTAC---GAAAGCATCATGG
CTGAGCATGGAGGAAGACCACACTGGGCCAAGGCACAC------------
------------AAGTACACAGGATTGCAG--------------------
----------------------TTCCAGAAAATTTAC---CCCAAGTGGA
AAGAG---TTTTGTGCCATCCGGGAAAAGCTGGACCCTAACGGAATGTTC
CTCAAC------GCCAACTTGGAGCGCGTGTTTGATGCCAGACCTTCTCC
TTCCACCTACGTC-------------------------------------
--------------------------------------------------
--------------------------------------------------
--------------------------------------------------
--------------------------------------------------
--------------------------------------------------
--------------------------------------------------
--------------------------------------------------
--------------------------------------------------
--------------------------------------------------
--------------------------------------------------
--------------------------------------------------
--------------------------------------------------
--------------------------------------------------
--------------------------------------------------
--------------------------------------------------
```


>Lottia_gigantea_(owl_limpet)_Lottiidae_ESO97787.1_(Gastropoda)
------------------------------------ATGACATTAGACAT
ACGGTCTTTAGGTAAACCGGGATATAAATTCACGAATTGGGCTACAACAT
ATTCATGTTACCCAGAACTTTACTTCGAG---CCAGAAACTACAGACGAA
ATTCGACAGATATTGGCGTTTGCCCAGAAGAAGGGCAAGAAAGTGAAAGT
TGTGGGGAATGGACATTCACCATCAGATTTGGCCTGTACTACAGACTTTA
TGATCAGTTTAAAGCGATATAACAGAGTTCTACAGGTC------------
GATAAAGAATCAAGATTAGTACGTGTTCAAGGTGGCTGTCTAATAAAAGA
TTTAAATGAAAGGATTTTACCGGCCAATGGTTTGGCTTTCACTGTGCAGG
GAACCGTTAGTGATTTAACTGCCGCTGGTGTCATTAGTACAGGCACTCAT
GGTACTGGACATGATTTC---GGGATCATTTCAACATTTGTCCGAGAGAT
CGAACTGATGCAAGCATCTGGAGAAGTATTAACAATATCCAGGGATTTA-
-----CAGAACACGGATCTATTTCCAGCAGCCTGTGTCTCATTAGGTGCA
CTTGGTGTTATATTGAGTGTTACATTTAAGTGTGAAGCAGCCTTCAATCT
GGAACAGACACAATTCCCCAATTCCCTGAACAATGTATTAGAGAATTTAG
AC------GAATATGTTACTGGATCTGACCATTTCCGCTTCTTCTATTTC
CCATATACCAACAGCACACTGTGCTTTAACGCTAATAGAACCTCTAAGCC
TAAG--
-----AATGTAAAATCAAGTTGGTTTTGGGAAACTTTA------------
------------------GTTGGTTATCACATTTTTCAATTTTTCTATTG
GCTAGGTGCC------TTTATAACTGCCTTAGTA------CCTGTAGTAA
ATCGATTGTTGTACAGGGTGATATAC------------GCACACAAGTCA
GAAAAAGTCGATAGATCTGATCATATA------TTTAATTTTAATTGTTT
A------TTTAAACAGTATGTTTCA---GAATGG----------------
--------------------------TCCATACCAAGAGAGAAGACATCT
TACGTTTTAAAAGAAATTCAGAAATGGATT---ATAGACAAC---AAT--
---TTCCCTG
CTCATGTTCCAATAGAAGTGAGATTTGTGAAAGGG---------------
---------------------GATGATCTTTATTTAAGCCCAGCTTATGG
ATGGAAGGATACA-------------------------------------
--------------------------------------TGTTACATCAAT
ATA---------------ATTATGTATACACCTTTTAATAAGATT-----
-GTACCACGAGAG---AAATATTGGAAAGCCTTT---GAAAAAATTATGT
CCGATGTTGGTGGACGACCTCATTGGGCAAAGGATCAT------------
------------AAATATGGACGAGAGGAG--------------------
----------------------TTTCAGAACCTGTAC---CCTCAGTTTG
ATACC---TTCTGTAAAATCAGAGAACGTCTGGATCCTAATGGAATGTTC
CTGAAC------TCCAACCTGGAACGAGTCTTTGGGAACAGTTCGAATTC
CATCTTTAAAGTG-------------------------------------

>Colubraria_reticulata_1_(Gastropoda)

-------CACGGGATACCAGGACACCAGTTCACCAACTGGAATGACACGT
ACTCCTGCCAGCCCGAGTTGTTCTTTGAA---CCCGACACCGTCCAACAG
CTGCAGCAGATTCTGGAAGCTGCTCGGAGGGCAGGGAAGAAAGTAAGGGT
TGTGGGCATGGGACACTCCTTCCACGATCTGCCCTGCACCACGGAGTACA
TGATTTCTCTCAGTCGCTACACCAAGGTCCTAAAGGTG------------
GATGAGGGCCTGCAGCAGGTGACAGTACAGGCAGGTCTGGTGCTCAAGGA
CCTGGTTAATGACGTGCTGCCCCCTCATGGACTGGCTCTTGGCGTGACAG
GAGCCATGCCACGGATCACAGTGGGAGGTTTCATGTCTACTGGCACACAT
GGCAGCGGAATGAAGTAT---GGCACCATGTCTTCTCTGGTGGTTTCCCT
TGACCTGCTTACGGCATCAGGGGAGATCATGCACATCTCCAAGGAGGAG-
--------AACTCTGATGTGCTGGAAGCTGCGCTGTTGAGCCTGGGGACG
CTAGGCGTGGTGCTGACGGTCACGCTGCAGTGTGAGTCTGCCTTCACCGT
CCGGCAACGCCAGTACCCTGCCACGCTTGACTACGTGTTGGACAATCTAC
CT------GAGCTGCTGGACGCCAGTGACTATTTCCAGTTCTGTTATTTC
CCGCATACAGACGGTGCAGTCTGCTACCAGGCAACTCGCACAAAAGAGGA
ACCC--
-----AACGATGTGAGCAGCTGGTTCTGGGACGTGTTC------------
------------------ATTGGGATCCACGTCTTGCAGCTTCTGCTCTG
GATATGCACA------TTCATTCCTCGGCTGACG------CCGTTGCTCA
ACAATCTGTTTTACCGCTTCGTGTTC------------CATCGGTCAGTG
GACGTAGTGGACAGTGCTCATCGCGTG------CTGTGTCACGACTGCCC
C------ATCCCTCTTAATGCCTGT---GAATGG----------------
--------------------------GCTGTGGCCAAAGAGCACACAGGC
CCCGCACTGAGAGAGCTGCAGCGATGGGTC---ACCCAAAGT---GGA--
---GGCCCTG
CCACGTTTCCTGTGGAGGTACGCTTTGTGAAGGGA---------------
---------------------GAGACCATCTACCTGAGCCCTGCCCACGG
A---AGGGACACC-------------------------------------
--------------------------------------TGCTACTTCAGC
ATC---------------TTCTACTACAGACCTTACAACAAAGAT-----
-GTACATAACGAC---AAGTACTGGGACAGCTTT---CAGCGAATCATGG
CTTCCTTCGGTGGACGACCTCACTGGGCCAAGGACCAC------------
------------AAGTACGGGTGCCAGGAC--------------------
----------------------TTTGAGAAGGTGTAC---CCCAAGTGGG
GCGAG---TTCTGCGCCATCCGGAAGCGGATGGACCCCTCCGGCATGTTC
CTCAAT------GATCACCTGCAGCGTGTCTTCCTCAGCAAGCCCTCCCC
CGTGGCCACTACGACGGCTGTT----------------------------

>Colubraria_reticulata_2_(Gastropoda)

--------------------------------GTGCTGAGCCTGGGGGCG
CTGGGAGTGGTTCTGACGGTCACCATGCAGTGTCAGCCTGCCTTCACCCT
GCGACAACACCAGTACCCCGCCAAGCTGAAAGACGTGATAGAGAATGTGA
AT------GTGTATGTGACAGCCAGTGATCATTTTCGGTTCTTCTGGTTC
CCACACACGGACGACGTCATCTGCTATCATGCTTCTCGTACCAAACAGGA
GCCC---A
TGCTGCAAAAATCAAGCGGCTGGTTCTGGGACACGTTT------------
------------------GTGGGATTCTATGTTTTGCAGCTGGTGTATTG
GATCAGCACG------TTGATTCCTAGTCTGGTG------CCGGCCATCA
ACCGGTTTTACTACCGCGTGTTGTAC------------GCCCACAGCGTG
GAGAGGGTCGATGTCAGCCATAAGATC------TTCAGCTTCAACTGTCT
C------TTCCGCCAGTACGTCACG---GAGTGG----------------
--------------------------GCCATTCCACGAGAGAAAGTGGGG
GTCGTTCTGGTGGAACTGAGCAGCTGGATC---TCCAACAAC---AGC--
---TTCCCGG
CACATTTCCCAGTGGAGGTGCGCTTCGTCAAAGAG---------------
---------------------GATGACATCTACCTCAGCCCCGCCTATGG
C---AGAGAGTCC-------------------------------------
--------------------------------------TGCTACATCAAT
ATC---------------GTCATGTACAGACCGTTCAACAAGTAC-----
-ATCCCCAATCAG---AAGTACTGGGACGCCTTT---GAACGCATTATGT
TGTCAGCAGGCGGGCGACCCCATTGGGCCAAGGACCAC------------
------------AATCTGAGGGGGCAGGAC--------------------
----------------------TTCGAGAGGCTGTAT---CCAAAGTGGG
GGGAG---TTCTGCCGACTGCGGGAACAGCTGGACCCCAACGGGATGTTC
CTCAAC------GCCAACCTGGAACGCGTGTTT-----------------

>Lymnaea_stagnalis_(Gastropoda)

----TCCCTTGGAAAGTCTGGTAAGAAATTTACCAACTGGGCCACAACAT
ATTCTTGCACTCCAGAGCTTTATTTTGAG---CCAGAAACAGAGGATGAG

ATTAGAAAGATTTTAGCCTATGCTCAAGAAAAAGACAAGAAGGTCAAGGT
TGTTGGTTTTGGCCACTCACCTTCTGATATAGCCTGTACAACTGATTTTA
TGATAAGTCTCTGCAGGTACAACAATGTCATAAAGGTT------------
GACAAAGAAAAATTGCAGGTGACAGTTCAAGGTGGCTGCCTTCTTAAAGA
ACTGAATGATCATGTCCTACCAGCCAATGACATGGCACTCTCTGTGCAAG
GGGCTGTATGTGATTTGACTGCTGCTGGAGTTATCAGCACAGGGACTCAT
GGGACAGGAGCACAGTTC---GGCATAATTTCATCTTACGTTGTTAATCT
TGAGCTAATGAAAGCTTCAGGGGAAGTGATTACTATAAACGAAGAGGTC-
--------AACTCAGAATTGCTACCAGCTGCTGCTCTCAGTCTGGGCAGC
TTGGGTGTAATTATCTCTGTCACATTGCAATGTGAGAAAGCCTTTAGGTT
GCATTGTAAACAAGTCACAAACACCTTGCAAAATGTAATAGAAAACCTTG
AT------GTCTATGTAACCAGCTCTGACCATTTCAAATTCTTCTGGTAT
CCACATACAGAATCTGTTGTTTGCTTCAACACTAATCGAACAAAAGAGGA
ACCC--
-----AAAGTAAAGTCCAGCTGGATCTGGGATATGGGA------------
------------------GTTGGCTACTATTTACTACAAATATTC-----
-------ACT------TTTTTCCCAAGTTTAGTC------CCTAGTATCA
ACAGACTGTACTTCAAGCTCTTCTGT------------TCTAGCTCTGTG
GAAAGAATAGACAGAAGTGATAAGATC------TTTAACTTCAACTGTTT
A------TTCAAGCAATATGTCATG---GAGTGG----------------
--------------------------TCTATACCCAGGAACAAAGTTGGA
GTTGTTCTTTGGGAGTTGAAGGAGTGGATT---GAGAAAAAT---AGA--
---TTTCCTG
CACATTTTCCTGTGGAAGTTCGTTTCGTCAAGGGA---------------
---------------------GACAATATTTATCTGAGCCCTTGCTATGA
T---CAAGATTCT-------------------------------------
--------------------------------------TGCTATATAAAC
ATT---------------ATTATGTACAGGCCATTTAATACATTG-----
-GTGTCACATGAA---ACCTATTGGAATGAGTAT---CAAAGTATAGTTG
CCAAACATGGGGGAAGGCCACACTGGGCTAAGGACCAT------------
------------AATTTCACAGGCACAGAA--------------------
----------------------TTTATCAAGCTATAT---CCAAAGTGGA
ATGAG---TTTTGTGCTATCCGTGAGAAGATGGACCCTAATGGTATGTTT
CTCAAT------GCAAATCTGGAGAGAGTATTCAACAGTCGGCCTTCACC
TTCAAGGAATCATGTG----------------------------------

>Kudoa_iwatai_(Myxozoa)

----------------ATTGATTATATATATCGAAATTGGGCTGGTACCT
ATTTCTGCAAACCCCAATTATATTTTCAA---CCTGAAACTATCGATCAA
ATATTAGAAATATATGATCTTGCGAAACTATATGGAAAGAATATAAGGTT
TATAGGAAGTTCAGCTTCTCCATCCAATCTACCGTGTACTAATGGATATA

TGGTTTGTATGAAAAAATTTAATAAGATTCTATGTATT------------
GATAAAGAAAAGTTAGAGGTAAAAGTACAAGGGGGTATTATATTATCTGA
ATTACAGGAAATA---CTCTATAGAAATGGCTTGGCTATATCTACTTTAC
CATCAATCAATTTTATAACACTTGCAGGAGCAATATCAACAGGAACTCAT
GGAAATGGACTTAAACAT---GGCAATCTTTCATCAATGGTTTTAAGCAT
AGAGATTTTATCTCCAAATGGACAACTTTTGTATTTGGATTCTAACAAC-
--------AATAAAGAGTTATTTCTTGCTGCTAAAGTTTCCCTTGGTCTT
CTAGGTGCTATTATTAATGTTACACTACATGTTGAACACTGTTTTAATGT
AATCGAAAAAAAATATCCATGCACTTTTGATTATATGATCAATAACATTG
AT------AATATAATAAATGCTAGTGAATTCAGTAAAATAATGTTCTAC
CCTCACACAGATAAATGTGTTATATTTGAAATGGATAGAACAGATAGAAA
AGAA---T
CTCCATCATCATATATCAGTATTTTATGG---------------------
------------------------ATAAAATTATTGGAAAAGTCTTGTTT
TATTACCTGC---TGTCGTATACCCTCCATAACT------AAGTACATTA
ATAATCTTTTCTTTTACATCCTCCAT------------AATAAAATTAGT
GAACGTATAGACAGGAGCGATTGTATA------TTTAATTTTGAAGTGTA
T------TTTCCCCAGTATGTAACA---GAAATT----------------
--------------------------TCTTTTAAAATTGAGGATACTAAA
GAGATTTTGGAAGAATTTTATAATCTTATA---AAGCAGCAC---AAA--
---CTTAATG
TGCATTTTCCTGTTGAAATACGATTTGTAAAAGCA---------------
---------------------GATGATGCTTATCTCAGCCAAAATAATGG
T---TACGACTGT-------------------------------------
--------------------------------------ATGATGATAAAC
TTA---------------TTGGCCTTCCGACCAGGACAATCCCAT-----
-TTTCCTAAAGAA---GAATATTTCACACTTTTTGATGAAAAGATACGTA
AAAAATTCAATGGTAGATCTCATTGGGCTAAGACGCAT------------
------------ACTGATAATCCAGCATCT--------------------
----------------------CTACAAACCTTATAT---CCAGATTTTG
GAAAA---TTTTTGACAGCAAGAGAGTTCCTTGATCCAGATGGTGTTTTG
CTTAAC------GATTATGCTAAAATACACCTTTTTGGTAAAAAAATTAA
A---

>Hyalella_azteca_(Crustacea)_1

----------------------------TGGAAAAACTGGTCTGAGCTAC
ATAAAGCAAACCCAAAAGTAATCGCGGCT---CCAAAGGACGAGGCAGAG
CTTGTCAAACTGATGAAAGAAGCC-------------AAAACGGTTCGTTT
CGCTGGAACCTCACACTCGTTTATGCCTCTGATTCCAAATGATGACACTA
TCATTTCATTAGACAACTTCAAAGGAGTTCTCTCTGTG------------
GACAAAGAAAAGGTACAAGCCAGAGTGGGTGCAGGAACCAAACTCTTTTT

TCTATCCAGTGAA---TTAGATAAACATGGACAAGCCTTGGCCAGTATTC
CAGACATCAACACCCAAAGTTTTGCGGGTGCTATGTCAACGGGAACCCAT
GGAACAGGAAAAAAATGG---CAAGCCATGCACGCCTATGTAAAAGCCAT
CAAAATTGTGGCTCCAACTGGGGTTGTATATGAATGCTCCGAAACCCAA-
--------AACCCAGACATTTTCCAAGCAGCCAAAGTATCTGTGGGAACC
CTTGGGGCCATTACAGAGGTAACGCTCCAAAACAGACCTGCCTACAACGT
GCACAAAACGGTTAAAGTACAAGAGTTAGATTGGGTTTGGAAAAATGCAC
AA------AATTCTTTCGAAACCGCTGAACACTTTGAAATCTATTACATC
CCTCATACCAACTTGGTGGCACTATTGACCTCTGAAATACATGAAGGAGA
AGTA---C
AAGACAGACCCAATTCTGAGGATGAAAACGCACTCAAA------------
------------------GATTTGAAAATGCTCCGTGATTACTTTTCATG
GAGTCCATGG------CTGAGAAAAAAACTCGCT------TCTACCATGG
GAAAACCAGAC---------------------------GATGTAATAGAG
GATTTCAAAGACAAATCTTTTAAATCT------TTGGCTCAACCCCGTGT
G------ACCAAGTTCAACGAATCT---GAATAC----------------
--------------------------CACATTCCCAAAGAAAATGCTTTT
GCTTGTTTCAAAGAAGTGGTAGAACTTATC---GATAGCATGCCTGGA--
---ACTTAC-
-----TATCCAGTGGAGCTTAGAAACATCAAACAA---------------
---------------------GATGATGCTTGGCTCAGTCCATTTTACAT
G---AAAGATTGT-------------------------------------
--------------------------------------TATTCTATTGCC
GTG------------------------------CATGCGGACAAT-----
-ACAGAAGAATATCAGTATTTGCTAGATGATTTT---GGTAAAATCTTCA
GAAAACACGGTGGTAGACCTCACTGGGGAAAACTCAAT------------
------------GACTTCACAAAAGAAGAT--------------------
----------------------TTTGCAGCGGCCTAC---CCAAAATGGA
ACGAC---TTTTTGGCTGTAAGACAAAAATTGGATCCAGAAGGAAAATTG
TTAAAC------GAGTATACAAGAAAAATTTTCGGA--------------

>Hyalella_azteca_(Crustacea)_2

----------------------GCCAAATGGTACAACTGGTCCAAAAGTT
TGGAGGCCAACCCCAAATATATCCAAGTA---CCCAAGGATGAAAAAGAA
CTCATCTCTGTGATGCAAAAGGCT------------AGTCAGGTAAGATT
TATGGGAACTAGCCATTCTTATATGCCACTTGTTCCCACAGAAGATACCA
TGGTTTCTCTTGCCTATTTTAATCAGGTTTCCAAAGCA------------
---GAGGAACCTCTTATGGCCTATGTTGGGCCTGGAATTGTGCTTTCTTC
CATGAACCGAAAA---TTGGAGGAATTCGGTCAAGATTATATCAGCATGC
CGGGAGTCAACCAACAGACTTTTGGGGGAGCCATGTCCACTGGAACCCAT

```
GGAACGGGAAAGGATTTG---ATGGCTTTACACGCTTACATCAAACAAAT
CAAACTCATCACAGCAAATGGTGAAACGGTGGTGTAGCAAAGACAAA-
--------GAATCTGAGATTTTTGAATCTGCCAAAGTTTCGCTGGGACTT
CTTGGTGCCATCACCGAAGTAAAAGTACAAAACAGAGAGAGCTTCAACAT
GAAACAAACCATCAAGATGGAGACACTGGAATGGTTCTTGGAAAATGGAG
AA------AAACTTTTTGAAACCACAGAATGTTTTGAGTGTTTGTACATT
CCAAATACGGGTAAAGTGGTGATGATTTCTTCCCAACAGTGGGATGGAGA
AATT---------------------------------------------C
ATCCCAAAACCCATTCCAACGATGAAGAAAACATTCAA------------
------------------ACTTTAAAATTGCTCAGAGATACCCTGTCTTG
GAGTCCATAT------CTTAGAAGAAAACTCTTT------GATTGGTTTA
CTTCAATGAAC---------------------------AATGTAGAAGAA
GAACTCTATGATAGGTCCTTCCGAATT------TTGTGCCACCACATGGT
T------ACCAAAGTCAATGAGTCC---GAATAC----------------
---------------------------CACGTTCCTCTTGAGAATGGATT
TCTTGTTTTGGAGAAGTTTGTGCACTGATG---GATACTTTTCCTACT--
-------------------------------------------GCCTTT-
-----TTCTTTTTGGAAATCAGAAGAGTCAAAGGG----------------
---------------------GACGATGCCTACATGAGTCCTTTTTACCA
A---AGAGATTCG-------------------------------------
--------------------------------------TACAGTGTATCG
GTG----------------------CATTCTCCTGTGGAGAAAAT-----
-CCAATTGACCTT------------ACTCCATTT---GGTGCCATTTACA
GAAAACACAAGGGAAGACCACACTGGGGAAAATGGCAT------------
------------GACTATTCAAGAGAAGAA--------------------
----------------------ATTGAGGCAGCCTAC---CCAAAAATGC
AGAAT---TTCTTGGATGTTAGAAAAATGTTGGACCCAAAGGGCAAGCTG
TTAAAT------GATTATACCAGAAAGATGTTTGTT--------------
--------------------------------------------------
--------------------------------------------------
--------------------------------------------------
--------------------------------------------------
--------------------------------------------------
--------------------------------------------------
--------------------------------------------------
--------------------------------------------------
--------------------------------------------------
--------------------------------------------------
--------------------------------------------------
--------------------------------------------------
--------------------------------------------------
--------------------------------------------------
--------------------------------------------------
--------------------------------------------------
--------------------------------------------------
------------
>Steganacarus_magnus_(Acari)_partial
--------------------------------------------------
--------------------------------------------------
--------------------------------------------------
--------------------------------------------------
--------------------------------------------------
--------------------------------------------------
--------------------------------------------------
----------------------------------------------TTAG
GTTCGATATCAGAGCTTACAATTGGCGGAACAATTAGTACTGGAACTCAC
GGAACGGGCGCTAACTTT---GGCATAATGTCTTCATACGTGATCGATGT
TGAAATGATTACTTCTTCCGGAGATACCATCAAATGTTCGCGCGACGAA-
```

```
--------AACAGTGATATATTTTTGTCAGCGATGTGTGGTTTGGGTGCT
ATCGGTATAATAACGAAAGTGTCGTTGCAATGCGAGCCATCATTCCTTCT
TCATCAGTACATATATCCATCCACATTAGATGACGTTCTCACTGATTTGA
AT------GATCAGATCAGGAGTTGTGATCACTATAGGTTTCTATGGTTT
CCTCATACGAACTGTGTGTCAATTTGGCAAAACAGTAGAATTTATAACAA
ATCA------------------------------ATTACTAAATACTCTA
CATTTCAACAAATGTATAATTGGTTCTGGGATTACGGC------------
------------------GTCGGTTATTATACGCTAGAGTTTTGGTATTG
GATGAGTACT------TTTGTGCCATCATTAGTG------CCATATATTA
ACCGAATCGCGTTTTGGTTGCTTTAT------------TCCAAACCAAAG
GAAACTGTGGATATAAGTCACAAAATA------TTTAATTTCGAGTGTCT
T------TTTAAGCAACACGTCAAT---GAATGG----------------
--------------------------TCGATTCCTGTTGAAAAGACGGCT
AAAGTATTGCTAGAGTTAAAGCAATGGATT---GACACGACGCCTAAT--
-------------------------------------------GTTTACG
CGCACTTTCCAGTTGAGGTACGTTTCGTAAAGGCT---------------
---------------------GATGACATTTATTTAAGTCCAGCGTATGG
C---CGCGATTCT-------------------------------------
--------------------------------------ACTTTTATTAAC
ATC---------------ATTATGTATCGACCGTTTGGCAAAGAT-----
-GTTCACTATCAG---GATTATTGGAACCAATAT---GAAAAGATTATGA
AAGAATCTGGTGGTCGACCACATTGGGCAAAAGCTCAT------------
------------AGAGAAACTGCAGCTGAT--------------------
----------------------TTTGTTAAGATGTAT---CCGTATTTCA
GAGCG---TGGATACACGTTAGAAAAAGAGTTGATCCCATGAATATGTTT
ATGAAT------CAATATCTTGAGAGAATTTTTTTAAAATACCCGATTGA
TGGACATAAT----------------------------------------
--------------------------------------------------
--------------------------------------------------
--------------------------------------------------
--------------------------------------------------
--------------------------------------------------
--------------------------------------------------
--------------------------------------------------
--------------------------------------------------
--------------------------------------------------
--------------------------------------------------
--------------------------------------------------
--------------------------------------------------
--------------------------------------------------
--------------------------------------------------
--------------------------------------------------
--------------------------------------------------
------------
>Ixodes_scapularis_(Acari)_partial
--------------------------------------------------
----ATTTCGGCTATCAAAGGGGTGACGTTCGAAAACTGGTCTAAAACGT
TTTCCTGCAAGCCCGAGTACTTGTTTGCT---CCCAGAAGCAAAGATGAA
TTATGTGAGGTGCTGGAGTTTGCCCTCCAACGTAGCATGAAGGTCCGCGT
GGTTGGTGCTGGTTATTCCCCGTCAGACATTGCATGCACCTCGGATGTGA
TGATCAGCATGCGCAGCATGGACAAGGTGCTTGAGGTC------------
AACAAAGAAAAGATGACTGTCAAGGCGGAAGCTGGGGTCACTCTGAAAAG
GCTGAACGAAGTCGAACTGGCCAAGAACGGACTCGCCCTT------CTGG
GTGCCGTGTCGGAGATCACGCTTGGCGGTGCAATCGCCACCGGCACTCAC
GGATCGGGACTCAATTTT---GGAATCCTGTCAACGCAGGTGTTGGAGCT
GGAACTGGTGACTTGTCTGGGCAAGACGCTGGTCTGCTCGCGGGAGAGC-
--------AACCCGGAGGTCTTCCTGGCAGCCGCCTGCGGGCTCGGGGCC
ATCGGGGTGGTCGTCGCGGTCACCGTGCAGTGCGAGCCAGCCTTTCGGCT
```

GCGCGAGTCCCGCTACTCCTGCGCCTTGCAGCGGGTGCTGGAAAACCTGG
AC------GTGCACCTGAAGAGCTCAGACCACTTTCGCTGCCTGTGGTAT
CCGCACACGGACACCGCCGTCTGCTTTCATCTGACTAGGACTGATGAG--

-----CACAAGCACATACTTCATTTTTATTCATACCAC------------
------------------TGCAAATATTATATGTTATGTTTCTTCTGTTG
GGTG------------------CCACCCCTGGTG------CCGTGGCTGA
ACCGACTCTTCCTGTGGCGGTGTTT-------------GCGCCGCCGAAG
CAGCGGGTGGACCTGAGCCATCGGGTG------TTCAACTACGAGTGTCG
C------TTCAAGCAGCATGTCAAC---GAGTGG----------------
--------------------------TCCATCCCAAGGCAAAAGACTGCC
GTGGCTCTGTGGAAACTCAAAGAGTGGATC---GACAACACACCGGAC--
---ATGTACG
TGCATATTCCAGTGGAGGTTCGGTTTGTGCGCCAG---------------
---------------------GACGACATCTTCCTCAGCCCAGCGTGTGG
A---CGTGACTCG-------------------------------------
--------------------------------------TGCTACATCAAC
GTC---------------ATCATG------CCTTACGGTAGGACG-----
-GTCCCCCACGAG---CGTTACTGGGCAGCCTAC---GAGGGAATCATGA
GAGGCCTTGGCGGCAGACCTCACTGGGCCAAG------------------

>Euroglyphus_maynei_(Acari)_partial

---------------------GGTATCATAGCCGACTATGTCATGGCTAT
GCGTTTGATAACACCTTCCGGAGATATAATATATTGTTCAAAAGAGACTG
GCAGTCAGCATTCTGATGTGTTCTTTTCGGCGTTATGTGGCCTGGGCGCA
CTTGGTATTATATTGGAAGTAACCATCCAATGTGAACGTAAATTCTTTCT
TCATCAGCTAACTTATCCGAGTCGATTAGATTCTGTATTGGAACGATTGG
AT------GAGAATGTTGAATCATGTGATCATTTTCGTTTTCTTTACTTT

CCACATACCGATTATGTTTCCGTTTCCATAACGAACAAA-----------

--------AGGATAAGTGATTGGATCGTCAACTATGGT------------
------------------ATTGGTTATCATCTACTTCAATTTGCCTATTG
GTTATCGACT------TATGAACCTAAAATAGTG------CCCATCATCA
ATCGATTAGCATTCTGGCTACTCTAT------------TCATCCAATCAA
GTCAAATTGGACATTAGTTATAAAGTA------TTCAATTTTGAATGTCT
A------TTTGCTCAACACGTAAAC---GAATGG----------------
--------------------------TCAATTCCAAGTGAA---------
---GCGTTACTCGAACTAAAAACAGCAATC---GATAATGAT---GGC--
---CTATACG
CACATTTTCCGATCGAAGTTCGATTTGTTCGACAA---------------
---------------------TCATCCATCTATCTAGCGCCAAGTTATGG
T---CGTGATTCC-------------------------------------
--------------------------------------ACATACATTAAC
ATT---------------ATCTCATATAGACCATATGGACGACAT-----
-GTTGATCACACC---GATTATTGGAATCGATTT---GAAGAGATTATGA
AACGAAACGGTGGCCGACCTCATTGGGCTAAG------------------

