# Supplementary material for: Multiple independent L-gulonolactone oxidase (GULO) gene losses and vitamin C synthesis reacquisition events in non-Deuterostomian animal species
Source: BMC Evol Biol. 2019 Jun 18;19:126. doi: 10.1186/s12862-019-1454-8 (PMC6582580; doi:10.1186/s12862-019-1454-8)
Supplement: Supplementary file 5 — Nucleotide alignment used in the phylogenetic analyses presented in Fig. 2. (PDF 188 kb) [file 12862_2019_1454_MOESM5_ESM.pdf]

>Mus_musculus_(house_mouse)_Muridae_NP_848862.1

-ATGGTCCATGGGTACAAAGGGGTCCAGTTCCAAAACTGGGCGAAGACCT
ATGGCTGCAGTCCAGAGATGTACTACCAGCCCACATCAGTGGGGGAGGTC
AGAGAGGTGCTGGCCCTGGCCCGGCAGCAGAACAAGAAAGTGAAGGTGGT
GGGTGGCGGCCACTCGCCTTCAGACATCGCCTGCACCGATGGCTTCATGA
TTCACATGGGCAAGATGAACCGGGTTCTCCAGGTG------------GAC
AAGGAGAAGAAGCAGGTCACAGTGGAAGCCGGTATCCTCCTGACTGACCT
GCACCCACAG---CTGGACAAGCATGGCCTGGCCCTGTCTAATCTGGGAG
CCGTGTCTGATGTGACGGTTGGTGGCGTCATTGGGTCTGGAACACATAAC
ACCGGGATCAAGCACGGTATCCTGGCCACCCAGGTGGTGGCCCTGACCCT
GATGAAGGCTGATGGAACAGTTCTGGAATGTTCTGAGTCA---------A
GTAATGCAGATGTGTTCCAGGCTGCAAGGGTGCACCTGGGCTGCCTGGGT
GTTATCCTCACTGTCACCCTGCAGTGTGTGCCACAGTTCCACCTTCTGGA
GACATCCTTTCCTTCGACCCTCAAGGAGGTCCTTGACAACCTGGAC---A
GCCACCTGAAGAAGTCTGAGTACTTCCGCTTCCTCTGGTTTCCTCACAGT
GAGAACGTCAGCATCATCTACCAAGATCACACCAACAAGGAGCCCTCCTC
TGCA---------------------TCTAACTGGTTT-------------
--------------TGGGACTATGCCATTGGGTTCTACCTCCTGGAATTC
TTGCTCTGGACCAGCACC---TACCTGCCACGCCTCGTGGGCTGGATCAA
CCGCTTCTTCTTCTGGCTGCTGTTC------------AACTGCAAGAAGG
AGAGCAGCAACCTCAGCCACAAGATC------TTCTCCTACGAGTGTCGC
TTCAAGCAGCATGTCCAAGACTGGGCCATCCCCAGGGAGAAGACCAAGGA
GGCCCTGCTGGAGCTAAAGGCCATGCTG---GAGGCCCACCCCAAG----
---GTGGTAGCC
CACTACCCCGTGGAGGTGCGC-----------------------------
-------TTCACCCGAGGTGATGACATCCTGCTGAGCCCGTGCTTCCAG-
--AGGGACAGC---------------------------------------
------------------------------------TGCTACATGAACAT
CATTATGTACAGGCCCTATGGGAAGGATGTGCCT---CGGTTGGATTACT
GGCTGGCCTAT---GAGACCATCATGAAGAAGTTTGGAGGCAGGCCCCAC
TGGGCAAAGGCCCAC-----------------------------------
-------------------------------AATTGCACCAGGAAGGACT
TTGAGAAAATGTAC---CCCGCCTTTCACAAGTTCTGTGACATCCGCGAG
AAGCTGGACCCCACTGGAATGTTCTTGAAT------TCGTACCTGGAAAA
G---GTTTTCTAC-------------------------------------

>Xenopus_laevis_(African_clawed_frog)_Pipidae_OCT81467.1

-ATGGTTCTTGGTCGTGGAGGATACAAGTTCGAGAACTGGGCCCAGACAT
ACGGCTCCAGCCCAGAGCTTTACTTCCAGCCCACGTGTGTAGAGGAAATA
AAGGAAATTCTCGATCTGGCTCGTCAGAGAAGCAAAAGAGTGAAGGTGGT
GGGAGGCGGTCACTCTCCGTCGGACATAGCCTGCACTGATGACTTTATGA
TCCGAATGGATAAGATGAACAGAATTCTCAAGGTG------------GAT

AAGGAAAAGAAGCAGGTAACAGTGGAAGGTGGCATGCTCCTGACCGATCT
CAACAAGGAA---TTAGATAAGCGTGGCCTGGCTCTGTCCAATTTAGGAG
CCGTATCTGAGGTTGCTGCGGCTGGCGTCATTGGGACGGGCACCCACAAC
ACTGGCATCACTCATGGCATATTATCAACACAGGTGGTGACTCTAACACT
GATGACGGCATTGGGGGAGATCCTGGAATGCTCCGAGGCT---------T
CGAACCCAGAAATATTCCAAGCTGCGAGACTCCATCTGGGCTCTCTAGGG
GTGATACTCAGCGTCACTATTCAGTGCAGATCTGCCTTCCACCTGAAAGA
GGTTCCGTTCTCCTCCACTCTCCAAGAGGTCCTCGACAACCTCGAC---A
CTCATCTGAACAGCTCGGAATATTTCCGCTTCTACTGGTTTCCGCACACA
GAGAATGTCAGCGTCTTTTATCAGGACCCAACAGACAAGCCCCCATCTTC
CAAA---------------------GCAAACTGGTTT-------------
--------------CGGGACACTTTTCTGGGAAATTACCTGCTAGAGTTC
CTGCTCTGGATAAGCACT---TTCATAACAGGAATGGTTCCTTGGATCAA
CCGGTTCTTCTTCTGGCTGCTGTTT------------GCAAGTAAAAGTG
AACAGGTCAACATTAGTCATAAAGTA------TTCAATTTCGACTGCCTC
TTCAAGCAGCACGTACAGGACTGGGCTATTCCCATAGAAAAGACCAAAGA
TGCATTGATGCAGCTGAAGGGTTGGCTG---GAGAAGAACCCGCAT----
---GTAGTGGCG
CATTTCCCAGTGGAGGTCCGA-----------------------------
-------TTTGCCCGTGGGGATGATATATTGATGAGCCCTTGTTACCAT-
--CAGGACAGC---------------------------------------
------------------------------------TGTTACATGAACAT
TATTATGTACAGGCCTTATGGGAAAGATGTTGCG---CACCAAGAATACT
GGGTAGAATAT---GAGAATATCATGAAAAAAGTTGGAGGAAGACCCCAC
TGGGCCAAGGCTCAC-----------------------------------
-------------------------------ACCTGCACCCGCAAAGACT
TTGAAAAGATGTAC---CCGGCGTTCAGCACATTCAAGAGCATCCGGGAG
AAACTGGACCCAACGGGAATGTTCCTAAAT------GCTTATCTGGAGAA
G---GTCTTCTAC-------------------------------------

>Priapulus_caudatus_(priapulids)_Priapulidae_XP_014666894.1
--AT
GGCCGAATGGGGTATTAAAGGGTACCTATTTGAAAACTGGGCCCAGACGT
ACAAATGCATCCCTGAATTATACTTTACTCCAAAGACAAAGGAGGATGTC
ATACAGATTCTAGAGCAGGCGCGGGCAGGAGGCAAGACGGTCAGGGCCGT
TGGTGGTGCACGGTCCCCATCAGACATTTGCTGCACCGATGGGTACATGA
TTGATATGCAGCGCATGGATAGCGTGATTGATGTA------------TGT
GCAGAAAAATGTCAAATAACAGTTGAAGGAGGAGTCATGCTGACAAAGTT
GAATAGGATT---TTAGAGGAGAATGGCATGGCTATCAGTGTACTTGGCG
CTATATCTGATATCACGTTGGCAGGAGCCATGGCAACTGGAACCCATGGG
TCAGGAGCTCAGTATGGAGTTCTAGCATCTTACGTTGTGAAGCTGGAGCT
GCTGACTGCGAGTGGTGAGGTGATGACGCTGTCGAGGGAA---------A
AGGACGAGGACATGTTCCGAGCTGCCGCAGTCAGCCTCGGTGCGCTGGGC
ATCATTCTCACTGTAACACTGCAGTGCGAGAAGGCTTTCAAACTCTGCAA

AACGTCGTTCACTACCACAATGGACGATGTTCTTGAGAATTTGGAG---G
TTCATGTCAGGTCTTCTGAGCACTTTCGCTTCATGTGGATTCCATACACC
GGACATGTCATTGCTACGCATGCAAACAGGACTGATAAAAAAATTGCGGT
GCAT---------------------TCAAGTTGGTTC-------------
--------------TGGGAAACTTTGGTTGGCTACCACATGTTGGAGTTC
ATGCTGTGGATAAGCACA---TACTTCACATCTATGGTGCCATATATTGC
ACGCTTTATGTACAATATTTTGTGG------------AGGCATGGTGGAG
AGATGGTAGACACTAGCTACAAGGTC------TTCAACTTCAACTGTCTT
TTCAAGCAGTATGTGAACGAGTGGTCAATCCCAAGGAGTCAGGCCGTGCT
GGTTTTACTGGAGTTACAAGAATGGATATTGGCACAGGAACACAGG----
---CTGCAGGTA
CACTTCCCCATTGAGGTGCGC-----------------------------
-------TTCGTGAAGGCAGACGATCTTTTGTTGAGCCCATGCTCTGAA-
--ATCGATAGT---------------------------------------
------------------------------------TGTTACATCAACAT
CATCATGTTTCGTCCGTATGGCCGCGATACACCA---CACCAGGAGTATT
GGGACAAGTAC---GAGTCCATTATGAAGAGAGCCGGAGGAAGGCCCCAC
TGGGCAAAGGCTCAC-----------------------------------
-------------------------------AGAGAAACAGCAAGTGATC
TGCGCAAGATGTAT---CCGCATTTTGAACAGTTCTGCAACATTCGCAGT
AGCCTGGATCCACACGGACTATTCCTTAAT------AACTACTTGAAGAG
A---ATATTGGAATATAAG-------------------------------

>Lingula_anatina_(brachiopods)_Lingulidae_XP_013393535.1
--ATGGATAT
ACAGAGTCTCGGCAGAACTGGCCATCTTTTCACTAACTGGGCCACAACAT
ATTCCTGCCAACCTGAACTACATTTTGAACCAGAGACAGAAGAACAGCTC
AAACAGATCCTGAACACTGCACAGATCAAGAATAAGAAGGTGAAGGTGGT
GGGTTGTGGTCATTCCTTCTCAGATCTGGTTTGCACTACTGACTATATGA
TCAGCATGAAGAACTTTGACAGAGTCATATCAATT------------GAC
AAGGAGGCATGTAAAATCAAAGTTCAAGCTGGAATTGAAGTCAAGAAACT
GAAAGATGAAATTCTGCCACAGCATGGACTGGCATTTAGTGTGTTGGGGT
CTGTGTCAGAAGTGTCCTTAGCAGGGTGTATATGTACTGGAACTCATGGC
ACTGGAGGAAACTATGGGGTCTTGTCATCTTATGTGTTGGGTTTGGAGAT
CATGACAGCTAATGGAGAGATCATTGAATGCAGTAAAGAG---------A
AGAACCAGGAAGTATTCTTGGCTGCATGCTGCAGTCTGGGAGCTCTTGGT
GTCATCATGTCTGTCACATTTCAATGTGAACCTGCATTTCACCTACATCA
GAGTCAATACAGGGACACTTTGAACAATGTTTTGGATAACCTGGAT---T
CCCATATCACCTCCTCAGACCACTTTCGCTTTGTGTGGATACCTCACACA
GATCACGTAGTTGTGTACAAAGCCAACAGAATATCAGCGCCCATTTGTAG
GAAA---------------------GACAGTTGGTTC-------------
--------------TGGAATTATGCTGTGGGGTGCCATATGTTGCAGTTC
TGCCTTCTCATCAGTGCT---TTCTTCACTTCTCTTCTTATTTATATGAA
CCGACTCTTCAGTTGGCTACTGTTC------------TCTAAACCAAAAG

AGATTGTAGATAGGGGTGACAGGGTT------TTCAATCTTGACTGTCTT
TTCCAACAATATGTCATGGAATGGGCAGTCCCAAGGGAAAAGACTGCTGA
GGTTTTGTTAGAGCTCAAGGACTGGATA---GAGACAAAC---CAG----
---TTTCCAGCA
CATTTTCCGGTAGAAGTGAGA-----------------------------
-------TTTGTCATGGCGGATAATATCTATCTCAGTCCAGCATACAAC-
--ATGGATTCC---------------------------------------
------------------------------------TGCTACATTAATAT
CCTTATGTTCAGGCCATACAACAAGTATGTTCCA---CATGAGACATACT
GGAATGCCTTC---CAAGATATCATGCTCAGAGCAGGTGGAAGACCACAC
TGGGCAAAGGACCAT-----------------------------------
-------------------------------GGTGTTGTAGCTGATCAGT
TTAGAAAAATGTAT---CCAAAGTGGACAAAGTTCTGTGAGATTGCCCAA
AAAGTAGACCCTCATGCCATGTTTAGGAAC------CCCAACCTGGAGAG
A---GTGCTGGGAAGAAAT-------------------------------

>Gallus_gallus_(chicken)_Phasianidae_XP_015140704.1

-ATGGTTCACGGCCAAGGAGGATTCAAGTTCCAGAACTGGGCCAAGACGT
ATGGCTCTTCCCCAGAGCTGTACTTCCAGCCCACCTCAGTGGAGGAGATC
CGGGAGATCCTGGATATGGCCCGGCAGAGGAACAAGAGGGTGAAGGTGGT
GGGGGGCGGCCACTCGCCCTCTGACATCGCCTGCACTGATGACTTCATGA
TCCAGATGGGGAAGATGAACAAGGTCCTCAAGGTG------------GAC
AAGGAGAAGCAGCAGGTGACGGTGGAAGGTGGGATCTTCCTCTCGGATCT
GAACGTGGAG---CTGAGCAAGCACGGGCTGGCACTGGCCAACTTAGGAG
CCGTTTCTGAGGTGGCAGCAGCTGGTGTGATTGGGACAGGGACGCACAAC
ACTGGGATCAAGCATGGCATCCTCCCCACCCAGGTTGTAGGGCTCTCACT
GCTGACAGCCTCAGGGGACATCCTGGAGTGCTCCGAGTCC---------A
TCAATGCAGATATCTTCCAGGCTGCCCGCCTGCACCTTGGCTGCCTGGGT
GTTGTGCTCACCGTCACCTTCCAGTGCGTGCCCCAGTTCCACCTGCACGA
GGTGACCTTTCCATCCACCCTCACTGAGGTCCTCAATCACCTTGAT---G
ACCACCTAAAGAGATCCCAATACTTCCGATTCCTGTGGTTCCCACACAGT
GAGAACGTCACTGTCATCTACCAGGACCCCACCAACAAGCCCCCCTCTTC
CTCC---------------------GCTAACTGGTTT-------------
--------------TGGGATTATGCTGTTGGGTACTACTTGCTGGAGTTT
CTCCTCTGGATCAGCACC---TTCGTGCCCAGCTTGGTGTGCTGGATCAA
CCGCTTCTTCTTCTGGCTTCTCTTC------------AGCTCCCGGGTGG
AGAACATCAATGTCAGCTACAAGATC------TTCAACTACGAGTGCCGC
TTCAAGCAGCATGTGCAAGACTGGGCCATCCCCATTGAGAAGACAAAGGA
AGCACTGCTGGAGCTGAAGGCTGCCCTG---GAGAACAACCCCAAG----
---ATGGTGGCC
CACTACCCTGTGGAGGTGCGC-----------------------------
-------TTTGCTCGAGCGGATGAGATCTGGCTGAGCCCCTGCTTCCAG-
--AGGGACAGC---------------------------------------

```
------------------------------------TGCTACATGAACAT
CATCATGTACAGGCCCTATGGGAAGAACGTGCCC---CGGCTCAACTACT
GGCTGACCTAC---GAGGGCATCATGAAGAAGTATGGTGGGAGACCACAC
TGGGCAAAGGCCCAC-----------------------------------
-------------------------------AGCTGCACCCGCAAGGATT
TTGAGAAGATGTAT---CCGGCCTTCCCCAAATTCTGCTCCGTCCGGGAT
AAGCTGGACCCTACAGGGATGTTCCTGAAC------ACCTATCTGGAAAA
G---GTGTTCTAC-------------------------------------
--------------------------------------------------
--------------------------------------------------
--------------------------------------------------
--------------------------------------------------
--------------------------------------------------
--------------------------------------------------
--------------------------------------------------
--------------------------------------------------
--------------------------------------------------
--------------------------------------------------
--------------------------------------------------
--------------------------------------------------
--------------------------------------------------
--------------------------------------------------
----------------
>Alligator_mississippiensis_(American_alligator)_Alligatoridae_KY043973.1
--------------------------------------------------
-ATGGTTCACGGCCATGGAGGCGTCCAGTTCCAGAACTGGGCCAAGACCT
ATGGCTCCTCCCCGGAGCTGTATTTCCAGCCGACGTCGGTGGAGGAAATC
AAGGAGATTTTGGAGCTGGCCCGGCAGCGGAACAAGCGGGTGAAGGTGGT
GGGAGGTGGACACTCGCCCTCTGACATCGCGTGCACGGACGGCTTCATGA
TCCAAATGGGGAAGATGAACAAGATCCTCAAGGTG------------GAT
AAAGAGAAGAGGCAAGCGACAGTGGAAGCAGGGATCCTCCTCTCTGATCT
GAACGTCGAG---CTGACCAAGTATGGCCTGGCGCTGGCCAACCTAGGAG
CTGTCTCCGAAGTGGCGGCCGCTGGGGTGATCGGCACCGGGACACACAAC
ACGGGCATCAAACACGGGATCCTGCCTACCCAGGTGGTGGCGCTGACCCT
GCTGACGGCGGCGGGGGAGATCCTGGAGTGCTCCGAGGGC---------T
CCCACGCGGACATCTTCCAAGCAGCCCGGGTGCACCTCGGGTGCTTGGGC
GTCGTGCTTACCATCACCTTCCAGTGCGTGCCGGAGTTTTACCTGCAGGA
GACCACCTTCCCCTCCACCCTCAAGGAGGTCCTGGACAACCTCGAC---A
GCCACCTGCAGCGCTCCGAGTACTTCCGCTTCCTCTGGTTTCCGCACAGC
GAGAATGTCAGCGTCATCTACCAGGATCACACCAGCAAGCCTCCCTCCTC
CACA---------------------GGCAGCTGGTTC-------------
--------------TGGGACTACGCCATCGGCTACTACTTGCTGGAGTTC
CTGCTCTGGGTCAGCACG---TTCCTGCCCTTCCTGGTGCCCTGGATCAA
CCGCGGCTTCTTCTGGCTCCTCTTC------------ACCAGCAAGGGGG
AGAAGGTCGACCTCAGCTACAAGGTC------TTCAACTACGAGTGCCGT
TTCAAGCAGCATGTCCAGGACTGGGCCATCCCCATTGAGAAGACAAAGGA
TGCCCTCCTGGAGCTGAAGGCCGCACTG---GAGAGCAGCCCCAAG----
-----------------------------------------GTGGTGGCT
CACTACCCCGTGGAGGTGCGC-----------------------------
-------TTCGCCCGGGCAGACAGCATCCTGCTCAGCCCCTGCTTCCAG-
--CGCGACAGC---------------------------------------
------------------------------------TGCTACATGAACAT
CATCATGTACAGGCCCTATGGGAAGGACGTACCC---CGACTGGACTACT
GGTTGGCCTAC---GAGGGCATCATGAAGAAGTTTGGAGGGAGGCCACAC
TGGGCCAAGGCTCAC-----------------------------------
-------------------------------GCCTGCACCCGGAAGGACC
TTGAGAAGATATAT---CCCGGCTTCCCGAAGTTCTGCTCCATCCGGGAG
AAGCTGGATCCTACAGGGATGTTCCTGAAC------ACCTACCTGGAGAA
```

G---ATCCCTTGGGACTTAACCCAATCCTTGTCCAAGAGCACTGCTCCCC
TGGGGTGGTTCAGCTTTGACCTGGCG------------------------

>Lepisosteus_oculatus_(spotted_gar)_Lepisosteidae_XP_015207781.1

-ATGATACAGGGAACTGAAGGATATCATTTCCAGAACTGGGCTCAGACAT
ACAGTTGCCACCCTGAGCAGTACTTCCAGCCCAGCTCTGTGGAAGATGTC
AGACAGATCCTGGAACTCGCTAGGCGTTGCAGCAAGAGAGTGAAGGTGGT
GGGTGGGGGGCACTCTCCCTCAGACATCGCCTGCACAGATGACTTCATGA
TGCGTATGGATAAGATGAATAAGGTTCTTGAGGTC------------GAT
GAAGAGAAGAGACAGGTCACAGTGGAAGCTGGGATATTTCTGCGTGATCT
GAATGAAGAA---TTAGCAAAACATGGCCTTGCCCTCTCAAATTTAGGAG
CCGTGGCAGAGGTGTCTGCTGCTGGCGTAACTGGAACTGGTACCCACAAC
ACAGGGGTAGATCATGGCATTCTGTCTACGCAGGTCATGCGTCTGAGTCT
GATGACTGCCAGTGGAGAGGTACTGCACTGCTCAGAGTCA---------G
TGAATGAAGACATCTTTCAGGCTGCTAGGCTTCACCTGGGCTGTCTGGGA
GTCATCATCACCATCACCTTCCAGTGCCAGCCTGCCTTTCGACTTCATGA
GCGCCAGTTCCCCTCCACACTCACAGAGGTTTTGGATAATTTGGAT---T
TCCACAGAAAGAAGTCAGAATATTTCCGATTCCTATGGTTTCCCAATACA
GAACATGTCAGAATAATATATCAGGATCGCACAGAAAAGCCTGCACAGTG
TACA---------------------TCCAGCTGGTTC-------------
--------------TGGAGCTATGCTGTTGGATATCATCTACTGGAATTT
GTCCTTTGGGTCAGCACT---TTCTTTCCTGGCCTGGTTTGCTGGATCAA
CCACCTCTTCTTCAAGTTACTCTAC------------AGCGGCAAAGAGG
AGCGAATAGGCCGCAGTGACAAGGTC------TTCACTTATGAGTGTCTC
TTCAAACAGCACGTACAGGATTGGTCTATTCCCATGGAACAGACTAAAGA
GGCTCTGCTGCAGCTGAAGGCCTGGCTG---GAGAATAACCCAGAC----
---ACAGTAGCT
CATTTCCCGGTGGAAGTGCGT-----------------------------
-------TTTGCTCGGGGAGACAACATTCTCCTCAGCCCCTGCTTTCAG-
--AGAGACAGC---------------------------------------
-------------------------------------TGCTACATCAACAT
CATCCTGTACAGGCCTTATGGAAAGGATGTGCCC---AAACAGCAGTATT
GGGCTGCTTAT---GAAAACATCATGAAGGATGTAGGAGGAAGGCCTCAC
TGGGCAAAGGCTCAC-----------------------------------
-------------------------------AATTGCACCCGTAAGGATT
TTGAGAAGATGTAC---CCTGGTTTCCAGAAGTTTTGCTCTATCCGGGAG
AAGCAGGACCCATCAGGAGTGTTCCTCAAC------ACCTACCTGGAAAA
T---ATTTTCTTC-------------------------------------

>Branchiostoma_belcheri_(Belchers_lancelet)_Branchiostomidae_XP_019645195
.1
------------------------ATGGCGGGAGATGCAGACAGACCGGC
AGTGATGACAGGGCAGACAGGAAGAAAGTTCACCAACTGGGCAGAGACAT
TCTCCTGTGAGCCAGAGCTGTTCTTCGAGCCAACTACTACAGAGGAAATA
AGACAGATCCTGGAAAAAGCCAGACGTCACAGCAAGCGTGTGAAGGTGTG
TGGGTTTGGGCACTCCCCCTCGGACATAGCCTGTACCACAGACTACATGA
TCAGTCTGGCCAAGTACAGACAGGTGTTGGAGGTT------------GAT
GAGGAGAACTGTGTAGTGACAGTCCAGTCAGGAATCCTGCTCAAGGAGCT
GAATGAAGAGGTGTTGCCAAAATACAACATGGCTCTCTCCAATCAGGGAG
CAGTGTCAGAGATCTCTGCTGCAGGGGTCATCTCCACTGGAACTCATGGT
ACAGGCAAGACCTTTGGCATCTTTGCAACACATGTTCTGGAGATGACCAT
GATGACAGCATCAGGAGAAGTACTGAGGTTGTCTCGTGAG---------G
AGAACAGAGACATGTTCCTTACAGCATTGGTGGGTCTGGGCTCCCTGGGA
ATCATCCTCACAGTCAAGATCCAGTGTGAACCTGCTTTTAATCTCCACCA
GGTCCAGTTCAGCTGCTCACTCGATAAGATGCTCAGCACTTTAGAC---A
AGCACCTGGATGAGAATGAACATTTTAAGTTCATGTGGTATCCACATACA
GATGGGGTGGTGGCATTTCTTTCCAACAGAACAAAAAAGCCAGTGCAGAC
CAAT---------------------CCCAGCTGGTTT-------------
--------------TGGGATTATTTTGTGGGCTACTATTTGCTGGAGTTT
CTCCTGTGGTTGAGCACA---TTTTGTTCAGACCTCCTACCATGTATCAA
CAAGTTTTACTACAGCTACATCTAT------------GCTGTACCGTCAG
AGCGGGTGGATCGCAGTGACAAAGTC------TTCAACTTTGAATGTCTT
TTCAAGCAGTATGTGACAGAGTGGTCTTTTCCAAGGAGTGAAACTGCAAA
TGTGCTGCACACTTTACAAGACTGGGTC---AAGTCCCACCCAGAA----
---CACAAAGTT
CACTTCCCAGTGGAGGTTCGG-----------------------------
-------TTTGTCCGATCGGACGACATCTACCTTAGCCCCTGCTACCAA-
--CAAGACAAC---------------------------------------
------------------------------------TGTTACATCAACAT
CATTTCTTACAGGCCGTATGGAAAGGATGCACCA---AAGGATGCCTGGT
GGGACATGTAT---GAGAGCGTCATGCACAAGGTTGGAGGGAAACCTCAT
TGGGCAAAGGCTCAC-----------------------------------
-------------------------------AAGGTGACACCTGAGCAGT
TTCAGAAGCTGTAC---CCAATGTTTTCCAAGTTCTGTGCCGTTCGGGAG
CAGCTGGACCCACAGGGCATGTTCCTGAAC------CCCTACCTGGAGCG
CCACGTTCTCGGCATGACCCCTCAGACACAGTTTGACGTT----------

>Saccharomyces_cerevisiae_S288C_NP_013624_1
---ATGTC
TACTATCCCATTTAGAAAGAACTATGTGTTCAAAAACTGGGCCGGAATTT
ATTCTGCAAAACCAGAACGTTACTTCCAACCAAGTTCAATTGATGAGGTT
GTCGAGTTAGTAAAGAGTGCCAGGCTAGCTGAAAAAAGCTTAGTTACTGT
TGGTTCGGGCCATTCTCCTAGTAACATGTGCGTTACTGATGAATGGCTTG
TTAACTTAGACAGATTGGACAAAGTACAAAAGTTTGTTGAATATCCTGAG
TTACATTATGCCGATGTCACAGTTGATGCCGGTATGAGGCTTTACCAATT
GAATGAATTT---TTGGGTGCGAAAGGTTACTCTATCCAAAATTTAGGCT
CTATCTCAGAACAAAGTGTTGCTGGCATAATCTCTACTGGTAGTCATGGT
TCCTCACCTTATCACGGTTTGATTTCTTCTCAATACGTAAACTTGACTAT
TGTTAATGGTAAGGGCGAATTGAAGTTCTTGGATGCTGAA---------A
ACGATCCAGAAGTCTTTAAAGCTGCTTTACTTTCAGTTGGAAAAATTGGT
ATCATTGTCTCTGCTACTATCAGGGTTGTTCCCGGCTTCAATATTAAATC
CACTCAAGAAGTGATTACTTTTGAAAACCTTTTGAAGCAATGGGAT---A
CCCTATGGACTTCATCTGAATTTATCAGAGTTTGGTGGTACCCTTATACT
AGAAAATGTGTTCTATGGAGGGGTAACAAAACTACAGATGCCCAAAATGG
TCCA------------------GCCAAGTCATGG---TGG----------
-----------------GGTACCAAGCTGGGTAGATTTTTCTACGAAACT
CTATTATGGATCTCTACCAAAATCTATGCGCCATTAACCCCATTTGTGGA
AAAGTTCGTTTTCAACAGGCAATACGGGAAATTGGAGAAGAGCTCTACTG
GTGATGTTAATGTTACCGATTCTATCAGCGGATTTAATATGGACTGTTTG
TTTTCACAATTTGTTGATGAATGGGGGTGCCCTATGGATAATGGTTTGGA
AGTCTTACGTTCATTGGATCATTCTATTGCGCAGGCTGCCATAAACAAAG
AA-------------------------------------TTTTATGTC
CACGTGCCTATGGAAGTCCGTTGCTCAAATACTACATTACCTTCTGAACC
CTTGGATACTAGCAAGAGAACAAACACCAGTCCCGGTCCCGTTTATGGC-
--AATGTGTGCCGCCCATTCCTGGATAACACACCATCCCATTGCAGATTT
GCTCCGTTGGAAAATGTTACCAACAGTCAGTTGACGTTGTACATAAATGC
TACCATTTATAGGCCGTTTGGCTGTAATACTCCA---ATTCATAAATGGT
TTACCCTTTTT---GAAAATACTATGATGGTAGCGGGAGGTAAGCCACAT
TGGGCCAAGAACTTCCTAGGCTCAACCACTCTAGCTGCTGGACCAGTGAA
AAAGGATACTGATTACGATGACTTTGAAATGAGGGGGATGGCATTGAAGG
TTGAAGAATGGTATGGCGAGGATTTGAAAAAGTTCCGGAAAATAAGAAAG
GAGCAAGATCCCGATAATGTATTCTTGGCAAACAAACAGTGGGCT-----

-----------------------------------ATCATAAATGGTATT
ATAGATCCTAGTGAGTTGTCCGAC--------------------------

>Metarhizium_majus_ARSEF_297_XP_014580409_1
ATGCATCCCGCGGCGGAATCCGAGCTGCGAGGCCAAAACGCCAACGGCGT
CGCGTTCCGCGCGAAACCGCACCACGTCCATCGAACCTGGGCGAGGACCT
TCTCCTCGATCCCCGAGCTCTACATCCAGCCAGAGACCCTCGCCGAGGTC

GAAAAGGTTGTTTCTCTCGCCCAGACATGTCGCCGGCGCATCGTCACCAC
CGGCTGCGGCCACTCGCCCTCCGACATCACCTGCACCTCCAGCTGGATGG
TCAACCTCGACCGCTTCAACAAGATCCTTGCCGTC------------AGC
ACGGACACGGGCCTGGTGACCATGGAGAGCGGCATACGGCTGTACGCCCT
GTGCGAGGAG---CTGGAGCGTCACGGTCTTGCCATGCCCAACTTGGGGA
GCATCAACCAGCAGTCCATTGCTGGCGCCATTTCGACGGGAACCCACGGG
AGCAGTGTGCGCCATGGCCTCATGTCTGAAGATGTGCAGTCTCTCCGAAT
CACACTGGCCAATGGGACAACGGAACTCTGCTCTGCCGAG---------T
CGAATCCGGAGCTGTTTCGAGCCGCGCTCCTGTCTCTCGGCGCATTGGGC
ATCATCACCGAGATTACGCTTCGCGCGGTCCCCGCCTTCAAGCTCAAGTG
GACGCAGACCATCGACACCGACTACAAGCTGTTCAAGAGATGGCCGCTCG
ACCTGTGGACGCAGAGCGAGTTTGTCAGGGTGTGGTGGTTCCCCTACACG
AGACGCGCCGTCGTGTGGCAGGCAGACAAAACCACGGAACCCGAGCAGGA
CCCC---------------------CCGACGAGCTACTAC----------
-----------------GACGGATCCCTGGGGTACTACGTCTACCACAAC
CTGCTCTACCTGGCGCAG---TTTGCTCCCCGGATCCTCCCCTGGGTCGA
GTGGTTCGTCTTCGGCATGCAGTACGGCTTCGCCAACGGGTCGACCACGA
CGGCCGTCCAGCCGAGCCGAAGGGCG------CTGCTGATGAACTGCCTC
TACTCGCAGTTTGTGAATGAGTGGGCGATCCCCCTGCACAGGGGGCCCGA
GGCGCTGCGGCGACTCAGCGCCTGGCTGAACCGCCTCACGCCCGACGACC
CGGACTACGTGGCGCACGGCATCCCCTTCTCGGCCGACGGGCTGTACGTG
CACGCCCCCGTCGAGGTGCGGGTGAGCGACACGAGC--------------
-------CTCACCTCCAACCTCCGTCCGTACCTGGACCCGACTGTCCCCG
ATGGCCCGACG---------------------------------------
------------------------------------CTGTACCTCAACGC
CACCTTGTATCGGCCGTACTGGCGGGACCCGCCGTGCCGCGACCGCTACT
ACCAGGCGTTT---GAGTGGTTGATGAGGGACATGGGCGGCGGCCGCAC
TGGGCCAAGAACTTT-----------------------------------
-------------------------------GAGACGTGTCGGCCTGATA
TCGAGGCCATGTATGGCGAGAGCTTGGAGGCGTTTCGGAGGATACGCGAC
GGCGTGGACCCGGAGGGCATGTTTGTCGGC------CCGTGGCACAGAGA
ACGCATCATGGCACCTCTGCCCGGATCCAAGCTTCAGCTGGAAGAGGTGG
AGGTTTCAAGGAGGAAGGCTGCTGGCGGCGGTCTCGTGATGGAGGGGCTT
GTCGGAGGAGGT--------------------------------------

>Nematostella_vectensis_(starlet_sea_anemone)_Edwardsiidae_EDO44935.1
------------------------ATGTCAAGCTTGGAAACAAAGAAGGT
TTTGGCGCGAGGAAAAAAGGGACACCGGTTTACCAACTGGTCTACTACAT
ATTCTTGCTCCCCTGAATTATACTTCGAGCCAGAATCCACCGATGAAATA
CGGCAGATTTTGCTAATTGCAAAGGAGCTCCATAAGCGTGTGCGTGTGGT
AGGGTCTGGCTACTCCCCGTCTGATCTTGCTTGTACCCCAGAGTATATGA
TGAGTTTGAAGAAGTACAAAGCTGTTTTAGAGATT------------GAC
CAGGAGAAACAGCAAGTAACAATGCAAGCAGGGCTCACACTCACCGAGCT
CCACACCGTG---TTGGACATTCATGGACTTGGATTCCCAAACCTTGGTG
CAGTGTCGGGTATCTCCTGTGCTGGGGTGATCTCCACCTGTGTGCATGGA
ACTGGGTTCAACTATGGTCTCCTAGCAACACATGTTTCAAGACTGGATAT

AATGACGGCAAGTGGAGAAATTATCACCTGCAGTAGAATG---------C
ATAATGAAGACATCTTCAGATCTGTCATTTGTGGGATGGGTTCAGTTGGT
GTTATTCTTTCCATTACATGGCAGTGTGAGAAGACCTTTAGTCTTTGCTT
AAACAAGAAATCCTTCTATTTAAATGAGATGCTTGAGGACTTGGAG---G
CACAGCTTGTGTCCTGTGATCACTTCAAGTTCTTTTGGTATCCACACACT
GACAAAGTTGTTGCAGAATATGCAAGTAGAACAACAAGGGGCCATACTCC
TCCA------------------CAGAATAGCTGGTTC-------------
--------------AGGGACATGTTGATTGGGTTTTATTTGCTGGAGTTT
CTTTACTGGTTAAGTATT---TTTATCCCAGCTCTTGTGCCTCTGATAAG
CAAGTTGTATTATATGATAGCAACA------------TCGGGTAGCAAGG
AGAGAATTGACAGAAGCTACAAGATT------ATGAATTTTAATTGTTTG
TTCAAGCAGTATGTGACAGAATGGTGTATTCCAAGAAATAAAGTTGCAGA
TGTCTTGAGGACGTTACGAGATTGGACT---GAGAAAAGT---GGA----
---TACAAAGTC
CATTTTCCTGTTGAAGTTCGC-----------------------------
-------TTTGTGAAGGCTGATGATTTCTATCTGAGTCCATGCTACAAG-
--ACTGATTCC---------------------------------------
------------------------------------TGTTTCATTAACAT
CATCTGCTACAGGCCATACAACCAGTTTGTTGCC---CATGATGCCTACT
GGAGATTCTAC---GAGAACCTGATGGATTCCGTTGGAGGCAAGCCTCAC
TGGCTAAGGCTCAT------------------------------------
-------------------------------AATCTGTGTGCCGCTGACA
TGGAAAAGAAGTAC---CCGATGTTCAACAAATACCGGGAAGTTTGCCAG
AGGCTAGACCCACAAGGAATCCTGAGGAAT------TCAAATGTAGACTG
CACCATTTTTGGTAAAACT-------------------------------

>Parasteatoda_tepidariorum_(common_house_spider)

-ATGAAGACCGGAATTCAAAATATTCATGTAAAAAATTGGGCTGAAACAT
ATGAATGTAGACCAGACCTTTTCTTTGCTCCAAAAACAGCAGAAGAAATA
TCTCAGATTTTGGACGTAGCTAAGCAAGAAGAAAAACGAGTAAAAGTTGT
TGGTTGTCGACATTCTCCTTCAGACATAGCTTTTACCAATGGAATTATGA
TAAGTTTGCAACACATGAATAAAGTTTTAGAGATT------------GAT
AAAAACAGTAAAAGAGTTAAAGTTGAAGCTGGAATCCTTTTGAGAGATTT
GAATAAAACT---CTTTATTCGAATAATCTTGCATTGTCAGTACTTGGCT
CTGTATCTGACATAACACTTGCTGGTGCAATGTGTGTTGCCACTCATGGG
ACTGGTTTAGAATATGGTACCATATCATCTTATGTCACAGAATTGGAAAT
GATGCTGGCAGATGGTTCTGTAAACACATATTCAAGTGTA---------A
GAGATGGAGATATTTTTCAGGCAGCTCTGTGCAGTCTTGGGTCACTAGGA
ATTATTTTGACTGTCACTATACAATGTGAAGAAGCTTTTAATTTACAAAT
GAGGCAATATGGTCTAAGTTTAAAAGATGTTATTGAAAACCTAGAG---G
TACATCTCAATGGTTCCGATCATTGCCGATTTATGTGGTTTCCATACACA
GATAGTGTTGTCATGTCTCATGCAACTCGTACAGAATTACCAGCCTTGAA
AGTA------------------------ACATGGATA------AGCAAGA

TATGGAATATGATATTTGATTATGGTGTTGGTTATCACCTTTTAGAATTT
TGTTATTATATAAGCACG---TTTATGCCTCATATAGTACCATACATAAA
TAGATTCTTTTACTACACAGTTTTC------------TCTGTTTACACTA
GAAAAATTGATAGAAGTTATAATGTG------TTCAACTTTGAATGTCTC
TTTAAGCAGTATGTTAATGAATGGGCTATTCCTATTGAAAAAACTGGCGC
TGTTCTATGGGAACTTAGAGAGTGGATT---GAGACTACCCCAGAA----
---GTATATGTT
CATTTCCCAATAGAAGTTAGA-----------------------------
-------TTTTGCAAAGCAGACAACATTTTCCTTAGTCCAGCTCATGGA-
--AGAAATACA---------------------------------------
------------------------------------TGTTATATTAATAT
TTTAATGTACAGGCCATATGGGAAAGATGTACCT---TATGAAAAATATT
GGGCAGCATAT---GAAAAGATAATGATGGAAGCTGATGGCAGGCCTCAT
TGGGCTAAGGCACAT-----------------------------------
--------------------------------TCAGTGACAGCTGATAAAT
TTAGACTGATGTAT---CCCTACTTTGGAAAATGGTGCTCAATACGACAA
AAGTTGGATCCAACAAATATGTTTTTTAAT------TCTTACATGGCAAG
G---ATTTTTTCTCATAGTAGACTA-------------------------

>Capitella_teleta_(Annelida)

----AATACTGGAAAGAGAGGCCATTTATTCGAGAACTGGGCAGGCACCT
ACTCATGTTCCCCGGAAGTATATTTTGAACCAACTAATGAAGAAGAGGTG
CGCCAGATTCTTCAAGTTGCTCGAGATGAGGGCAGGTGCGTCAAAGTGGT
TGGAGGGGGTCACTCGCCCTCGGACATCGCGTGTACCGACGACTTCTTAG
TCTCAATGAAACACTTCAAAAAATTAATCAACGTC------------GAC
AAAGAAAAGCGGTTGTTCACAGCAGAAGGCGGAATAATGATATCAGAACT
GAACGAATTA---CTTCGTGACCGTGGCTATGCCCTCAGTGTATTGGGCT
CCATTTCGGAATTAACTCTGGCGGGCGTTATATGCACTGGGACTCATGGA
ACAGGCATTCAATTCGGCAACTTGGCATCTTCTGTTACGGAAATCGAATT
AATGACGTCTGACGGGGAAATCCGCACGTTGAGTAAAGAG---------G
CAGACGGCGATGTGTTCAGAGCAGCAGCGGTCAGCCTGGGATGCCTCGGA
GTCATCCTAAAAGTGACCGCTAAGTGCGAAGAGGCGTTCAACTTGAAACA
AAACAGCTATGGCGCCAACATCAAAGACTTGCTGGAAAACCTGGAC---G
TCCATCTCAAGGCATCTGACCACTTCAGGTTCATGTGGTACCCGCACACT
GACCAGTGCGTTACATTCCACACCAGAAGAACGCAAGAGGCGGTTCGTAG
GAGC---------------------CACAGCTGGTTC-------------
--------------TGGGATTATCTCATTGGATTCTATCTCCTGGAATTC
CTTCTCTGGATTAGTACC---TGGTTCAAAGGATTTGTGCCCTTGATCAA
TCGAACCTACAGCCGAATCAAC---------------AGCAAACCCTCCG
AGTTCATTGATGTCAGTTATAAGGTC------TTCAATTTCAACTGTCTG
TTCCGGCAATATGTTATGGAGTGGGCCATTCCAATGATAAAAACGCAGCT
CGCTTTGTTTGAATTAAAGAATTGGATC---GGAAATTCA---GGA----
---TTTGAAGCT

```
CATTCTCCGGTGGAAGTTCGT-----------------------------
-------TTTGTGCGTGGGGATGACATGCTGTTGAGCCCTGCTAATGGC-
--CGGGACGTC---------------------------------------
------------------------------------TGTTATGTGAACAT
CATTATA------CCGTACAACAAGTTAGTTGCT---CATGCTGAGTATT
GGGAAGCGTTT---AAGCAGATCATGCTCCGAAATGACGGCAGACCACAT
TGGGCAAAGGATCAC-----------------------------------
-------------------------------CTCATGACCGCCAAGGAGC
TGAGGCCCCTCTAC---CCTAAATGGGACACCTTCTGCCGGATTCGTCAA
AAAATGGATCCAAAGGGGATGTTCATGAAT------GAGAATTTGAAGAA
C---ACCCTTAGCTATTCA-------------------------------
--------------------------------------------------
--------------------------------------------------
--------------------------------------------------
--------------------------------------------------
--------------------------------------------------
--------------------------------------------------
--------------------------------------------------
--------------------------------------------------
--------------------------------------------------
--------------------------------------------------
--------------------------------------------------
--------------------------------------------------
--------------------------------------------------
--------------------------------------------------
----------------
>Trichoplax_adhaerens_(Placozoa)
--------------------------------------------------
-------------CCTTACGGCAAAATTTTCAGCAACTGGGCTAAAACTT
ATCAGTGCCAGCCAGAGCTATATTTTATTCCTGAAAGTATCGATCAATTA
AGAGATGTTATCAAGTTGGCTACAATTCATAAGAAAGTCGTTAGAGTAGT
CGGCTGCGGCCATTCTCCATCGGACATTACGTGCACGTCGGATTACATGA
TCAGTATGGAAAAATTATCCGGTATTCTTGAAGTT------------GAT
GTGGATCGTAATCAAATAAAAGTACAAGGTGGTGTCAAGATTGAGCGGCT
ACATAAAGTC---CTAGAAGAGTATGGCCTTGCTATTAGCAACATCGGTT
CGGTATCCGATATCACTCTATCGGGAGCAATTGCTACTGGTACTCACGCA
ACAGGTGTCGACTTTGGCATGCTTGCTACCAATGTCTTAGAGATTGAATT
ATTGCTAGCCAATGGGGAGCTCATGGTATGTTCTCGACAT---------C
AAAACAAGGAAATTTTTTTGTGTGCTTTATGCAATCTTGGTGCACTTGGT
GTTGTTGTTTCAATCAAATTACAGTGTGAAAAGAGTTTCAAATTAGAGCA
GCAACAATCTTCCTGTACTCTTGACGAGGTATTAGATAATTTGGAA---A
ATGAAGTACGCTCATGCGAACACTTTCGTTTTGGTTGGTTTCCACATACT
AATGATGCAGTT---------------------------CCCAAAGTCGA
TAGT---------------------TACAATTGGTTT-------------
--------------TGGGATAGCATTGTAGGTTATCATTCTCTTCAGTTC
TTC------------TCC---TACTTTGATAGTCTTATCCCATGGATAAA
TAGAACGTTCTTTGGATTGTTGTTC------------CGTAAACCAAAGT
ATCGTGTCGATGATAGCTATAAGGTA------TTCAACTTTGAATGCTTG
TTTAAGCAATACGTCACGGAATGGGCCATACCTAGGGAAAACACTGGCAT
AGTGCTAAGGCAGTTGCGAGATTGGCTG---AACACAAAT---AAC----
-----------------------------------------TTTTATGCT
CATTACCCTGTTGAAGTACGA-----------------------------
-------TTTGTTAAAAGAGATGATGTTTACCTTAGCCAAAATTATGAA-
--CGTGATACA---------------------------------------
------------------------------------TGCCACATCAACAT
CGTTATGTATAGACCTTATGGAAAATTCATTCCT---CATGACCGCTATT
GGAAGGCATAC---GAAGAAATAATGCTTGCTGCTAAAGGAAAACCCCAC
TGGGCTAAGGCGCAT-----------------------------------
```

-------------------------------AGTCTGACGTATAAAGAAT
TGTCACAGATGTAT---CCTCAATATGATAAGTTTTGCAAAATCCGCGAT
GAACTTGATCCCAATCGGATATTTATTAAT------CGTTATTTGACCCA
A---GTTCTCTTTGAAGGCCGGGAAGAG----------------------

>Achipteria_coleoptrata_(Acari)

----------GGAGTGAAGAATATAAAGTTCACGAATTGGTCGCAAACAT
ATTCTTGTCATCCATTCCTTTATTTTGTTCCTAAAGATATCGAAGAATTG
AGAGAAATAATATTTTTGGCCAAAAGTGAGAATAAAAAGATTCGAGTCGT
GGGTTGCGGTCATTCGCCGTCAGATATTTGCTGTACTAATGATTATATGA
TTAGTTTAAGGCTTTTTAATAATGTATTGAATGTG------------AAT
CGAGAGAAACATACGGTTAGTGTTGAGACCGGTATTACTTTGACTGAACT
CAACGCATAT---CTTGACTCTCAAAAGTTGGCGCTTTCGGTTTTGGGTT
CGATTTCTGATATAACTGTTGGGGGAGTAATAAGTACTGGAACTCATGGA
AGTGGCGGTAAATTTGGTGTTTTCGCTGATTATGTCTTAGATATGGAGTT
AATCACCACTTCTGGCGATACGATAAAGTGTTCTCGTGAT---------G
AGAATAATGATGTATTTCTTTCATCACTGTGTGGATTGGGTGCCACCGGT
ATCATAACCCGAGTGACCATTCAATGCGAGCCCGGATTCCTTCTCTATTC
AAACAGCTATCCGTCCACTTTAGATGAAGTGCTCGAAGATTTGGAC---G
ACCAAATCAACAGTTGTGATCACTTCAGGCTCTTATGGTTTCCGCACACG
AATTGTGTTTCCGTTTCGAATTATAACCGGATTTACAACAAATCGGTTAC
AAAATATTCTAAATTTCAAATGATTTCTAATTGGGTC-------------
--------------TGGAATTACGGCGTCGGTTACTATACCTTAGAGTTC
GCGTATTGGCTCAGCACT---TATTATCAACCATTAGTCCCGTATATCAA
CAGAATTTGGTTTTGGTTACAGTAT------------TCTCGACCCCAGG
AAGCGATTGATGTCAGCCATAAGATA------TTCAACTTTGAGTGTCTC
TTCAGACAACATGTGAACGAGTGGTCAATACCCAGAGAGAAAACGGCGAA
AGTATTACTGGAACTGAAGGAATGGATT---GAAACGACGCCTAAT----
---ATTTACGTT
CACTTTCCGGTCGAAATTCGT-----------------------------
-------TTCGTCAAAGAGGACGACATTTATTTGAGTCCAGCGTATGGT-
--CGCGACTCC---------------------------------------
------------------------------------ACATTCATTAATAT
AATTATGTACAGACCTTATGGTAAGGATATCTCG---CATTCGATATATT
GGAATCAATAC---GAAAAGTTGATGAAAGAGGCCGGGGGTCGACCCCAT
TGGGCTAAAGCCCAC-----------------------------------
-------------------------------AGAGAGAGTTCTTCTGATT
TCGTTAAAATGTAC---CCGTATTTCAGGGCCTGGACTCAAGTCCGTAAA
CGATTGGATCCGATTAATATGTTAGTCAACTCATATCTCAACAG
A---ATTCTTATCGAA----------------------------------

>Hypochthonius_rufulus_(Acari)

----------------------GTGCACGTGACAAACTGGTCACGCACGT
TCCATTGCGACCCCATTTTGTATTTCATCCCTAAAGATGTTGAGGAACTC
AGAGAGATCCTTCTTTTGGCGGAATCAAATCATAAGAAAGTCCGTGTCAT
TGGAAATTGTCATTCACCGTCTGACATTTGCTGTACAAATGATTATGCAA
TTAATTTGCAACACTTTAATAATGTATTGAGTGTC------------GAT
CATGAGACATGCCGTGTCAAAGTCGAGGCCGGAATGATGTTCGTCGAACT
CATTCAGCAT---TTGGATAAATTGGGAATTGCATTTCCTATTCTCCCGT
CGATATCCGATTTGACCGTTGGTGGTGTCATAAGTACGGGAACACACGGT
ACGGGTATAAATTATGGTATAATCTCGACATTTGTGATTGATATGGAACT
AATCACATCATCGGGTGATACTATAAAATGTTCGCGTAAC---------G
ATAATACCGAAATATTTATGGCGTCACTCTGTGGTTTGGGATCGACCGGA
ATCATAGTCAATGTGACACTTCAATGTGAACCGTCATTTCTGTTACATCA
GCACATATATCCTTCGACTTTAGATGATGTTCTCGATGATCTGACT---G
ATCAAATACAATCAGCAGATCATTACCGATTCTTATGGTTTCCGCACACC
AATTGTGTGTCGATATGGCAAAACACACGTATTTATAATGCACCCGTAAC
TAAATATTCGACACTCCAAAAGGCAATAAATTGGTTT-------------
--------------TGGGATTATGCAATGGGGTATTATACCCTCGAATTT
GCCTATTGGTGCAGTACG---TTTTGTCCACGATTAGTCCCGTACATTAA
TCGTGCAGCATTTTGGTTATTATAC------------TCATCTTCAAAGG
AGAGAATCGAAATCAGTCATAAGGTG------TTTACGTTTGACTGTCTT
TTTCGACAGTATGTCAATGAATGGTCAATACCATTTGAAAATACGGCGCG
CGTTTTACTCGAACTCAAGAATTGGATT---GATTCGACTCCCGAT----
---GTTTACGCT
CATTTTCCGGTCGAAGTTCGC-----------------------------
-------TTCGTTAAGGGTGATGATATTTATTTGAGTCCAGCGTTTGGT-
--CGCGATTCA---------------------------------------
------------------------------------ACGTATATCAATAT
CATTATGTACAGACCCTATGGTAAGGATCAACCG---TTTCAAGAATATT
GGAATAAATAC---GAAGAAATTATGAAAGCGTCCGGAGGACGCCCGCAT
TGGGCTAAAGCACAT-----------------------------------
-------------------------------CGTGAAACTGCTGCTGATT
TTCTTAAGATGTAT---CCCCACTTTAAGACATGGACTCACGTGAGGAAA
CGTTTGGACCCGATTAACATGATGATGAAT------CCGTATATGGAAAG
G---ATATTTAAGAAAATTTCCAAT-------------------------

>Latrodectus_hesperus_(spiders)

------ATATTAGAACTAGCTAGACAAGAAGAAAAAAAAGTGAAGGTTGT
AGGCTGCCGACATTCTCCATCTGATATAGCTTGTACAAGTGGTATCATGA
TTAGCCTGCTGAATTATAACAAAATTTTAGAAATT------------GAT
AAACATAATCGCCGTGTTAAAGTTCAAGCTGGAATAGTTTTAAGTGATCT
AAATGATGTA---TTGTATTCCAATAACCTTAGTCTCTCAGTGCTTGGTT
CAGTGTCTAATATTACCATTGGTGGGGCAATAAGTGTAGCTACTCATGGA
ACTGGCATTGAATATGGTACAATTTCATCTTATGTGACCGAATTAGATAT
AATGCTTGCTGATGGTTCAACTAAAACTTTATCTCCTGAC---------A
AGAATGGGGATTTATTTCAAGCTGCTCTGTGTAGTCTTGGCTCTTTTGGA
ATTATATTAACAGTAACTATTCAATGTGAAGAAGCATTTAATTTGCAAAT
GAGACAATATGGATTGAGTCTAAAAGATGTGATTGAAAACTTAGAT---G
TACATTTGTCAGGCTCAGATCATTTTCGATTTATGTGGTTCCCTTACACA
GATAGTGTTGTTGTTTCTCATTCAATAAGAACTGAATTGCCCCCAATGAA
AATA------------------------TCATGGATT------AGTAGGA
TATGGAATGCAATATATGAATATGGTATTGGATATCACCTTCTTGAATTT
TGCTATTATATCAGTACA---TTCTTTCCTCGTATAGTGCCATATATAAA
TAGATTCTTCTACTATACAACTTTT------------TCGGTGTATACTA
GGAAAATTGATAGGAGCTACAGAATA------TTTAATTTTGAATGTCTA
TTCAAACAATATGTCAATGAATGGGCTATTCCAATAGAGAAAACTGGTGT
AGTTCTATGGCAACTAAGAGAATGGATA---GAATCAACGCCTGAT----
---TTTTATGTA
CATTTTCCAATAGAAGTCAGA-----------------------------
-------TTTACTAAAGCAGACAATATATTTTTAAGTCCAGCACATGGT-
--AGAGATACA---------------------------------------
------------------------------------TGTTATATTAATAT
TCTTATGTACAGACCCTATGGTAAAGATGTACCA---TATGAAAAGTATT
GGGCAGCATAT---GAAAAAATTATGTTAGAAGCTGATGGTAGACCACAT
TGGGCTAAAGCTCAT-----------------------------------
-------------------------------TCAGTGACTGCTGAAATGT
TTAGACTGATGTAT---CCATATTTTGGAAAATGGTGCTCAATACGTCAC
AAGTTGGATCCAACAAATATGTTTTTTAAT------CCTTATATGGCTAG
A---ATTTTTTCTAACAGCCGAATG-------------------------

>Nephila_clavipes_(spiders)

----AAAGACGGAATCCCAAATGTCCACTTCAAAAACTGGGCAGAAACAT
TTGAATGTGTGCCAGAATTGTTTTTTGCTCCAAAATCTCAAGAAGAAATT
GCTCAGATATTAGATTTAGCACGAGAAGAAAACAAAAAGGTGAGAGTTGT
GGGCTCTAAACATTCACCTTCTGATATAGCATGTACAAATGGATTTATGA
TCAGTTTGCAGAATTTTAATAAACTGGTTGAGGTT------------GAC
AAAAAACTTATGAGAGTAAAAGTGCAAGCTGGTATGAAATTAACTGAGCT
TAATGATATT---TTGTATTCTCATAACCTTGGGCTTTCTGTACTTGGAT
CTGTTTCTGATATCACTATCGGAGGAGCAATTAGTGTTGGTACCCATGGT
ACAGGTATCGAATATGGGGTCCTTTCATCCTATATAACTGAAATGGAACT
GATGTTATCTAATAATGCTGTGATAAAAGTCTCTCCTGAA---------A
AAGATGTCGATGTGTTTCAAGCTGCTGTTTGCAGTCTAGGCTCATTTGGG
ATTATTTTAACTGTTACAATACAATGTGAACAAGCATTCAATTTGCACAT
GAGACAATATGGTTTACCTCTAAAAGATGTTGTTGAAAATCTTGAC---G
TACATTTGACAGGATCTGATCATTTTAGATTTATGTGGTTTCCGTATACA
GATAATGTAGTTGTATCTCATACTTCAAGGACCGATTTGCCTGCCAGAAA
AGAA------------------------ACGTGGCTT------AGAAAAA
TTTGGAATCTTTTCTGGAATTACGGCATTGGATATCATGCTCTCGAATTC
TGCTATTATGTAAGTACA---TTCTTTCCACATTGGGTACCACATATCAA
TCGAATCTTCTACTACCTGTTGTTT------------TCAACATCTAGTA
CAAAAACTGATCGAAGCTACAGAGTC------TTTAACTTTGAGTGCCTT
TTTAAGCAATATGTAAATGAATGGGCTATTCCTATTGAAAAAACAGGAGT
TGTATTATGGCAGCTAAGAGAATGGATT---GAAACTACTCCTGAT----
---GTTTATGTA
CATTTTCCAATAGAAGTTAGG-----------------------------
-------TTTACAAAAGCAGACAACATCTTTATCAGCCCTGCATTTGGA-
--AGAGATACT---------------------------------------
------------------------------------TGTTACATAAATAT
TATAATGTACAGGCCGTATGGTAAGTCTGTTCCA---TACAAAACATATT
GGGAGGCATAT---GAACATATTATGTTGCAGGCTGGTGGTCGTCCTCAC
TGGGCAAAGGCTCAT-----------------------------------
-------------------------------TCAATTACAGCACAGACAT
TTAGACACATGTAT---CCATTATTTGGAAAATGGTGTTCAATACGACAA
CAGTTAGATCCCATAAATATGTTTATGAAT------TCCTATATGACTCG
T---ATATTCTCA-------------------------------------

>Platynothrus_peltifer_(Acari)

------ATCATATTTTTGGCCAAAAGTTGCAATAAAAAAATCCGAATCGT
GGGCTGCGGTCACTCTCCATCTGATATTTGTTGTACAAATGATTATATGA
TAAGTTTGAGTTATTTTAATAATGTATTGAATGTC------------AAC
AGAGAGTTGAATACCATTCAAGTCGAGGCCGGCATTACTTTCAGTGAACT
CAATGCACAT---CTCGATAGACACAAACTCGCATTTCCTGTATTGGGTT

CGGTCTCAGAATTAACGATCGCAGGAGTGATCAGTACTGGGACTCACGGA
ACGGGTGCCAAATTTGGTATTATCTCTAGTTATGTAATAGACATGGAATT
AATAACAACATCTGGAGATACTATTAAGTGTTCTCGGGAT---------G
AAAACAGTGATGTATTTCTATCAGCTCTTTGTGGTCTGGGAGCTATTGGT
GTGATAATACGAGTGACCCTTCAATGCGAGCCCGCATTCCTTCTCTGTCA
ATACAGCTATCCGTCAACTCTAGACGAAGTGCTCGAAGATCTAAAT---G
ATCAAATCCAAAGTTGTGATCATTTTCGGTTCTTATGGTTTCCGCACACC
AATTATGTTTCCATTTCAAACAACAGTAGAATTTCTAATAAAGAGATAAC
TAAATATTCCACATTCCAAACGGCTTATAATTGGTTT-------------
--------------TGGAATTACGGTATCGGATATTATTCGCTTGAATTC
GCGTATTGGGTCAGTACA---TATTGTCAACGATTAGTCCCATATATTAA
TCGCATCTACTTTTGGCTGCTTTAT------------TCAAAACCTCAAC
AAACAATTGATGTCAGCCATAAAGTC------TTCAATTTTGAATGTCTT
TTTAAACAACACGTCAATGAGTGGTCTATTCCTAATGAAAAGACAGCTAA
AGTATTGAATCAGTTAAAGAGTTGGATA---GAATCGACTCCAGAT----
---ATTTATGTT
CACTTTCCTGTTGAAGTCCGA-----------------------------
-------TTTGTAAAAGAAGATGATATTTACTTAAGTCCAGCATTTGGT-
--CGCAATTCG---------------------------------------
------------------------------------ACATTTATTAACAT
AATTATGTATAGGCCTTACGGTAAAGATGTTTCG---CATTCAGAATATT
GGGATCAATAC---GAGAAGATCATGAAAGAAGCCGGCGGTCGACCTCAT
TGGGCAAAAGCTCAT-----------------------------------
-------------------------------AGAGAAACTGCGACTGATT
TCGTAAAGATGTAT---CCATACTTCAGAGCCTGGTCTCACGTTAGAAAG
CGTTTGGATCCCATCAATATGTTAGTCAAT------TCATATCTCAACAG
A---ATTTTA--

>Dermatophagoides_farinae_(Acari)

----CGAATAGCAGTGGCTGGATATCAATTTAAAAATTGGTCCGGTACTT
ATCAATGTGAGCCAGAATTGTTTTTTATCCCAGAAACAATCGATGAAATT
CGTGAGATTCTCTATTTAGCCAAAACTGAAAAGAAACGAATCCGTGTAGT
TGGATGCGGTAATTCTCCCTCTGATCTATGTTGTTCATCTGATTATATGA
TATCGATGGAACGTTTTTCAAACATTCTTTATATT------------GAT
CGACAACAATGCCTTGTCACAGTCCAAGCCGGTATTCATCTTGATCGATT
GAATAAATTA---CTTGAAGAAAATGAACTAGCGTTGCCTGTTCTAGGTT
CAATATCGGAATCTGCATTGGCTGGAGTCATATCGGTTGGCACACACGGA
ACTGGTTATCAAACGGGCATCATAGCCGATTATGTGGTAGCTATGCGTTT
GATAACACCTTCAGGAGACATAATATCTTGTTCAAAAGAATCTGGCACTC
AACATAATGATGTCTTCAATTCTGTACTATGTGGCCTAGGCGCATTGGGT
ATCATATTAGAAGTCACTATTCGATGTGAGCCTAAATTCTTTTTACATCA
ACTAACGTATCCCAGCCGATTAGATTATGTACTTGATCGATTGGAT---G
AGAATGTTGAATCATGTGATCATTTCCGTTTTCTTTACTTTCCACATACC

```
GATTATGTTTCTGTTTCCATAACGAACAAAGTGAAAGAACGTGATATGCT
TCAG---------------AAAATTTTCGACTGGATC-------------
--------------ATTAAATATGGTGTTGGCTATCATCTACTTCAATTC
GCTTATTGGCTATCAACT---TATGCGCCGGCAATAGTGCCCACCATCAA
TCGATTAGCATTCTGGCTGCTTTAT------------TCATCAAATCAAG
TCCAAATGGACATTAGTTATAAAGTT------TTTAATTTCGAATGCCTG
TTCGCTCAACACGTCAACGAATGGTCCATTCCCAGGAACAAGACAAGTCA
AGTGTTACGCGAGCTAAAAGCAGCAATC---GATCATGGT---GGC----
-----------------------------------------CTATACGCA
CATTTTCCTATCGAAGTTCGA-----------------------------
-------TTTGTTCAACCATCTTCCATCTATCTTGCGCCAAGTTATGGT-
--CGTGATTCA---------------------------------------
------------------------------------ACATACATTAACAT
TATCTCATATCGACCATATGGACGACATGTTGAC---CATACCGATTATT
GGAATCGTTAT---GAAGAGATTATGAAACGAAACGGAGGACGGCCTCAT
TGGGCCAAGAATCAT-----------------------------------
-------------------------------CGAGAAACGGCCGTTGATT
TGGTTCGAATGTAT---CCGCATTTCCGATATTGGGCTCATATACGCAAA
AGATTGGATCCAAATCGACTATTTTTCAAT------TCATATCTTGATCG
A---ATCTTTTCAACATTTCCGACTAGCACAATAGCCACCAGTGACCATA
ATGAATCTAAAATGTTTGCTAACGTCCAAGGTGGTGATGGTAGTTTGGAT
TTGTCTCAATCTTTACAC--------------------------------
--------------------------------------------------
--------------------------------------------------
--------------------------------------------------
--------------------------------------------------
--------------------------------------------------
--------------------------------------------------
--------------------------------------------------
--------------------------------------------------
--------------------------------------------------
--------------------------------------------------
--------------------------------------------------
--------------------------------------------------
----------------
>Radix_auricularia_(Gastropoda)
--------------------------------------------------
----TCTCTTGGAAAGTCTGGTAAAAAGTTTACTAACTGGGCCACTACCT
ATTCTTGCACTCCGGAACTTTATTTTGAACCAGAAACAGAGGATGACATT
AAAAAGATTTTGGCCTATGCTCAAGAAAAAGACAAAAAAGTCAAGGTCGT
TGGTTTTGGCCACTCACCTTCTGATTTAGCCTGTACGTCTGATTATATGA
TTAGTCTCTGCAGGTTCAACAAGGTCATAAAGGTT------------GAC
AAAGAAAAATTGCAAGTAACTGTTCAAGGTGGCTGTCTTCTTAAAGAACT
GAATGATCACGTCCTACCAGCTAATGGGATGGCACTCTCTGTACAAGGGG
CTGTATGTGATTTGACTGCAGCTGGTGTTATTAGTACCGGCACTCACGGT
ACAGGTGCAGAGTTTGGTATCATTTCATCTTATGTTGTTGGTCTTGAGCT
AATGAAAGCTTCAGGGGAAGTAATTACCATCAACCAAGAA---------C
TCAACTCAGAATTACTTCCAGCTGTTTCTCTTAGTCTGGGCTCCTTGGGT
ATCATTCTATCTGTCACATTGCAGTGTGAGAAAGCTTTCAGGCTGCATTG
CAAACAAGTGACAAACAACTTGCAAAATGTGATTGAAAACCTTGAT---G
TGTATGTGACCAGTTCTGATCATTTCAAGTTTTTCTGGTATCCACACACA
GAATCTGTCGTTTGCTTTTTCACAAATAGAACAAAAGAGGAACCTAATGT
AAAG---------------------TCAAGCTGGTTC-------------
--------------TGGGAGATGGGAGTTGGTTACTATTTACTACAGATA
TTGTTA------TGTACT---TTTTTCCCCACTTTAGTTCCTGGCATCAA
CAGACTTTACTTAAAGCTCTTCTGT------------TCTAGAAGTGTAG
AGAGAGTAGACAGAAGTGATAAGATC------TTTAACTTTAATTGTTTG
TTCAAGCAGTACGTCATGGAATGGTCTATACCCAGGTCCAAAGTTGGAGT
```

AGTTCTGTGGGAACTGAAAGAGTGGATT---GAGAAAAAC---AAA----
---TTTCCGGCA
CATTTTCCCGTGGAAGTGCGT-----------------------------
-------TTTGTCAAGGGAGATAATATTTATCTGAGTCCTTGCTATAAA-
--CAAGATTCC---------------------------------------
------------------------------------TGCTATATTAACAT
TATTATGTACAGGCCATTCAATAAACTTGTGTCA---CATGAAACCTACT
GGAATGAGTAT---CAAAGTATAGTTGCCAAACATGGCGGAAGGCCACAC
TGGGCTAAGGACCAT-----------------------------------
-------------------------------AACTTTACAGGCACAGAAT
TCATCAAGCTATAT---CCAAAGTGGAATGAGTTTTGTGCCATCCGTGAA
AAAATGGACCCTAATGGGATGTTCCTCAAT------GCAAATCTGGAGAG
A---GTATTTAACAACAGACCTTCACCTTCAAGGAATCATGTA-------

>Aplysia_californica_(California_sea_hare)_Aplysiidae_XP_005103891.1_(Gastropoda)
---------------------------------ATGTCAAATTTTGATAT
TCGGACCCTCGGCAAGTCAGGTAGGCAGTTCACCAACTGGGCAACTACAT
ACTCATGTCAGCCAGAACTGTATTTTGAACCTGAGAATAATGAAGAAATA
AAGAATATTGTTGATTATGCACGAGAGAAGGGGAAAAAGGTGAAAGTGGT
TGGTTTTGGACATTCCCCTTCAGACATCGCTTGTACTACAGACTTTATGA
TCAGCCTGAAGCGCTACAATAAAGTTTTGAAGGTT------------GAT
GAAGAGAAGTCTCAGGTGAGCGTGCAAGCTGGATGCTTGATAAAAGACCT
CAACCACAACGTCCTTCCAGCGCACAACTTGGCATTCTCTGTCCAAGGGA
CTGTGGACGATCTCTCAGCTGCTGGTGTCATTAGCACTGGAACTCACGGG
ACTGGCACCAACTTTGGCATTGTGTCTTCCTATGTCGTGGATCTCACTCT
AATGAAAGCCTCTGGGGAGATAATCACCATCAGCAAAGAA---------G
AAAACTCTGAGTTGTTCAACGCTGCTGTTCTGGGTCTTGGCTCGCTTGGA
GTGATTCTCTCTGTCACGTTCCAGTGCGAGAAAGCTTACAGACTTCACCA
GAGGCAGTTCCCCAGCTCCCTGAAAAATGTCATTGACAACTTTGAC---A
AGCATGTGGCAGAGGCTGACCATTTCCGCTTCATGTGGTATCCTCACACT
GAGTCTGTCGTCTGTTACAGTGCCAGCAGGACCAAAAAGGAGCCAGCCGT
GAAG--------------------TCCAGCTGGTTT-------------
--------------TGGGATATGTTCGTTGGCTACCATCTCTTACAACTT
TTGCTGTGGATAAGCACG---TTTGTGCCTTGTCTGGTGAAGAGCATCAA
CCGTCTGTACTTTCGCCTCATGTGT------------CAGCAGCCCATCG
AGAAGGTGGCCCGCAGCGACCGGGTC------TTTAACTTCAACTGCCTG
TTCAAGCAGTATGTCACAGAGTGGGCCATACCCAAAGAGAACACGGGCGT
GGTGCTGTACAAGCTGAAAGACTGGATT---GAGCAGAAC---GGT----
---TTCTACGCA
CACTTCCCCGTGGAGGTTCGC-----------------------------
-------TTTGTGCGTGCTGATGACATCTACCTGAGCCCATGTTATCAA-
--CAAGACTCC---------------------------------------
------------------------------------TGCTATATCAACAT

TATCATGTACAGGCCCTTCAACAAGCTGGTGGAC---CACGAGTCGTACT
GGACGGCGTAC---GAAAGCATCATGGCTGAGCATGGAGGAAGACCACAC
TGGGCCAAGGCACAC-----------------------------------
-------------------------------AAGTACACAGGATTGCAGT
TCCAGAAAATTTAC---CCCAAGTGGAAAGAGTTTTGTGCCATCCGGGAA
AAGCTGGACCCTAACGGAATGTTCCTCAAC------GCCAACTTGGAGCG
C---GTGTTTGATGCCAGACCTTCTCCTTCCACCTACGTC----------

>Lottia_gigantea_(owl_limpet)_Lottiidae_ESO97787.1_(Gastropoda)
------------------------------------ATGACATTAGACAT
ACGGTCTTTAGGTAAACCGGGATATAAATTCACGAATTGGGCTACAACAT
ATTCATGTTACCCAGAACTTTACTTCGAGCCAGAAACTACAGACGAAATT
CGACAGATATTGGCGTTTGCCCAGAAGAAGGGCAAGAAAGTGAAAGTTGT
GGGGAATGGACATTCACCATCAGATTTGGCCTGTACTACAGACTTTATGA
TCAGTTTAAAGCGATATAACAGAGTTCTACAGGTC------------GAT
AAAGAATCAAGATTAGTACGTGTTCAAGGTGGCTGTCTAATAAAAGATTT
AAATGAAAGGATTTTACCGGCCAATGGTTTGGCTTTCACTGTGCAGGGAA
CCGTTAGTGATTTAACTGCCGCTGGTGTCATTAGTACAGGCACTCATGGT
ACTGGACATGATTTCGGGATCATTTCAACATTTGTCCGAGAGATCGAACT
GATGCAAGCATCTGGAGAAGTATTAACAATATCCAGGGATTTA------C
AGAACACGGATCTATTTCCAGCAGCCTGTGTCTCATTAGGTGCACTTGGT
GTTATATTGAGTGTTACATTTAAGTGTGAAGCAGCCTTCAATCTGGAACA
GACACAATTCCCCAATTCCCTGAACAATGTATTAGAGAATTTAGAC---G
AATATGTTACTGGATCTGACCATTTCCGCTTCTTCTATTTCCCATATACC
AACAGCACACTGTGCTTTAACGCTAATAGAACCTCTAAGCCTAAGAATGT
AAAA---------------------TCAAGTTGGTTT-------------
--------------TGGGAAACTTTAGTTGGTTATCACATTTTTCAATTT
TTCTATTGGCTAGGTGCC---TTTATAACTGCCTTAGTACCTGTAGTAAA
TCGATTGTTGTACAGGGTGATATAC------------GCACACAAGTCAG
AAAAAGTCGATAGATCTGATCATATA------TTTAATTTTAATTGTTTA
TTTAAACAGTATGTTTCAGAATGGTCCATACCAAGAGAGAAGACATCTTA
CGTTTTAAAAGAAATTCAGAAATGGATT---ATAGACAAC---AAT----
---TTCCCTGCT
CATGTTCCAATAGAAGTGAGA-----------------------------
-------TTTGTGAAAGGGGATGATCTTTATTTAAGCCCAGCTTATGGAT
GGAAGGATACA---------------------------------------
------------------------------------TGTTACATCAATAT
AATTATGTATACACCTTTTAATAAGATTGTACCA---CGAGAGAAATATT
GGAAAGCCTTT---GAAAAAATTATGTCCGATGTTGGTGGACGACCTCAT
TGGGCAAAGGATCAT-----------------------------------
-------------------------------AAATATGGACGAGAGGAGT
TTCAGAACCTGTAC---CCTCAGTTTGATACCTTCTGTAAAATCAGAGAA
CGTCTGGATCCTAATGGAATGTTCCTGAAC------TCCAACCTGGAACG
A---GTCTTTGGGAACAGTTCGAATTCCATCTTTAAAGTG----------

>Colubraria_reticulata_1_(Gastropoda)

-------CACGGGATACCAGGACACCAGTTCACCAACTGGAATGACACGT
ACTCCTGCCAGCCCGAGTTGTTCTTTGAACCCGACACCGTCCAACAGCTG
CAGCAGATTCTGGAAGCTGCTCGGAGGGCAGGGAAGAAAGTAAGGGTTGT
GGGCATGGGACACTCCTTCCACGATCTGCCCTGCACCACGGAGTACATGA
TTTCTCTCAGTCGCTACACCAAGGTCCTAAAGGTG------------GAT
GAGGGCCTGCAGCAGGTGACAGTACAGGCAGGTCTGGTGCTCAAGGACCT
GGTTAATGACGTGCTGCCCCCTCATGGACTGGCTCTTGGCGTGACAGGAG
CCATGCCACGGATCACAGTGGGAGGTTTCATGTCTACTGGCACACATGGC
AGCGGAATGAAGTATGGCACCATGTCTTCTCTGGTGGTTTCCCTTGACCT
GCTTACGGCATCAGGGGAGATCATGCACATCTCCAAGGAG---------G
AGAACTCTGATGTGCTGGAAGCTGCGCTGTTGAGCCTGGGGACGCTAGGC
GTGGTGCTGACGGTCACGCTGCAGTGTGAGTCTGCCTTCACCGTCCGGCA
ACGCCAGTACCCTGCCACGCTTGACTACGTGTTGGACAATCTACCT---G
AGCTGCTGGACGCCAGTGACTATTTCCAGTTCTGTTATTTCCCGCATACA
GACGGTGCAGTCTGCTACCAGGCAACTCGCACAAAAGAGGAACCCAACGA
TGTG---------------------AGCAGCTGGTTCTGGGACGTG----
-----------------------TTCATTGGGATCCACGTCTTGCAGCTT
CTGCTCTGGATATGCACA---TTCATTCCTCGGCTGACGCCGTTGCTCAA
CAATCTGTTTTACCGCTTCGTGTTC------------CATCGGTCAGTGG
ACGTAGTGGACAGTGCTCATCGCGTG------CTGTGTCACGACTGCCCC
ATCCCTCTTAATGCCTGTGAATGGGCTGTGGCCAAAGAGCACACAGGCCC
CGCACTGAGAGAGCTGCAGCGATGGGTC---ACCCAAAGT---GGA----
---GGCCCTGCC
ACGTTTCCTGTGGAGGTACGC-----------------------------
-------TTTGTGAAGGGAGAGACCATCTACCTGAGCCCTGCCCACGGA-
--AGGGACACC---------------------------------------
------------------------------------TGCTACTTCAGCAT
CTTCTACTACAGACCTTACAACAAAGATGTACAT---AACGACAAGTACT
GGGACAGCTTT---CAGCGAATCATGGCTTCCTTCGGTGGACGACCTCAC
TGGGCCAAGGACCAC-----------------------------------
-------------------------------AAGTACGGGTGCCAGGACT
TTGAGAAGGTGTAC---CCCAAGTGGGGCGAGTTCTGCGCCATCCGGAAG
CGGATGGACCCCTCCGGCATGTTCCTCAAT------GATCACCTGCAGCG
T---GTCTTCCTCAGCAAGCCCTCCCCCGTGGCCACTACGACGGCTGTT-

>Colubraria_reticulata_2_(Gastropoda)

--------------------------GTGCTGAGCCTGGGGGCGCTGGGA
GTGGTTCTGACGGTCACCATGCAGTGTCAGCCTGCCTTCACCCTGCGACA
ACACCAGTACCCCGCCAAGCTGAAAGACGTGATAGAGAATGTGAAT---G
TGTATGTGACAGCCAGTGATCATTTTCGGTTCTTCTGGTTCCCACACACG
GACGACGTCATCTGCTATCATGCTTCTCGTACCAAACAGGAGCCCATGCT
GCAA---------------AAATCAAGCGGCTGGTTC-------------
--------------TGGGACACGTTTGTGGGATTCTATGTTTTGCAGCTG
GTGTATTGGATCAGCACG---TTGATTCCTAGTCTGGTGCCGGCCATCAA
CCGGTTTTACTACCGCGTGTTGTAC------------GCCCACAGCGTGG
AGAGGGTCGATGTCAGCCATAAGATC------TTCAGCTTCAACTGTCTC
TTCCGCCAGTACGTCACGGAGTGGGCCATTCCACGAGAGAAAGTGGGGGT
CGTTCTGGTGGAACTGAGCAGCTGGATC------TCCAACAACAGC----
---TTCCCGGCA
CATTTCCCAGTGGAGGTGCGC-----------------------------
-------TTCGTCAAAGAGGATGACATCTACCTCAGCCCCGCCTATGGC-
--AGAGAGTCC---------------------------------------
------------------------------------TGCTACATCAATAT
CGTCATGTACAGACCGTTCAACAAGTACATCCCC---AATCAGAAGTACT
GGGACGCCTTT---GAACGCATTATGTTGTCAGCAGGCGGGCGACCCCAT
TGGGCCAAGGACCAC-----------------------------------
-------------------------------AATCTGAGGGGGCAGGACT
TCGAGAGGCTGTAT---CCAAAGTGGGGGGAGTTCTGCCGACTGCGGGAA
CAGCTGGACCCCAACGGGATGTTCCTCAAC------GCCAACCTGGAACG
C---GTGTTT--


```
----------------
>Lymnaea_stagnalis_(Gastropoda)
--------------------------------------------------
----TCCCTTGGAAAGTCTGGTAAGAAATTTACCAACTGGGCCACAACAT
ATTCTTGCACTCCAGAGCTTTATTTTGAGCCAGAAACAGAGGATGAGATT
AGAAAGATTTTAGCCTATGCTCAAGAAAAAGACAAGAAGGTCAAGGTTGT
TGGTTTTGGCCACTCACCTTCTGATATAGCCTGTACAACTGATTTTATGA
TAAGTCTCTGCAGGTACAACAATGTCATAAAGGTT------------GAC
AAAGAAAAATTGCAGGTGACAGTTCAAGGTGGCTGCCTTCTTAAAGAACT
GAATGATCATGTCCTACCAGCCAATGACATGGCACTCTCTGTGCAAGGGG
CTGTATGTGATTTGACTGCTGCTGGAGTTATCAGCACAGGGACTCATGGG
ACAGGAGCACAGTTCGGCATAATTTCATCTTACGTTGTTAATCTTGAGCT
AATGAAAGCTTCAGGGGAAGTGATTACTATAAACGAAGAG---------G
TCAACTCAGAATTGCTACCAGCTGCTGCTCTCAGTCTGGGCAGCTTGGGT
GTAATTATCTCTGTCACATTGCAATGTGAGAAAGCCTTTAGGTTGCATTG
TAAACAAGTCACAAACACCTTGCAAAATGTAATAGAAAACCTTGAT---G
TCTATGTAACCAGCTCTGACCATTTCAAATTCTTCTGGTATCCACATACA
GAATCTGTTGTTTGCTTCAACACTAATCGAACAAAAGAGGAACCCAAAGT
AAAG---------------------TCCAGCTGGATC-------------
--------------TGGGATATGGGAGTTGGCTACTATTTACTACAAATA
TTC------------ACT---TTTTTCCCAAGTTTAGTCCCTAGTATCAA
CAGACTGTACTTCAAGCTCTTCTGT------------TCTAGCTCTGTGG
AAAGAATAGACAGAAGTGATAAGATC------TTTAACTTCAACTGTTTA
TTCAAGCAATATGTCATGGAGTGGTCTATACCCAGGAACAAAGTTGGAGT
TGTTCTTTGGGAGTTGAAGGAGTGGATT---GAGAAAAAT---AGA----
-----------------------------------------TTTCCTGCA
CATTTTCCTGTGGAAGTTCGT-----------------------------
-------TTCGTCAAGGGAGACAATATTTATCTGAGCCCTTGCTATGAT-
--CAAGATTCT---------------------------------------
------------------------------------TGCTATATAAACAT
TATTATGTACAGGCCATTTAATACATTGGTGTCA---CATGAAACCTATT
GGAATGAGTAT---CAAAGTATAGTTGCCAAACATGGGGGAAGGCCACAC
TGGGCTAAGGACCAT-----------------------------------
-------------------------------AATTTCACAGGCACAGAAT
TTATCAAGCTATAT---CCAAAGTGGAATGAGTTTTGTGCTATCCGTGAG
AAGATGGACCCTAATGGTATGTTTCTCAAT------GCAAATCTGGAGAG
A---GTATTCAACAGTCGGCCTTCACCTTCAAGGAATCATGTG-------
--------------------------------------------------
--------------------------------------------------
--------------------------------------------------
--------------------------------------------------
--------------------------------------------------
--------------------------------------------------
--------------------------------------------------
--------------------------------------------------
--------------------------------------------------
--------------------------------------------------
--------------------------------------------------
--------------------------------------------------
--------------------------------------------------
--------------------------------------------------
----------------
>Kudoa_iwatai_(Myxozoa)
--------------------------------------------------
----------------ATTGATTATATATATCGAAATTGGGCTGGTACCT
ATTTCTGCAAACCCCAATTATATTTTCAACCTGAAACTATCGATCAAATA
TTAGAAATATATGATCTTGCGAAACTATATGGAAAGAATATAAGGTTTAT
AGGAAGTTCAGCTTCTCCATCCAATCTACCGTGTACTAATGGATATATGG
```

TTTGTATGAAAAAAATTTAATAAGATTCTATGTATT-----------GAT
AAAGAAAAGTTAGAGGTAAAAGTACAAGGGGGTATTATATTATCTGAATT
ACAGGAAATA---CTCTATAGAAATGGCTTGGCTATATCTACTTTACCAT
CAATCAATTTTATAACACTTGCAGGAGCAATATCAACAGGAACTCATGGA
AATGGACTTAAACATGGCAATCTTTCATCAATGGTTTTAAGCATAGAGAT
TTTATCTCCAAATGGACAACTTTTGTATTTGGATTCTAAC---------A
ACAATAAAGAGTTATTTCTTGCTGCTAAAGTTTCCCTTGGTCTTCTAGGT
GCTATTATTAATGTTACACTACATGTTGAACACTGTTTTAATGTAATCGA
AAAAAAATATCCATGCACTTTTGATTATATGATCAATAACATTGAT---A
ATATAATAAATGCTAGTGAATTCAGTAAAATAATGTTCTACCCTCACACA
GATAAATGTGTTATATTTGAAATGGATAGAACAGATAGAAAAGAATCTCC
ATCA------------------------TCATATATC------AGT----
------------------------ATTTATGGATAAAATTATTGGAAAAG
TCTTGTTTTATTACCTGCTGTCGTATACCCTCCATAACTAAGTACATTAA
TAATCTTTTCTTTTACATCCTCCAT------------AATAAAATTAGTG
AACGTATAGACAGGAGCGATTGTATA------TTTAATTTTGAAGTGTAT
TTTCCCCAGTATGTAACAGAAATTTCTTTTAAAATTGAGGATACTAAAGA
GATTTTGGAAGAATTTTATAATCTTATA---AAGCAGCAC---AAA----
---CTTAATGTG
CATTTTCCTGTTGAAATACGA-----------------------------
-------TTTGTAAAAGCAGATGATGCTTATCTCAGCCAAAATAATGGT-
--TACGACTGT---------------------------------------
------------------------------------ATGATGATAAACTT
ATTGGCCTTCCGACCAGGACAATCCCATTTTCCT---AAAGAAGAATATT
TCACACTTTTTGATGAAAAGATACGTAAAAAATTCAATGGTAGATCTCAT
TGGGCTAAGACGCAT-----------------------------------
-------------------------------ACTGATAATCCAGCATCTC
TACAAACCTTATAT---CCAGATTTTGGAAAATTTTTGACAGCAAGAGAG
TTCCTTGATCCAGATGGTGTTTTGCTTAAC------GATTATGCTAAAAT
ACACCTTTTTGGTAAAAAAATTAAA-------------------------

>Steganacarus_magnus_(Acari)_partial

---TTAGGTT
CGATATCAGAGCTTACAATTGGCGGAACAATTAGTACTGGAACTCACGGA
ACGGGCGCTAACTTTGGCATAATGTCTTCATACGTGATCGATGTTGAAAT
GATTACTTCTTCCGGAGATACCATCAAATGTTCGCGCGAC---------G
AAAACAGTGATATATTTTTGTCAGCGATGTGTGGTTTGGGTGCTATCGGT

ATAATAACGAAAGTGTCGTTGCAATGCGAGCCATCATTCCTTCTTCATCA
GTACATATATCCATCCACATTAGATGACGTTCTCACTGATTTGAAT---G
ATCAGATCAGGAGTTGTGATCACTATAGGTTTCTATGGTTTCCTCATACG
AACTGTGTGTCAATTTGGCAAAACAGTAGAATTTATAACAAATCAATTAC
TAAATACTCTACATTTCAACAAATGTATAATTGGTTC-------------
--------------TGGGATTACGGCGTCGGTTATTATACGCTAGAGTTT
TGGTATTGGATGAGTACT---TTTGTGCCATCATTAGTGCCATATATTAA
CCGAATCGCGTTTTGGTTGCTTTAT------------TCCAAACCAAAGG
AAACTGTGGATATAAGTCACAAAATA------TTTAATTTCGAGTGTCTT
TTTAAGCAACACGTCAATGAATGGTCGATTCCTGTTGAAAAGACGGCTAA
AGTATTGCTAGAGTTAAAGCAATGGATT---GACACGACGCCTAAT----
---GTTTACGCG
CACTTTCCAGTTGAGGTACGT-----------------------------
-------TTCGTAAAGGCTGATGACATTTATTTAAGTCCAGCGTATGGC-
--CGCGATTCT---------------------------------------
------------------------------------ACTTTTATTAACAT
CATTATGTATCGACCGTTTGGCAAAGATGTTCAC---TATCAGGATTATT
GGAACCAATAT---GAAAAGATTATGAAAGAATCTGGTGGTCGACCACAT
TGGGCAAAAGCTCAT-----------------------------------
-------------------------------AGAGAAACTGCAGCTGATT
TTGTTAAGATGTAT---CCGTATTTCAGAGCGTGGATACACGTTAGAAAA
AGAGTTGATCCCATGAATATGTTTATGAAT------CAATATCTTGAGAG
A---ATTTTTTTAAAATACCCGATTGATGGACATAAT-------------

>Ixodes_scapularis_(Acari)_partial

----ATTTCGGCTATCAAAGGGGTGACGTTCGAAAACTGGTCTAAAACGT
TTTCCTGCAAGCCCGAGTACTTGTTTGCTCCCAGAAGCAAAGATGAATTA
TGTGAGGTGCTGGAGTTTGCCCTCCAACGTAGCATGAAGGTCCGCGTGGT
TGGTGCTGGTTATTCCCCGTCAGACATTGCATGCACCTCGGATGTGATGA
TCAGCATGCGCAGCATGGACAAGGTGCTTGAGGTC------------AAC
AAAGAAAAGATGACTGTCAAGGCGGAAGCTGGGGTCACTCTGAAAAGGCT
GAACGAAGTCGAACTGGCCAAGAACGGACTCGCCCTT------CTGGGTG
CCGTGTCGGAGATCACGCTTGGCGGTGCAATCGCCACCGGCACTCACGGA
TCGGGACTCAATTTTGGAATCCTGTCAACGCAGGTGTTGGAGCTGGAACT
GGTGACTTGTCTGGGCAAGACGCTGGTCTGCTCGCGGGAG---------A
GCAACCCGGAGGTCTTCCTGGCAGCCGCCTGCGGGCTCGGGGCCATCGGG
GTGGTCGTCGCGGTCACCGTGCAGTGCGAGCCAGCCTTTCGGCTGCGCGA
GTCCCGCTACTCCTGCGCCTTGCAGCGGGTGCTGGAAAACCTGGAC---G
TGCACCTGAAGAGCTCAGACCACTTTCGCTGCCTGTGGTATCCGCACACG
GACACCGCCGTCTGCTTTCATCTGACTAGGACTGATGAGCACAAGCACAT
ACTT---------------------------CATTTT-------------
--------------TATTCATACCACTGCAAATATTATATGTTATGTTTC
TTCTGTTGGGTG---------------CCACCCCTGGTGCCGTGGCTGAA

```
CCGACTCTTCCTGTGGGCGGTGTTT------------GCGCCGCCGAAGC
AGCGGGTGGACCTGAGCCATCGGGTG------TTCAACTACGAGTGTCGC
TTCAAGCAGCATGTCAACGAGTGGTCCATCCCAAGGCAAAAGACTGCCGT
GGCTCTGTGGAAACTCAAAGAGTGGATC---GACAACACACCGGAC----
-----------------------------------------ATGTACGTG
CATATTCCAGTGGAGGTTCGG-----------------------------
-------TTTGTGCGCCAGGACGACATCTTCCTCAGCCCAGCGTGTGGA-
--CGTGACTCG---------------------------------------
------------------------------------TGCTACATCAACGT
CATCATG------CCTTACGGTAGGACGGTCCCC---CACGAGCGTTACT
GGGCAGCCTAC---GAGGGAATCATGAGAGGCCTTGGCGGCAGACCTCAC
TGGGCCAAG-----------------------------------------
--------------------------------------------------
--------------------------------------------------
--------------------------------------------------
--------------------------------------------------
--------------------------------------------------
--------------------------------------------------
--------------------------------------------------
--------------------------------------------------
--------------------------------------------------
--------------------------------------------------
--------------------------------------------------
--------------------------------------------------
--------------------------------------------------
--------------------------------------------------
--------------------------------------------------
--------------------------------------------------
--------------------------------------------------
--------------------------------------------------
----------------
>Euroglyphus_maynei_(Acari)_partial
--------------------------------------------------
--------------------------------------------------
--------------------------------------------------
--------------------------------------------------
--------------------------------------------------
--------------------------------------------------
--------------------------------------------------
--------------------------------------------------
--------------------------------------------------
---------------GGTATCATAGCCGACTATGTCATGGCTATGCGTTT
GATAACACCTTCCGGAGATATAATATATTGTTCAAAAGAGACTGGCAGTC
AGCATTCTGATGTGTTCTTTTCGGCGTTATGTGGCCTGGGCGCACTTGGT
ATTATATTGGAAGTAACCATCCAATGTGAACGTAAATTCTTTCTTCATCA
GCTAACTTATCCGAGTCGATTAGATTCTGTATTGGAACGATTGGAT---G
AGAATGTTGAATCATGTGATCATTTTCGTTTTCTTTACTTTCCACATACC
GATTATGTTTCCGTTTCCATAACGAACAAAAGGATA--------------
-------------------------AGTGATTGGATC-------------
--------------GTCAACTATGGTATTGGTTATCATCTACTTCAATTT
GCCTATTGGTTATCGACT---TATGAACCTAAAATAGTGCCCATCATCAA
TCGATTAGCATTCTGGCTACTCTAT------------TCATCCAATCAAG
TCAAATTGGACATTAGTTATAAAGTA------TTCAATTTTGAATGTCTA
TTTGCTCAACACGTAAACGAATGGTCAATTCCA------------AGTGA
AGCGTTACTCGAACTAAAAACAGCAATC---GATAATGAT---GGC----
-----------------------------------------CTATACGCA
CATTTTCCGATCGAAGTTCGA-----------------------------
-------TTTGTTCGACAATCATCCATCTATCTAGCGCCAAGTTATGGT-
```

```
--CGTGATTCC---------------------------------------
------------------------------------ACATACATTAACAT
TATCTCATATAGACCATATGGACGACATGTTGAT---CACACCGATTATT
GGAATCGATTT---GAAGAGATTATGAAACGAAACGGTGGCCGACCTCAT
TGGGCTAAG-----------------------------------------
--------------------------------------------------
--------------------------------------------------
--------------------------------------------------
--------------------------------------------------
--------------------------------------------------
--------------------------------------------------
--------------------------------------------------
--------------------------------------------------
--------------------------------------------------
--------------------------------------------------
--------------------------------------------------
--------------------------------------------------
--------------------------------------------------
--------------------------------------------------
--------------------------------------------------
--------------------------------------------------
--------------------------------------------------
--------------------------------------------------
----------------
```
